# Graphitic carbon nitride/nickel dual catalysis for decarboxylative synthesis of unsymmetrical ketones from keto acids

**DOI:** 10.1039/d5gc03641k

**Published:** 2025-10-24

**Authors:** Michael T. Findlay, Florian Lukas, Francesca Rizzo, Junsong Liu, Benjamin Martin, Simon Allmendinger, Markus Furegati, Pablo Gabriel, Timothy Noël

**Affiliations:** a Flow Chemistry Group, Van ‘t Hoff Institute for Molecular Sciences (HIMS), University of Amsterdam Science Park 904 1098 XH Amsterdam The Netherlands t.noel@uva.nl; b PhotoGreen Lab, Department of Chemistry, University of Pavia Viale Taramelli 12 Pavia 27100 Italy; c Novartis Pharma AG Fabrikstrasse 4002 Basel Switzerland

## Abstract

We report a sustainable, dual-catalytic system for the synthesis of ketones *via* decarboxylative cross-coupling of α-keto acids and aryl halides, enabled by graphitic carbon nitride (gCN) and nickel catalysis. The heterogeneous gCN photocatalyst, derived from earth-abundant precursors, facilitates the generation of acyl radicals under light irradiation, which undergo efficient nickel-catalysed cross-coupling to form a diverse array of ketone products. The methodology demonstrates broad substrate scope, including heteroaryl and vinyl halides, and is compatible with a range of functional groups. Our studies revealed and addressed a competing Norrish Type II cleavage pathway, enabling the successful coupling of longer-chain substrates by switching to a 456 nm light source. Oxamic acids were shown to participate under similar conditions to furnish amides. Compared to iridium-based systems, this protocol significantly reduces total carbon release (TCR), and the gCN photocatalyst was readily recycled over 10 runs without loss of activity. This work highlights gCN's potential as a green, reusable alternative for metallaphotoredox cross-couplings.

## Abstract 2

Sustainable dual-catalysis with graphitic carbon nitride (gCN) and nickel for ketone and amide synthesis from α-keto and oxamic acids.

## Graphical abstract

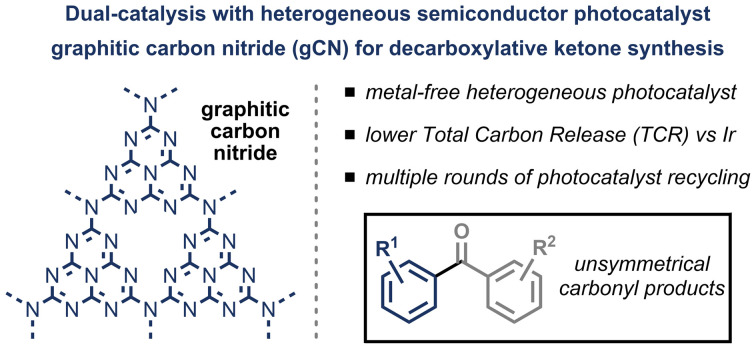

**Green foundation**

1. Carbon footprint: the use of metal-free gCN photocatalyst, which is formed from bulk chemical precursors, results in a significant reduction in carbon footprint as measured by TCR, making the process far more sustainable compared to variants using traditional photocatalysts.

2. Photocatalyst separation and recycling: facile removal of the gCN by centrifugation or filtration enables easy workups and recycling of the photocatalyst. No consideration has to be given to photocatalyst decomposition products or noble metal poisoning in the crude product.

3. Future work should focus on methods for recycling the nickel catalyst, and on catalyst immobilisation for use in continuous-flow photochemistry systems.

## Introduction

The emergence of photoredox catalysis in synthetic chemistry has brought a plethora of new synthetic methodologies to the modern chemist's toolbox, enabling a range of novel synthetic disconnections.^1,2^ In particular, metallaphotoredox chemistry, the merger of transition-metal catalysis and photoredox catalysis, has allowed the novel synthesis of previously elusive molecular scaffolds and the utilisation of a wide variety of potential coupling partners.^3^

Many existing metallaphotoredox strategies employ transition-metal complexes as photocatalysts, where the redox and excited-state properties can be modulated by variation of the ligand environment around the metal centre, making them a popular choice for reaction development.^4^ However, these photocatalysts are typically based on the platinum-group metals iridium and ruthenium, two of the least abundant stable elements found in the earth's crust.^5^ The scarcity of these metals has historically resulted in high and volatile prices, and coupled with a rapidly growing global demand, has led to a hesitancy to commit to their long-term use. Recent EU legislation has designated platinum group metals as critical raw materials, highlighting both their strategic importance and the urgent need to shift toward more sustainable and less resource-dependent alternatives.^6^ Additionally, cost and scarcity are not the only factors to consider – the large carbon footprint associated with mining these elements is often overlooked, with an estimated 8860 kg CO_2_-equivalent emissions associated with production of just 1 kg of iridium metal.^7^

Consequently, recent research has focused on the development of alternative photocatalysts as long-term replacements.^8^ In the last few years, heterogeneous semiconductors have emerged as a promising alternative class of photocatalysts that are capable of performing in photoredox and metallaphotoredox chemistry.^9–11^ In particular, graphitic carbon nitrides (gCNs) have been reported to facilitate a wide variety of synthetic transformations, including C–H functionalisations, oxidations, and metallaphotoredox C–O, C–N, and C–C couplings, providing a robust, noble–metal free photocatalyst that is prepared from cheap and abundant precursors.^12–21^ First reports of coupling between carboxylic acid and aryl halide coupling partners using gCN–nickel dual-catalytic systems, from the research groups of Pieber and Vilé, led exclusively to direct C–O bond formation and ester products.^22,23^

gCNs, which possess a relatively oxidising valence-bond position,^24^ have also been demonstrated to facilitate decarboxylative transformations with carboxylic acid substrates. Single-electron oxidation by the excited state photocatalyst is rapidly followed by decarboxylation, generating an alkyl radical intermediate which is then capable of reacting with a variety of potential coupling partners in order to form C–H, C–C, C–O and C–N bonds.^25–27^ Recent work in our research group has demonstrated the use of a gCN/nickel dual-catalytic system in forming C(sp^2^)–C(sp^3^) bonds from carboxylic acids and aryl halides, in a decarboxylative fashion.^28^ The research groups of MacMillan, Li, and others, have shown that acyl radical intermediates can also be generated in similar fashion *via* photoredox chemistry, leading to valuable carbonyl-containing products when combined with transition-metal catalysis.^29–31^

**Scheme 1:**
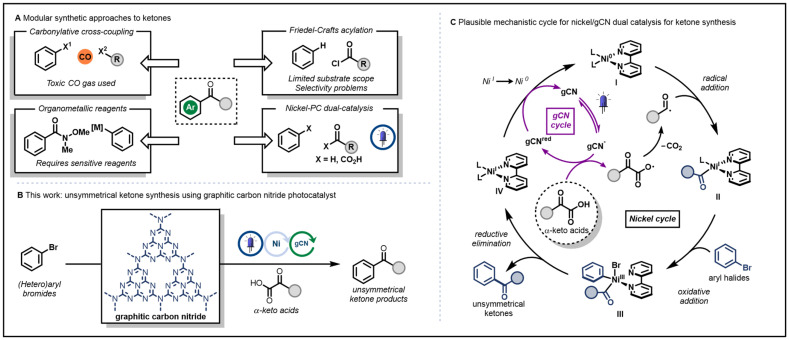
(A) Current approaches reported for modular synthesis of ketones; (B) our work on the synthesis of unsymmetrical ketones from aryl halides and α-keto carboxylic acids using gCN photocatalyst; and (C) proposed mechanism for the transformation. L = ligand, and can represent carboxylate, solvent molecule, phthalimide.

Among these products, ketones are particularly significant due to their broad utility and prevalence in chemical synthesis. They are ubiquitous motifs in chemistry, and feature prominently in the skeleton of a wide range of bioactive molecules – in many cases enhancing their effectiveness and selectivity – and serving as a versatile handle for further functionalisation. Traditional methods for their modular synthesis rely on highly-reactive organometallic reagents, hazardous carbon monoxide gas, or poorly-selective Friedel–Crafts chemistry (Scheme 1A). More benign methodologies have been reported using metallaphotoredox chemistry, most commonly employing iridium-based photocatalysts (Scheme 1A).^29,32^

In an effort to expand upon our earlier work, we investigated the use of α-keto carboxylic acids as a source of acyl radicals with a commercial gCN photocatalyst, furnishing ketones after nickel-catalysed coupling with aryl halides, providing a valuable alternative to the use of iridium-based photocatalysts (Scheme 1B and C).

## Results and discussion

We began our investigation by exploring the reaction between methyl 4-bromobenzoate 1 and phenylglyoxylic acid 2, which gave the desired biaryl ketone product 3 in a promising initial yield of 47%. After a thorough screening of reaction conditions, we were able to optimise our methodology – generating biaryl ketone 3 in an improved yield of 81%, by using commercial gCN photocatalyst (commercially available, for further details see SI), phthalimide as additive, NiBr_2_·glyme, and 2,2′-bipyridine as ligand, under irradiation with a 390 nm Kessil lamp for 16 h, using a 3D-printed UFO reactor developed by our group (Table 1, entry 1).^33^

**Table 1:** Reaction condition optimisation

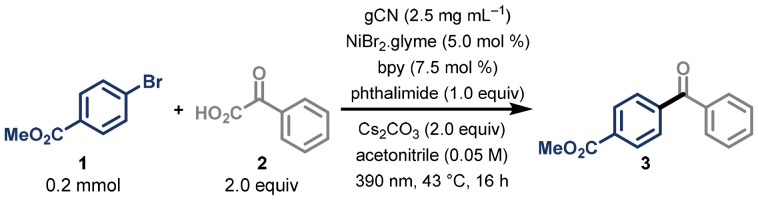		
Entry	Variation	3 (%)
1	None	81 (75)
2	K_2_HPO_3_/K_2_CO_3_/DBU as base	61/72/47
3	DMSO/DMF as solvent	75/16
4	Ar–I/Ar–Cl	81/0
5	1, 2 and Cs_2_CO_3_ (1 : 1 : 1) stoichiometry	53
6	No phthalimide	25
7	No photocatalyst	0
8	No nickel	0
9	No light	0

In the course of our investigation, we found that the diphenyl-substituted bipyridyl ligand (dPh-bpy), which we found vital for supressing esterification in earlier projects, was no longer necessary for optimal reactivity – with the unsubstituted bipyridyl ligand outperforming it under our conditions.^28^ Cs_2_CO_3_ was found to be the optimal base in our system, with other common bases K_2_HPO_3_, K_2_CO_3_, and DBU leading to lower overall yields (entry 2). The use of acetonitrile as reaction solvent led to the highest yield, with DMSO showing a slightly reduced yield of 3 of 75%, and the use of DMF resulting in a significantly reduced overall yield of 15% (entry 3). Aryl iodides could also be used as electrophilic coupling partners, but switching to aryl chlorides shut down reactivity (entry 4).

For valuable α-keto carboxylic acid coupling partners, it was possible to reduce the equivalents needed, with an equimolar amount of 1, 2, and base still leading to a synthetically useful yield of 53% (entry 5), with phthalimide proving to be an essential additive for effective trapping of the formed acyl radical on nickel – in its absence an increased amount of aldehyde byproduct was observed, generated through hydrogen abstraction by the intermediate acyl radical (entry 6). Control experiments conducted in the absence of gCN, nickel-catalyst, or visible-light irradiation confirmed the necessity of each component, and resulted in the quantitative recovery of aryl bromide starting material (entries 7–9).

**Scheme 2:**
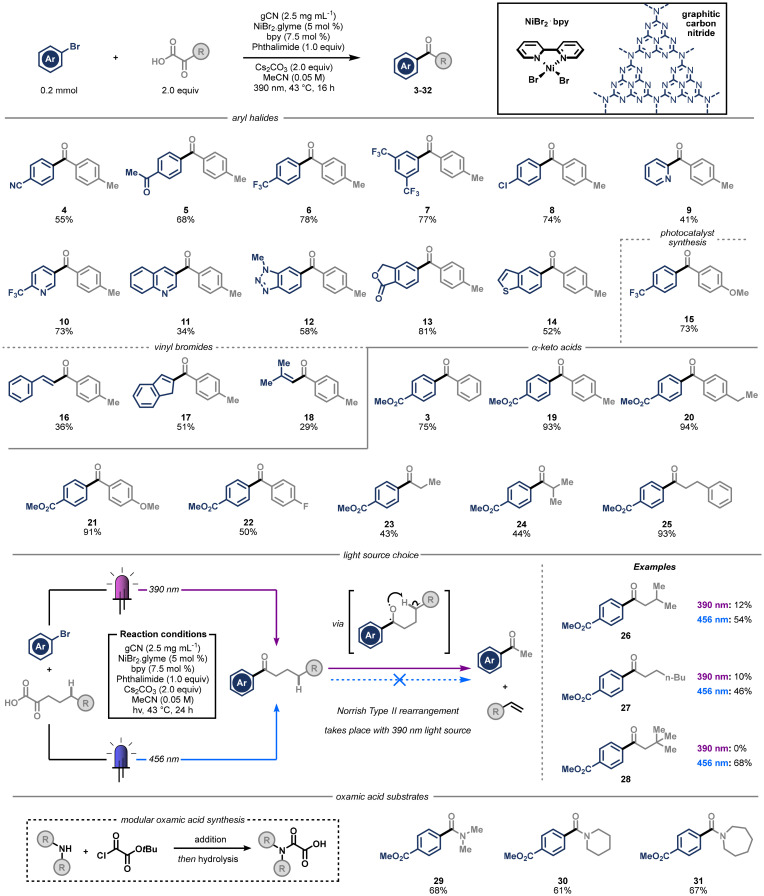
Scope of reaction, investigating (hetero)aryl halides, aryl and alkyl α-keto carboxylic acids, and oxamic acids. All yields are of isolated compounds.

Having found optimal conditions for efficient coupling between the acyl radical and the electrophile, we turned our attention to exploring the full scope of possible coupling partners (Scheme 2). We were pleased to find that besides the electron-withdrawing ester group (3), nitrile and ketone (4 and 5, respectively) groups at the *para*-position to the bromide were also well tolerated. This was an especially gratifying result, as these functional groups would typically interfere with conventional organometallic reagents. Both trifluoromethyl (6), and bis-*meta*-trifluoromethyl (7) substitution led to high yields (78% and 77%), and halogenated coupling partner 1-bromo-4-chlorobenzene generated product 8 in 74% yield, with complete selectivity for reaction at the brominated position. Additionally, due to the frequent appearance of heterocyclic motifs in active pharmaceutical ingredients, we set out to test the compatibility of heteroaryl bromide substrates in our system. We found that the protocol works with 2-bromo pyridine (9, 41%) and by switching to the more electron-poor 5-bromo-2-(trifluoromethyl)pyridine, the target product 10 was obtained with an improved 73% yield. Alternative N-heterocyclic scaffolds such as quinoline (11) and *N*-Me-benzotriazole (12) were amenable to the conditions, and products were also formed from oxygen containing phthalide (13) and sulphur-containing benzothiophene (14) structures. To showcase the utility of this method, we synthesised 15, a photocatalyst known for its use in hydrogen atom transfer chemistry, in a single step starting from commercially available starting materials, in an excellent yield of 73%.^34,35^ Furthermore, the protocol could be applied to vinyl bromides, leading to the synthesis of α,β-unsaturated ketones. Both linear (16) and cyclic (17) alkenyl bromide scaffolds could be coupled, in addition to unconjugated bromo-methyl propene (18), and generated the desired products in synthetically useful yields.

Next, we explored the range of α-keto carboxylic acid coupling partners that perform well under our reaction conditions. Electron donating alkyl- and methoxy-substituents at the *para*-position on the α-keto carboxylic acid led to increased product yields, generating products 19, 20, and 21 in 93%, 94%, and 91% yield respectively, whereas electron-withdrawing fluorine substitution decreased the yield slightly to 50% (22). Further increasing the electron-withdrawing nature of the *para*-substituent led to diminished yields, with a nitrile substitution generating the ketone products in only 10% yield (for unsuccessful substrates see SI section 13). Gratifyingly, the procedure was scaled up to 5 mmol with only minor adjustments to the reactor setup and base loading (1.0 equiv. instead of 2.0), at virtually no loss of yield (19, 77%).

Switching to alkyl α-keto carboxylic acids, we found that ethyl, isopropyl, or phenylethyl motifs were competent coupling partners, generating products 23, 24, and 25 in good yields. These substrates are particularly useful for performing downstream reductions to the alcohol in a stereoselective fashion, where an increase in the size differential between the two sides of the ketone can drive selectivity. Interestingly, when we subjected longer chained α-keto carboxylic acids to our reaction conditions, we were surprised to find efficient formation of undesired methyl ketone product. After investigation of the mechanism of this pathway, we were able to elucidate its origin as a Norrish type II cleavage of the product containing hydrogen in the γ-position, *via* direct irradiation with 390 nm light, as recently reported by the Wu group in their elegant protocol for alkene synthesis.^36^ To prevent this, we attempted to limit such decomposition by using shorter reaction times, which only led to mixtures of products. Instead, we found that suppression of the cleavage was possible by switching to a 456 nm light source, delivering products 26, 27, and 28 in good yields, and effectively shutting down the cleavage pathway.

Finally, to expand the utility of this reaction system, we next considered the use of oxamic acid coupling partners. These substrates can be prepared from amines *via* a simple addition–hydrolysis sequence starting from oxalyl chloride, providing easy access to a wide variety of potential coupling partners from the respective amines (see SI section 11 for details). Previous reports have demonstrated that this class of substrates can also undergo oxidative decarboxylation, liberating carbamoyl radical intermediates that can furnish amides in a modular fashion.^31^ Indeed, when using graphitic carbon nitride as photocatalyst, the reaction proceeded as expected, and we were able to produce acyclic and amide 29 and lactams 30 and 31 in excellent yields. In an attempt to further broaden the scope of the reaction we subjected an oxalate coupling partner (2-(benzyloxy)-2-oxoacetic acid) to the same condition, expecting to observe either single decarboxylation and ester products or double decarboxylation and C–C bond formation (see SI section 13).^37^ Unfortunately, this only led to the return of aryl bromide starting material, probably due to the more challenging oxidation of alkyl oxalates, which are likely out of the range of our gCN catalyst.

**Scheme 3:**
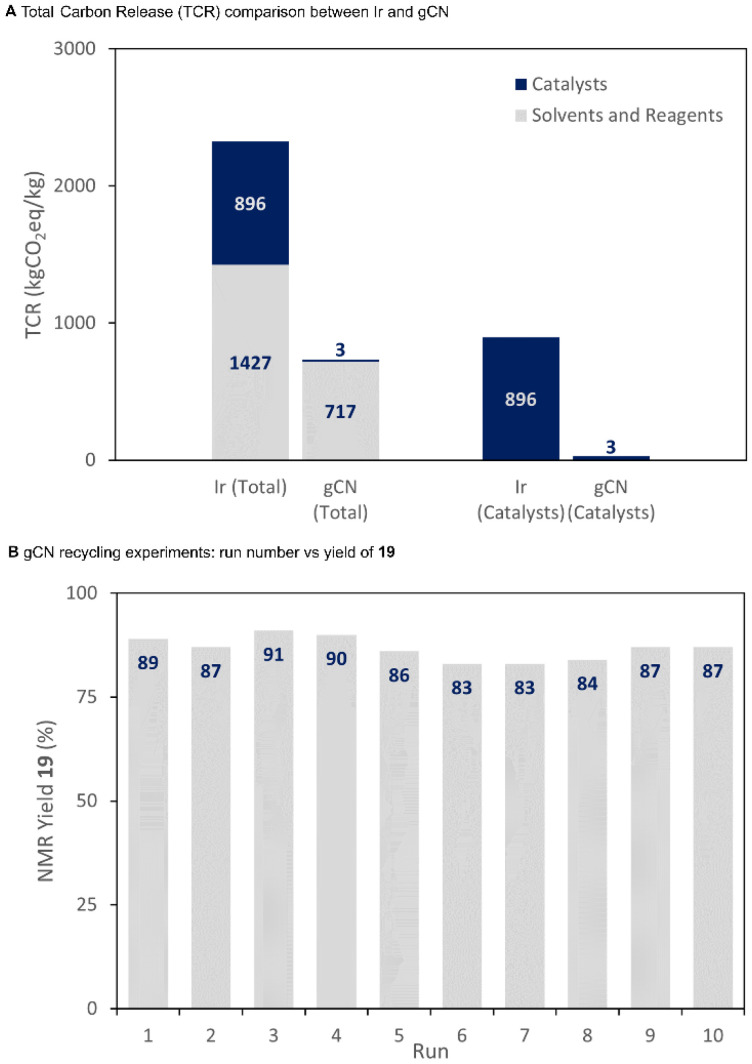
(A) Comparison of TCR for iridium and gCN-catalysed procedures and (B) recycling experiments of gCN.

In addition to its synthetic capabilities, gCN can offer a number of advantages over conventional iridium-based photocatalysts due to its low price, robust nature, and significantly reduced environmental impact. To demonstrate this, we used a recently reported method^38,39^ to calculate the total carbon release (TCR) for our procedure and for an analogous procedure performed with iridium.^29^ By analysis of the results, it can be seen that a significant drop in the environmental impact is observed for the gCN-catalysed procedure compared to the iridium-catalysed process (Scheme 3A). By isolating the contribution of the catalysts from the total of all reaction components, it becomes clear that the majority of the TCR reduction originates from the reduced contribution of the catalytic system, with gCN representing a reduction in TCR by a factor of 300. It should be noted that these TCR calculations do not account for any potential recycling of the photocatalysts. The heterogeneous nature of the gCN photocatalyst enables it to be easily separated from the reaction mixture by filtration or centrifugation, and as a result, the separated photocatalyst can be reused after simple washing and drying steps. To demonstrate the utility, we separated and recycled our gCN photocatalyst for 10 consecutive reaction runs without observing any degradation of yield (Scheme 3B), demonstrating both the facile separation and the robust nature of this photocatalyst.

## Conclusions

In conclusion, we have successfully developed a dual-catalytic cross-coupling procedure with graphitic carbon nitride and nickel catalysis for the synthesis of ketones from α-keto acids and aryl halides. In this system, a broad range of aryl and alkyl keto acids are tolerated, and we have established suitable conditions to prevent Norrish type 2 decomposition of the products containing a γ-hydrogen substituent. We also demonstrate the use of oxamic acids for the modular preparation of amides. By avoiding the use of a precious metal iridium photocatalyst, we achieved a stark reduction in the total carbon release (TCR) of the methodology when compared with previous methods, and demonstrate that the same catalyst can be filtered and reused at least 10 times without loss of reactivity. Further studies on using heterogeneous semiconductors as photocatalysts are ongoing in our laboratory.

## Author contributions

MTF and FL designed the project, with input from TN and BM. MTF, FL, FR, and JL performed and analysed the synthetic experiments. Mechanistic studies, kinetic experiments and analytical measurements were carried out by MTF and FL. All authors provided input during monthly update meetings. MTF, FL and TN wrote the manuscript with input from all authors.

## Conflicts of interest

There are no conflicts to declare.

## Supplementary Material

GC-027-D5GC03641K-s001

## Data Availability

The data supporting this article have been included as part of the supplementary information (SI). Supplementary information is available. See DOI: https://doi.org/10.1039/d5gc03641k.
