# Supplementary material for: Graphitic carbon nitride/nickel dual catalysis for decarboxylative synthesis of unsymmetrical ketones from keto acids
Source: Green Chem. 2025 Oct 24;27(45):14589–94. doi: 10.1039/d5gc03641k (PMC12571222; doi:10.1039/d5gc03641k)
Supplement: GC-027-D5GC03641K-s001 [file GC-027-D5GC03641K-s001.pdf]

Supplementary Information (SI) for Green Chemistry.
This journal is © The Royal Society of Chemistry 2025

Supporting Information

Graphitic carbon nitride/nickel dual catalysis for decarboxylative synthesis of unsymmetrical ketones from keto acids

Michael T. Findlay,[1,†] *Florian Lukas,*[1,†] *Francesca Rizzo,*[1,2] *Junsong Liu,*[1] *Benjamin Martin,*[3] *Simon Allmendinger,*[3] *Markus Furegati,*[3] *Pablo Gabriel,*[3] *and Timothy Noël* [1,*]

[1]Flow Chemistry Group, Van 't Hoff Institute for Molecular Sciences (HIMS), University of Amsterdam, Science Park 904, 1098 XH Amsterdam, The Netherlands.

[2]PhotoGreen Lab, Department of Chemistry, University of Pavia, Viale Taramelli 12, Pavia, 27100 Italy

[3]Novartis Pharma AG, Fabrikstrasse, 4002 Basel, Switzerland.

[†]These authors contributed equally to this work.

* *Email:* t.noel@uva.nl

Table of Contents

1. General information ... 3

2. Chart of synthesized products ... 4

3. Reaction setups ... 5

   3.1 Vials photoreactor (BIG-UFO reactor) ... 5

   3.2 Scale-up reactor (box reactor) ... 6

4. Optimization ... 7

   4.1 Optimization of photocatalyst loading ... 7

   4.2 Optimization of acid and base loading ... 7

   4.3 Optimization of Nickel catalysis ... 9

   4.4 Optimization of Phthalimide loading ... 11

   4.5 Screening of wavelength ... 11

   4.6 Control Experiments ... 12

5. General procedure 1 (GP1) ... 13

6. Mechanistic investigation ... 14

   6.1 Chemical Quenching ... 14

   6.2 Mechanism of methyl 4-acetylbenzoate formation ... 15

7. Spectroscopy measurements ... 16

   7.1 UV-Vis characterization ... 16

   7.2 Single electron microscopy ... 16

   7.3 ATR-IR Spectra of gCN before and after recycling ... 17

8. Scale-up ... 18

9. Recycling experiments ... 19

10. Calculations for TCR ... 20

11. Starting material synthesis ... 21

12. Characterization data of synthesized compounds ... 23

13. Limitations of the scope ... 34

14. NMR spectra of isolated compounds ... 35

1. General information

All reagents and solvents were used as received without further purification, unless stated otherwise. Reagents and solvents were bought from Sigma Aldrich, TCI, Fluorochem and Fisher Scientific and, if applicable, kept under argon atmosphere. Technical solvents were bought from VWR International and Biosolve, and were used as received. Graphitic Carbon Nitride photocatalyst was ordered from NanoChemazone unless otherwise indicated. Disposable syringes were purchased from Laboratory Glass Specialist. Product isolation was performed manually, using silica (60, F254, Merck™). TLC analysis was performed using Silica on aluminum foil TLC plates (F254, Supelco Sigma-Aldrich™) with visualization under ultraviolet light (254 nm and 365 nm) or appropriate TLC staining (cerium ammonium molybdate or potassium permanganate). ^{1}H, ^{13}C, and ^{19}F NMR spectra were recorded, unless stated otherwise, at ambient temperature using a Bruker AV400 or a Bruker AV300. ^{1}H NMR spectra are reported to 2 d.p. in parts per million (ppm) downfield relative to $CDCl_3$ (7.26 ppm) and all ^{13}C NMR spectra are reported to 1 d.p. in ppm relative to $CDCl_3$ (77.2 ppm) unless stated otherwise. The following abbreviations have been adopted to describe the multiplicity: bs (broad singlet), s (singlet), d (doublet), t (triplet), q (quartet), p (pentet), h (hextet), hept (heptet), m (multiplet), dd (double of doublets), td (triple of doublets), tt (triplet of triplets). Coupling constants (*J*) are reported in hertz (Hz) to 1 d.p. NMR data were processed using the MestReNova 14.1.0 software package. Known products were characterized through comparison with the corresponding ^{1}H NMR and ^{13}C NMR from literature. High resolution mass spectra (HRMS) were collected on an AccuTOF LC, JMS-T100LP Mass spectrometer (JEOL, Japen). UV-Vis spectra were recorded with a single-beam Duetta ExSpec equipped with a Xe arc lamp (250-1000 nm) and a CCD camera as a detector. IR spectra were recorded with a Thermo Scientific Nicolet iS50 FT-IR spectrometer. GCMS data was gathered using an Agilent 5977C GC/MSD System. The names of all products were generated using the PerkinElmer ChemBioDraw Ultra v.12.0.2 software package.

For the photochemical batch experiments, a 3D-printed (PLA) reactor internally coated with aluminum foil and equipped with a specific 3D-printed (PLA) lid serving as vials holder and lamp holder was used (see section 3 for details).

2. Chart of synthesized products

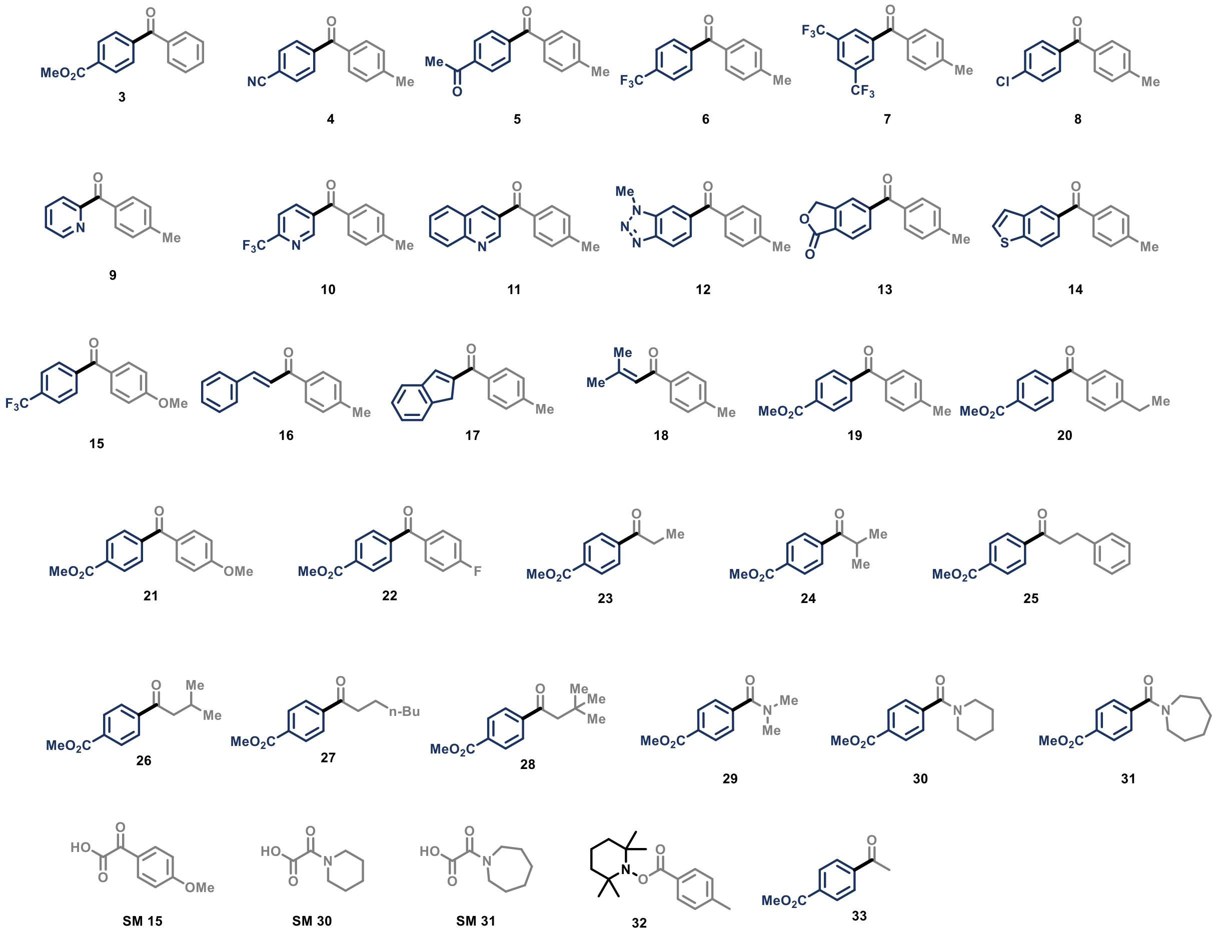

Figure S1. Chart of synthesized products

3. Reaction setups

3.1 Vials photoreactor (BIG-UFO reactor)

Four reactions could be irradiated simultaneously using the adapted "UFO" photoreactor depicted below. A 40W Kessil PR160L-390 nm was used as light source, while the temperature was maintained around 43 °C *via* use of a fan positioned under the reactor.[1]

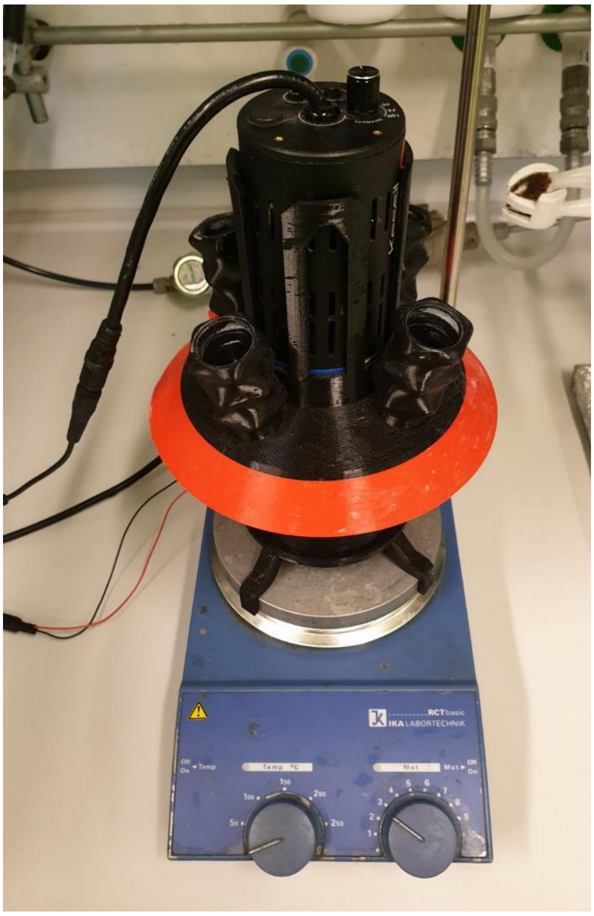

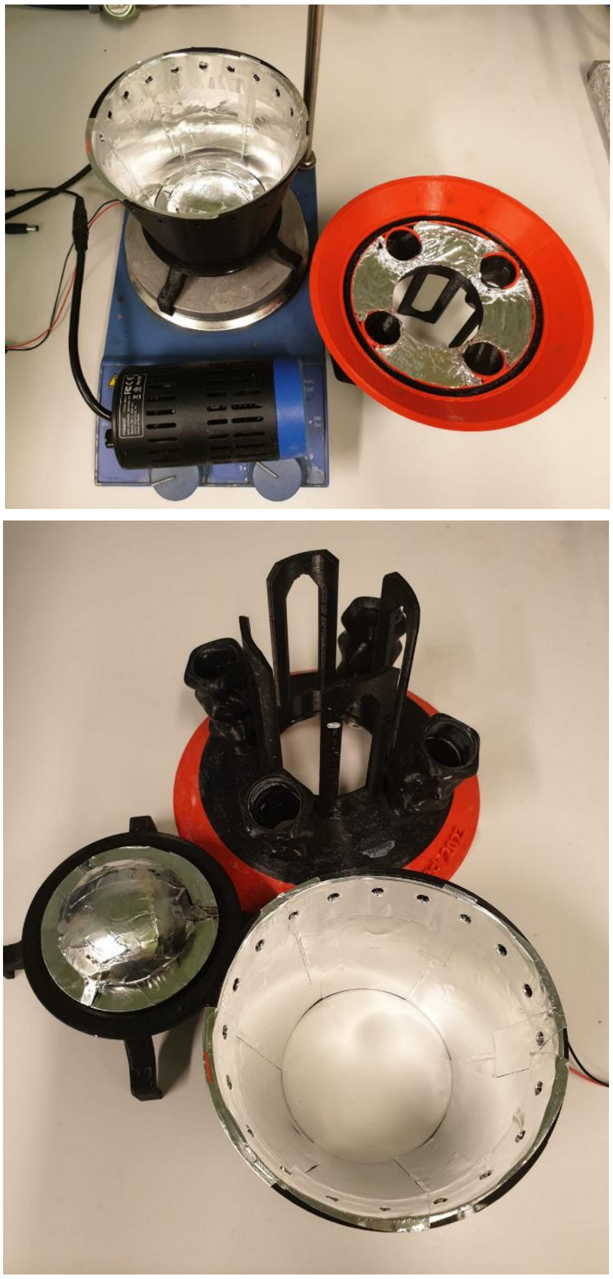

Figure S2. Photoreactor set-up used for optimization and scope

3.2 Scale-up reactor (box reactor)

The reaction was irradiated using the photoreactor depicted below. Two 40W Kessil PR160L- 390nm were used as lamps at a distance of 6 cm, while the temperature was maintained around 40 °C *via* use of a fan positioned on the back side of the reactor.

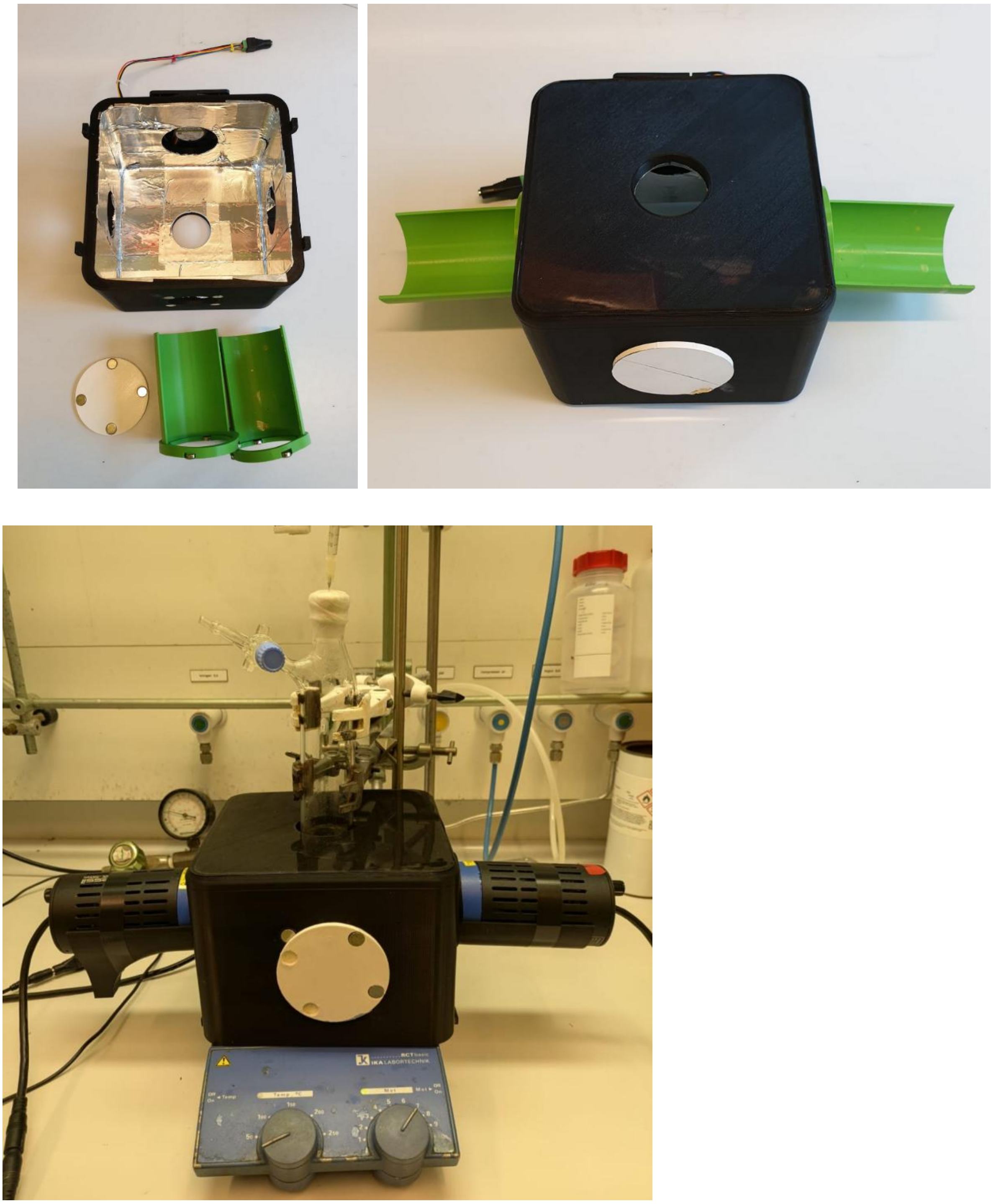

Figure S3. Photoreactor set-up used for reaction scale-up.

4. Optimization

4.1 Optimization of photocatalyst loading

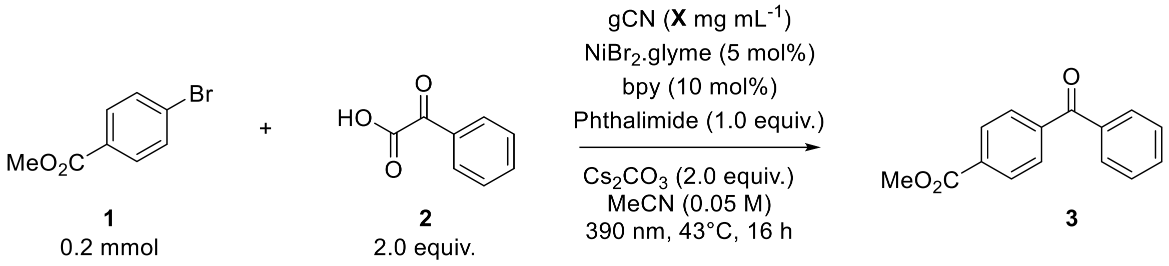

Table S1. Screening of gCN loading.

Entry	X (mg mL^{-1})	Yield of 3 (%)
**1**	5.0	71
**2**	2.5	73
**3**	1.25	50

4.2 Optimization of acid and base loading

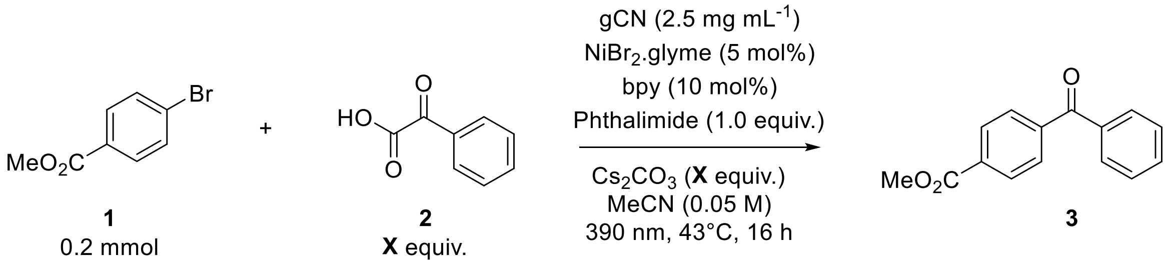

Table S2. Screening of acid loading.

Entry	X (equiv.)	Yield of 3 (%)
**1**	1	35
**2**	2	70
**3**	3	71
**4**	4	74

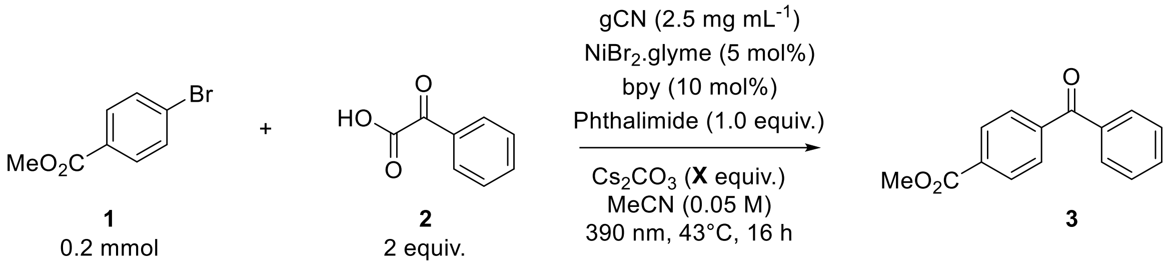

Table S3. Screening of base loading.

Entry	X (equiv.)	Yield of 3 (%)
**1**	1.0	80
**2**	1.5	77
**3**	2.0	81

4.3 Optimization of Nickel catalysis

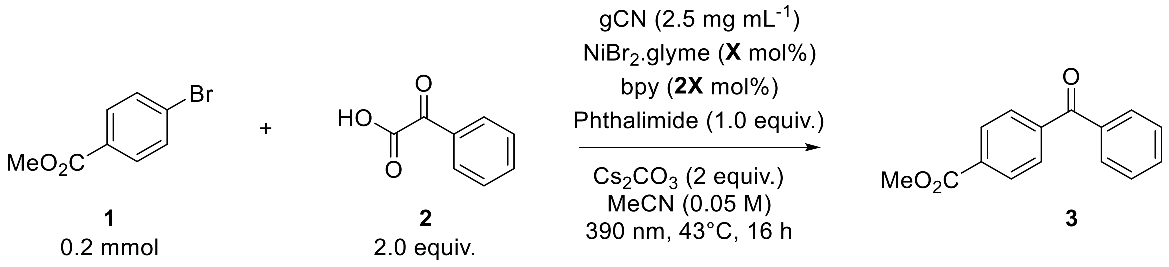

Table S4. Screening of nickel catalyst and ligand loading.

Entry	$NiBr_2$·glyme loading (mol%)	Yield of 3 (%)
1	2.5	42
2	5.0	73
3	7.5	70
4	10.0	63

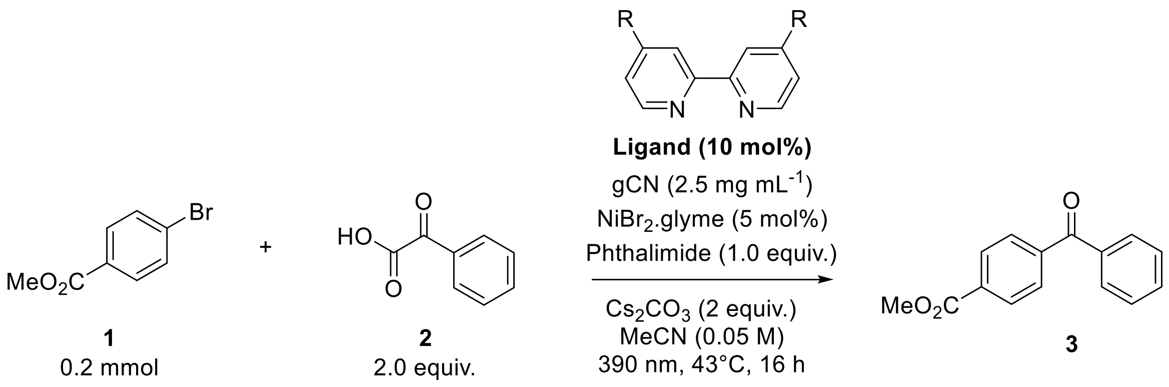

Table S5. Screening of differently substituted bipyridyl ligands.

Entry	Ligand	Yield of 3 (%)
1	bpy (R=H)	70
2	dOMebpy (R=OMe)	28
3	dtbbpy (R=tBu)	43
4	dphenbpy (R=Ph)	47

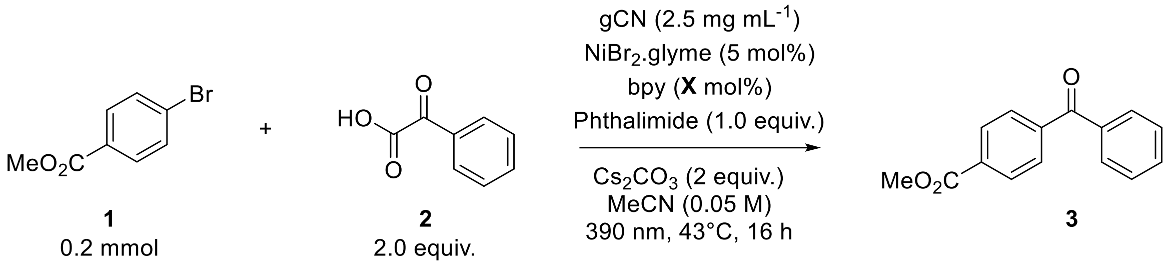

Table S6. Screening of nickel catalyst and ligand ratio.

Entry	X	Yield of 3 (%)
**1**	5	66
**2**	7.5	81
**3**	10	62

4.4 Optimization of Phthalimide loading

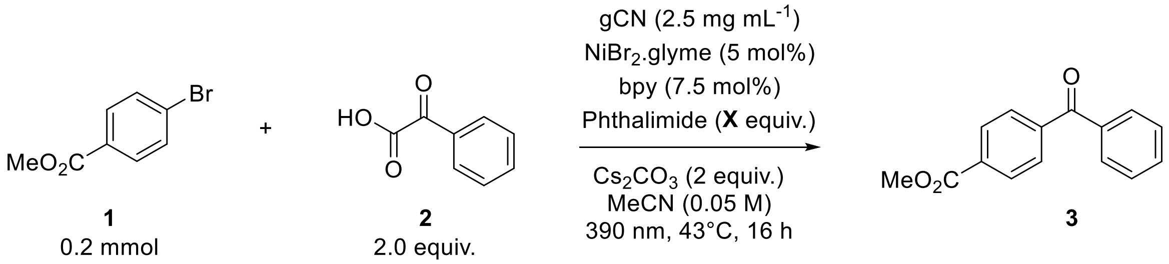

Table S7. Screen of phthalimide loading.

Entry	X (equiv.)	Yield of 3 (%)
**1**	0	25
**2**	0.5	73
**3**	1.0	83
**4**	1.5	81

4.5 Screening of wavelength

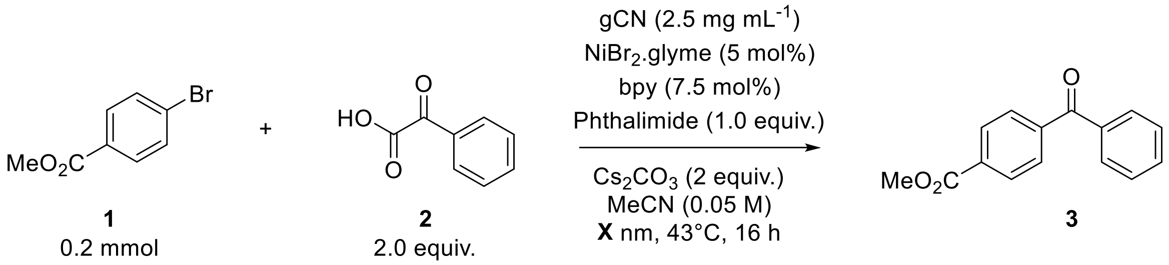

Table S8. Screen of wavelength.

Entry	X (nm)	Yield of 3 (%)	1 recovered (%)
**1**	370	67	0
**2**	390	83	0
**3**	456	45	52

4.6 Control Experiments

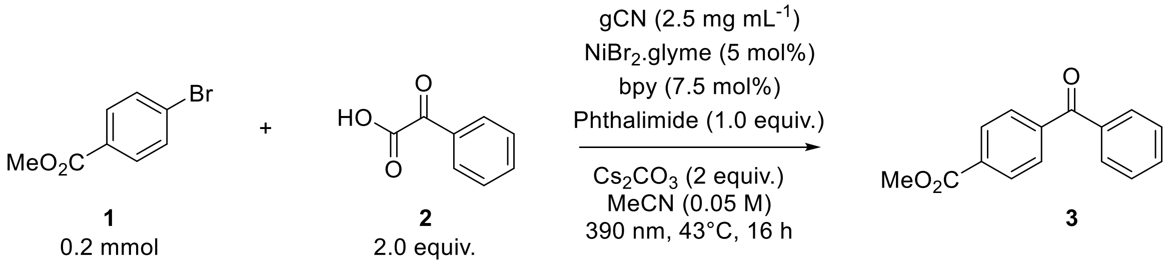

Table S9. Control experiments.

Entry	Conditions	Yield of 3 [%]
**1**	As above	81 (75)
**2**	DMF/DMSO/EtOH*/*i*PrOH* as solvent	16/75/traces/traces
**3**	K_2HPO_3/K_2CO_3/DBU as base	61/72/47
**4**	1, 2 and Cs^{2}CO^{3} (1:1:1) stoichiometry	53
**5**	preformed Ni(dtbbpy)Br_2	75
**6**	TiO_2 instead of gCN, 370 nm / 390 nm	61/84
**7**	Ar-I, Ar-Cl	81/0
**8**	No photocatalyst	0
**9**	No nickel	0
**10**	No light	0

*Reactions performed using EtOH and *i*PrOH as solvent only led to traces of desired product. In both cases, the reactions contained a significant amount of etherification product, a known reaction with this type of system using alcohol substrates, in addition to other minor side products.

5. General procedure 1 (GP1)

In a typical experiment, an oven-dried 30 mL vial equipped with a stirring bar was charged with the aryl bromide (0.2 mmol, 1.0 equiv), phthalimide (0.2 mmol, 1.0 equiv), gCN (from Nanochemazone, unless otherwise specified) (10 mg), the respective α-keto acid (0.4 mmol, 2.0 equiv), and Cs_2CO_3 (0.4 mmol, 2.0 equiv, matching the acid) and the vial was sealed with a rubber septum. Subsequently, the atmosphere was exchanged to N_2 several times and 3 mL degassed, dry acetonitrile were added. $NiBr_2$.glyme (10 µmol, 5 mol %) and 2.2'-bipyridine (15 µmol, 7.5 mol %) were dissolved in 1 mL of degassed, dry acetonitrile and added to the vial to achieve a final concentration of 0.05 M. The vial was stirred and irradiated in the UFO photochemical reactor (see section 3) for the indicated time. The temperature was maintained at approximately 43 °C during the course of the reaction. Then, the vial was removed from the photochemical reactor, the reaction mixture diluted with EtOAc and transferred to a centrifuge vial and centrifuged for 4 min at 4000 rpm. The supernatant was removed and the sediment washed with EtOAc and centrifuged again. The solvent of the combined supernatants was removed under reduced pressure and the crude reaction mixture analysed by qNMR or purified by flash column chromatography on silica gel.

6. Mechanistic investigation

6.1 Chemical Quenching

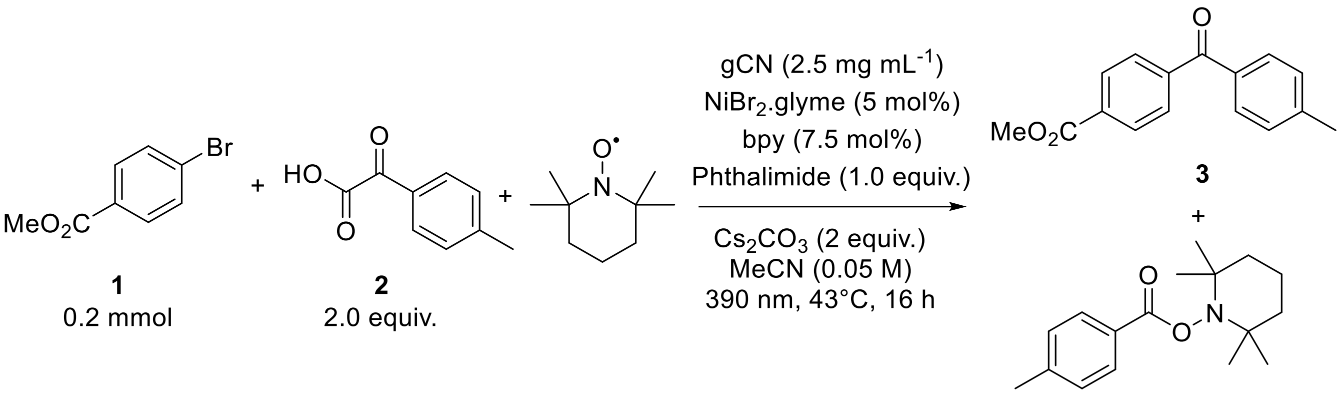

Table S10: Chemical quenching with TEMPO

Entry	**TEMPO** (equiv.)	**Yield 3 (%)**	**Yield 32 (%)**
**1**	0.5	82	42
**2**	1	7	61
**3**	2	0	120
**4**	3	0	150

2,2,6,6-tetramethylpiperidin-1-yl 4-methylbenzoate (32)

^{1}H NMR (300 MHz, $CDCl_3$) δ 7.99 (d, J = 8.3 Hz, 2H), 7.27 (d, J = 6.6 Hz, 2H), 2.43 (s, 3H), 1.87 – 1.70 (m, 3H), 1.65 – 1.55 (m, 2H), 1.53 – 1.41 (m, 1H), 1.28 (s, 6H), 1.13 (s, 6H).

^{13}C NMR (101 MHz, $CDCl_3$) δ 166.6, 143.6, 129.7, 129.3, 127.1, 60.5, 39.2, 32.1, 21.8 (d, J = 4.4 Hz), 21.0, 17.1

HRMS Mass calculated for $C_{17}H_{25}NO_2$ $[M]^+$: 275.1885, mass found: 275.1877.

6.2 Mechanism of methyl 4-acetylbenzoate formation

When 4-methyl-2-oxopentanoic acid and 2-oxooctanoic acid where subjected to conditions as described in GP1 the formation of methyl 4-acetylbenzoate (**13**) was observed in addition to low amounts of the desired products **10** and **12** respectively. Considering that the reaction proceeds only to 10% when set with 2-oxopropanoic acid a decomposition of the formed products seemed likely. A recent study by the group Jie Wu describes the cleavage of alkyl-aryl ketones via 1,5 HAT.[2] Following the conditions used in this paper we wondered if the cleavage could be surpressed by shorter irradiation times or the use of longer wavelength light. The results can be found in Table S11 and S12.

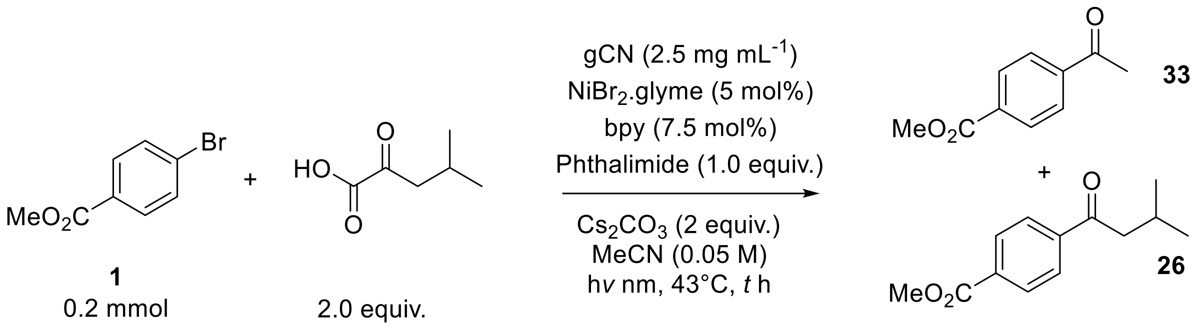

Table S11: methyl 4-acetylbenzoate formation from 4-methyl-2-oxopentanoic acid

Wavelength	Time (h)	Yield 26 (%)	SM 1 remaining (%)	Yield 33 (%)
390	6	12	0	50
390	6	37	0	30
456	16	45	20	0

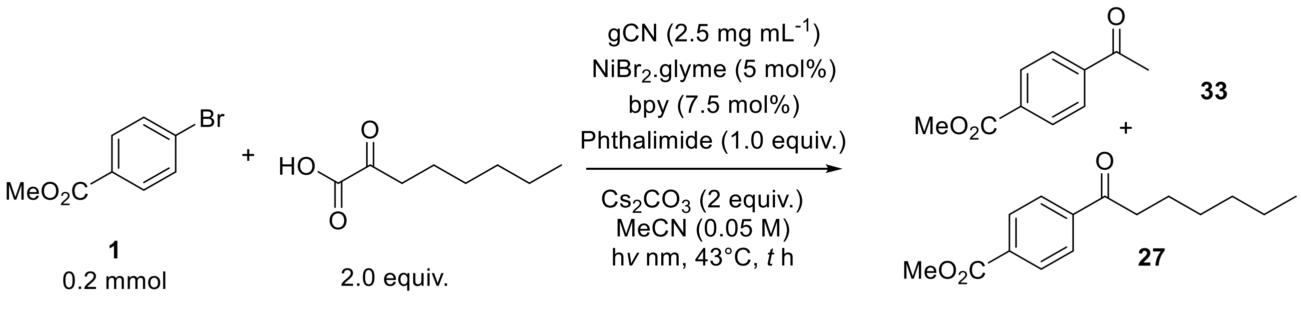

Table S12: methyl 4-acetylbenzoate formation from 2-oxooctanoic acid

Wavelength	Time (h)	Yield 27 (%)	SM 1 remaining (%)	Yield 33 (%)
390	6	10	0	60
390	6	28	20	36
456	16	55	15	0

7. Spectroscopy measurements

7.1 UV-Vis characterization

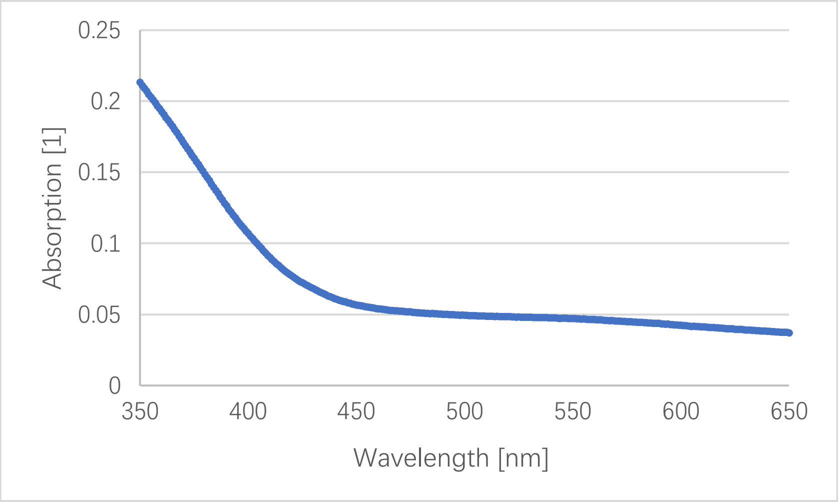

Figure S4: Absorption spectrum of **Ni(bpy)Br$_2$** complex. The spectrum was recorded in MeCN in a quartz cuvette (optical path: 1 cm) with a bandwidth of 5 nm and a data pitch of 1 nm. Scan rate: medium.

7.2 Single electron microscopy

Mag: 50x Mag: 1000x Mag: 50,000x

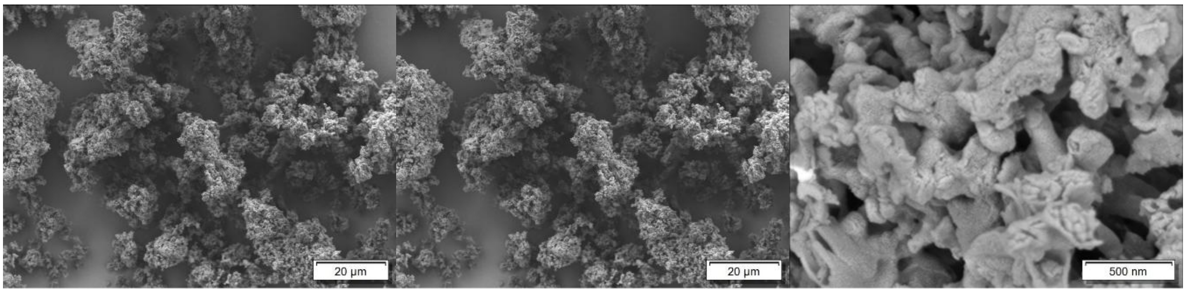

Figure S5: SEM images gCN **Nanochemazone**

→ Specific surface area: **BET analysis** = 46.4 m^2/g; **BJH analysis** = 39 m^2/g

→ **Mesoporous volume/total pore volume**: 0.38 cm^3/g

7.3 ATR-IR Spectra of gCN before and after recycling

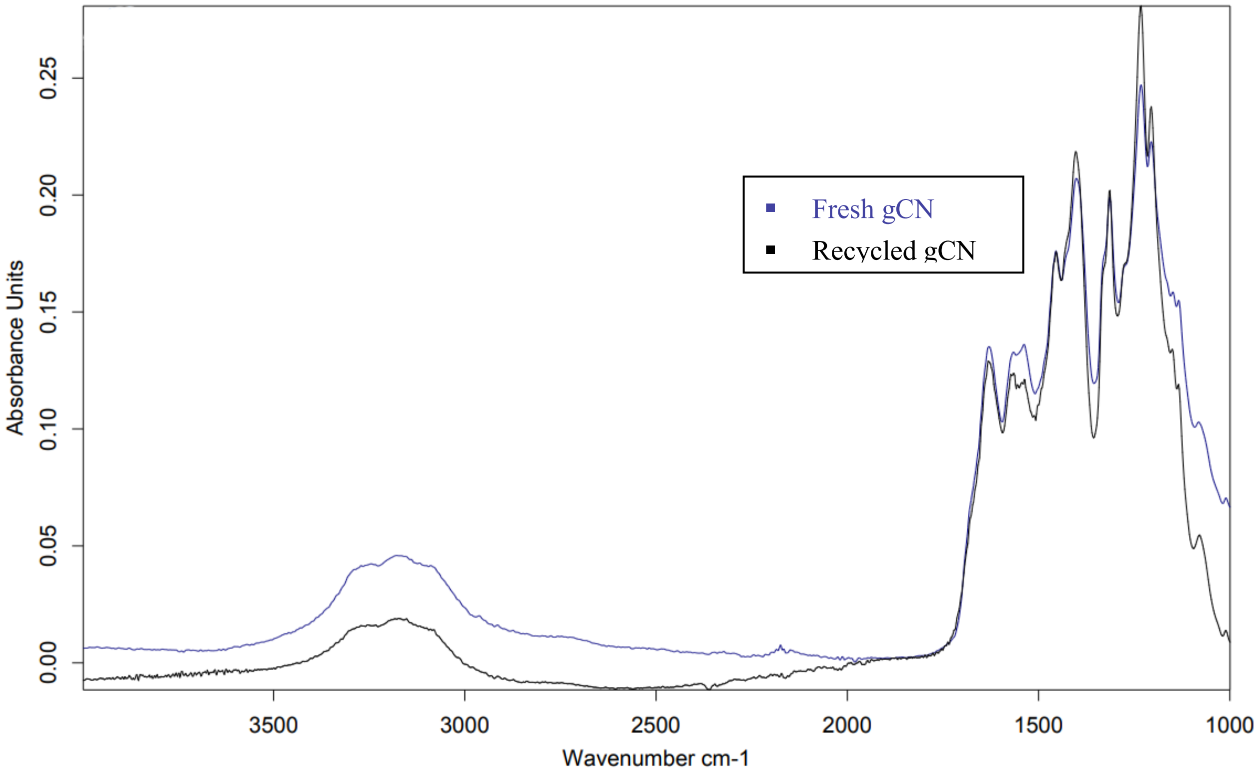

Figure S6: ATR-IR spectra of gCN before and after being recycled.

8. Scale-up

For the scale-up of product **3** some exploratory experiments were run (table S13). For the 5.0 mmol scale experiments the box reactor was used (see section 3). Yields were determined by ^{1}H NMR using TCE as external standard.

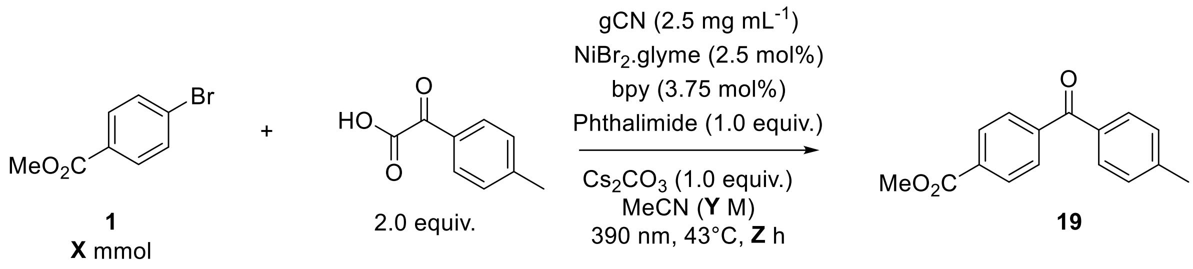

Table S13: Reaction conditions scale up

Entry	Time (h)	Concentration (M)	Scale (mmol)	Setup	Yield of 19 (%)
**1**	16	0.2	2.0	UFO	63
**2**	16	0.1	2.0	UFO	79
**3**	30	0.1	5.0	Box	58
**4**	54	0.1	5.0	Box	72
**5**	104	0.1	5.0	Box	81

Scale-up synthesis of product 3 (5.0 mmol)

An oven dried 500 mL Schlenk flask equipped with a magnetic stirring bar, was charged with methyl 4-bromobenzoate (1.08 g, 5.0 mmol, 1.0 equiv), phthalimide (0.736 g, 5.0 mmol, 1.0 equiv), gCN (250 mg), 2-oxo-2-(p-tolyl)acetic acid (1.64 g, 10.0 mmol, 2.0 equiv), and Cs_2CO_3 (1.63 g, 5.0 mmol, 1.0 equiv) and the Schlenk flask was sealed with a rubber septum. Subsequently, the atmosphere was exchanged to N_2 several times and 50 mL degassed, dry acetonitrile were added. $NiBr_2$.glyme (38.6 mg, 125 µmol, 2.5 mol %) and 2.2'-bipyridine (29 mg, 188 µ mol, 3.75 mol %) were dissolved in 25 mL of degassed, dry acetonitrile and added to the Schlenk flash to achieve the final concentration. The vial was stirred and irradiated in the box reactor (see section 3) for the indicated time. The temperature was maintained at 40 °C during the course of the reaction. The flask was removed from the photochemical reactor, the reaction mixture diluted with EtOAc and transferred in portions to centrifuge vials and centrifuged for 4 min at 4000 rpm. The supernatant was removed and the sediment washed with EtOAc and centrifuged again. The solvent of the combined supernatants was removed under reduced pressure and the crude reaction mixture analysed by ^{1}H NMR using TCE as external standard.

9. Recycling experiments

To demonstrate the recyclability of the gCN catalyst, the model reaction was run as described in GP1 with methyl 4-bromobenzoate (43 mg, 0.2 mmol, 1.0 equiv), 2-oxo-2-(p-tolyl)acetic acid (66 mg, 0.4 mmol, 2.0 equiv) with phthalimide (29 mg, 0.2 mmol, 1.0 equiv), Cs_2CO_3 (130 mg, 0.4 mmol, 2.0 equiv), gCN (10 mg), $NiBr_2$.glyme (3.1 mg, 10 µmol, 5 mol %) and 2.2'-bipyridine (2.3 mg, 15 µmol, 7.5 mol %) for 16 h. The yield of the reaction was determined using ^{1}H NMR with TCE as external standard.

The solids recovered from centrifugation were washed twice with both EtOAc and water. After each washing step the solids were centrifuged and the supernatant discarded. To ensure reproducibility additional washing steps with 1 M HCl twice and then water and acetone once each were implemented.

Recovered gCN catalyst was shown to give the same yield when subjected to the reaction conditions. Exclusion experiments in earlier works had shown a yield of 20% after recycling without further addition of nickel catalyst, after being washed with only EtOAc and water. As such, the additional washing steps were implemented, after which no reactivity was observed without addition of nickel catalyst.

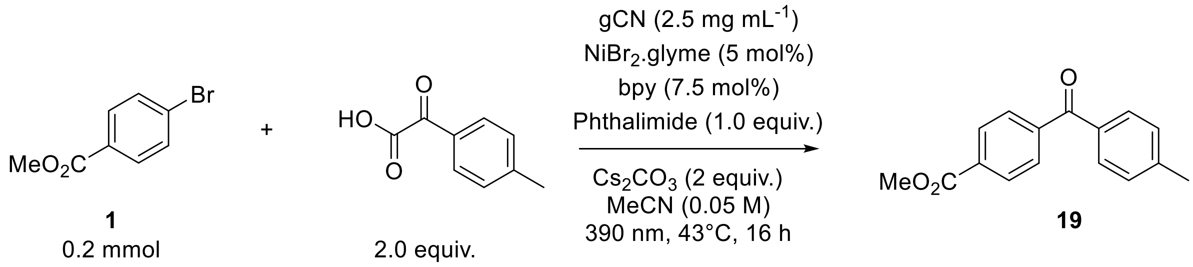

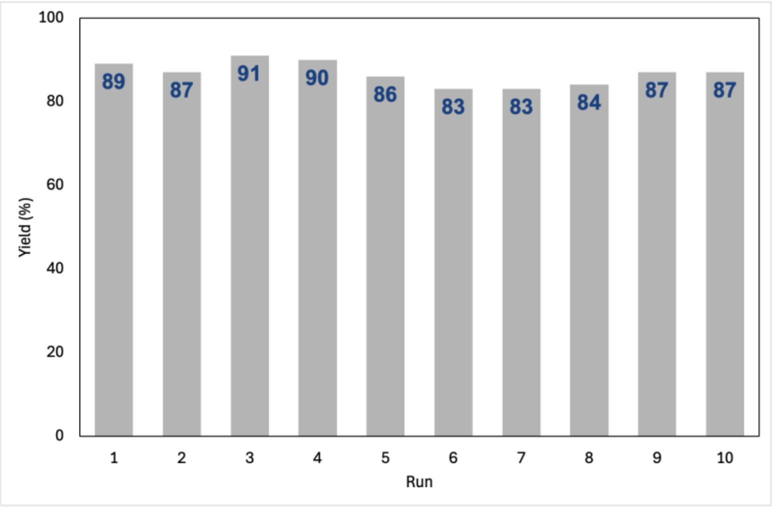

Run	Yield of 19 (%)
1	89
2	87
3	91
4	90
5	86
6	83
7	83
8	84
9	87
10	87

Figure S7: Yield recycling experiments.

10. Calculations for TCR

The TCR calculations were made according to the methodology described in the referenced publications, for the transformations below.[3],[4]

This work:

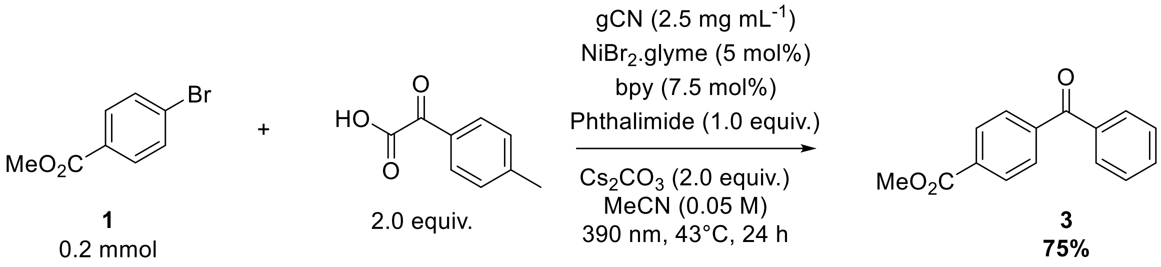

MacMillan group, ACIE, 2015[5]

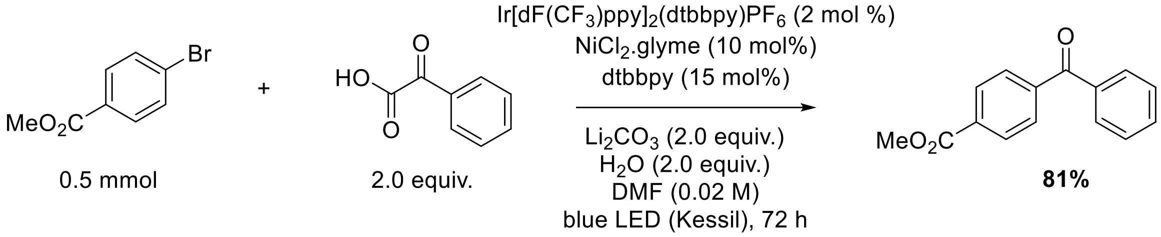

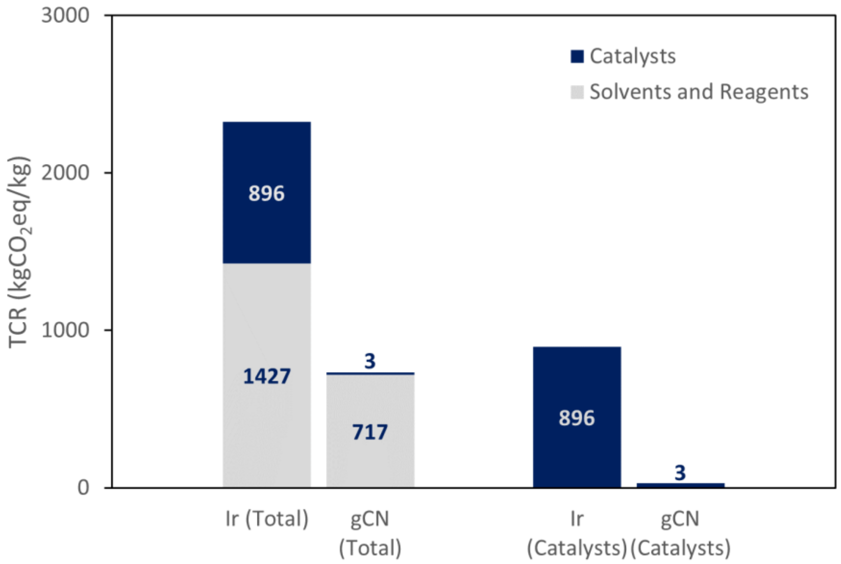

Figure S8: TCR

11. Starting material synthesis

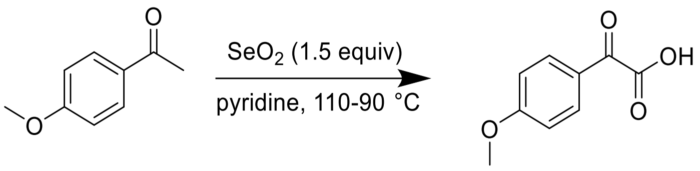

SM 15

The procedure was adapted from literature.[6] To an oven-dried 100-mL round-bottomed flask with a magnetic stir bar, were successively added 1-(4-methoxyphenyl)ethan-1-one (826 mg, 5.5 mmol, 1.0 equiv.), selenium dioxide (916 mg, 8.25 mmol, 1.5 equiv.) and pyridine (5.5 mL, 1.0 M). The reaction mixture was heated in an oil bath to 110 °C for one hour, then the temperature was lowered to 90 °C for an additional four hours. The solution was filtered, and the residue was washed with EtOAc. The combined filtrate was treated successively with 2 N HCl and with water and the organic layer was separated. (A single washing step with 1 N HCl, as described in literature, proved ineffective in removing all the pyridine.) The aqueous fractions were extracted with DCM. The organic layers were combined and dried over sodium sulfate, and the solvent was removed under reduced pressure. While the product appeared clean on NMR, the reactivity was sluggish (20% NMR). After filtration through a silica pad the colour lightened and the reactivity was vastly improved (95% NMR). The product was obtained as a light brown solid (664 mg, 67% yield).

2-(4-methoxyphenyl)-2-oxoacetic acid (SM 15)

Characterization data in accordance with literature.[6]

^{1}H NMR (400 MHz, $CDCl_3$) δ 8.49 (d, J = 9.1 Hz, 2H), 6.99 (d, J = 9.1 Hz, 2H), 3.92 (s, 3H).

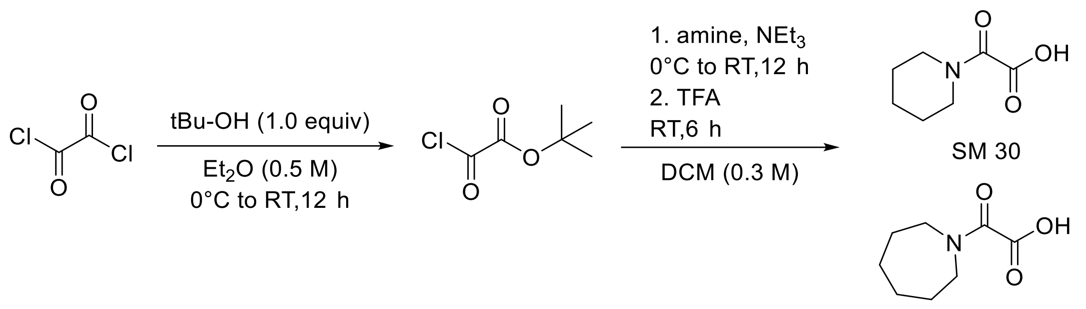

The reaction was performed as described in literature.[7] In an oven-dried Schlenk tube flushed with nitrogen, tert-butanol (10 mmol, 1.0 equiv.) was added to a dry solution of oxalyl chloride (10 mmol, 1.0 equiv.) in diethyl ether (0.5 M) while vigorously stirred at 0°C. Following the complete addition of the tert-butanol, the reaction was allowed to reach room temperature. After 12 hours of stirring, the reaction was stopped and the solvent removed under reduced pressure. The resulting colorless liquid was stored in the refrigerator before use.
Respectively piperidine or azepane (10 mmol, 1.0 equiv.) and triethylamine (12 mmol, 1.2 equiv.) were introduced to a flask containing dry DCM (0.3 M) under nitrogen atmosphere. The solution was cooled to 0°C using an ice bath. Tert-butyl 2-chloro-2-oxoacetate (12 mmol, 1.2 equiv.) obtained from the first step of the reaction was added dropwise over 15 min. The solution was allowed to warm to room temperature and stirred for 6 hours. The reaction mixture was washed with 1.0 M HCl. The aqueous layer extracted with DCM. The combined organic layer was washed with brine, dried over sodium sulfate, and concentrated under reduced pressure. The obtained oil was then redissolved in DCM (0.3M), TFA (5.0 equiv.) was added, and the solution was stirred at room temperature for 12 hours. Solvents were removed at reduced pressure to deliver the desired products as white solids.

2-oxo-2-(piperidin-1-yl)acetic acid (SM 30)

Characterization data are in accordance with literature.[7]

^{1}H NMR (300 MHz, $CDCl_3$) δ 4.01 (t, J = 4.8 Hz, 1H), 3.64 (t, J = 5.1 Hz, 1H), 1.79 – 1.61 (m, 4H).

2-(azepan-1-yl)-2-oxoacetic acid (SM 31)

Characterization data are in accordance with literature.[8]

^{1}H NMR (300 MHz, $CDCl_3$) δ 9.73 (s, 1H), 3.61 (d, J = 5.9 Hz, 2H), 3.55 (d, J = 5.9 Hz, 2H), 1.88 – 1.67 (m, 4H), 1.64 – 1.53 (m, 4H).

12. Characterization data of synthesized compounds

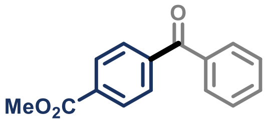

3

methyl 4-benzoylbenzoate (3). Prepared according to GP1 from methyl 4-bromobenzoate (43 mg, 0.2 mmol, 1.0 equiv) and 2-oxo-2-phenylacetic acid (60 mg, 0.4 mmol, 2.0 equiv) with phthalimide (29 mg, 0.2 mmol, 1.0 equiv), Cs_2CO_3 (130 mg, 0.4 mmol, 2.0 equiv), gCN (10 mg), $NiBr_2$.glyme (3.1 mg, 10 µmol, 5 mol %) and 2.2'-bipyridine (2.3 mg, 15 µmol, 7.5 mol %) for 16 h. Purified via flash column chromatography on silica gel (pentane:DCM 25:1) to afford the product as a white solid (36 mg, 75% yield).

Characterization data in accordance with literature.[9]

^{1}H NMR (400 MHz, $CDCl_3$) δ 8.14 (d, J = 8.3 Hz, 2H), 7.83 (d, J = 8.3 Hz, 2H), 7.80 (dd, J = 8.2, 1.4 Hz, 2H), 7.63 – 7.57 (m, 1H), 7.53 – 7.46 (m, 2H), 3.96 (s, 3H).

^{13}C NMR (101 MHz, $CDCl_3$) δ 196.1, 166.4, 141.5, 137.1, 133.4, 133.1, 130.2, 129.9, 129.6, 128.6, 52.6.

GCMS (m/z): [M]+ calcd. for $C_{15}H_{12}O_3$: 240.08, mass found: 240.00.

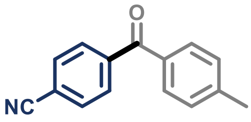

4

4-(4-methylbenzoyl)benzonitrile (4). Prepared according to GP1 from 4-bromobenzonitrile (36 mg, 0.2 mmol, 1.0 equiv) and 2-oxo-2-(p-tolyl)acetic acid (66 mg, 0.4 mmol, 2.0 equiv) with phthalimide (29 mg, 0.2 mmol, 1.0 equiv), Cs_2CO_3 (130 mg, 0.4 mmol, 2.0 equiv), gCN (10 mg), $NiBr_2$.glyme (3.1 mg, 10 µmol, 5 mol %) and 2.2'-bipyridine (2.3 mg, 15 µmol, 7.5 mol %) for 16 h. Purified via flash column chromatography on silica gel (pentane:EtOAc 15:1) to afford the product as a white solid (25 mg, 55% yield).

Characterization data in accordance with literature.[10]

^{1}H NMR (300 MHz, $CDCl_3$) δ 7.85 (d, J = 8.6 Hz, 2H), 7.78 (d, J = 8.6 Hz, 2H), 7.69 (d, J = 8.2 Hz, 2H), 7.31 (d, J = 7.9 Hz, 2H), 2.46 (s, 3H).

^{13}C NMR (75 MHz, $CDCl_3$) δ 194.9, 144.5, 141.8, 133.8, 132.3, 130.4, 130.3, 129.5, 118.2, 115.6, 21.9.

GCMS (m/z): [M]+ calcd. for $C_{15}H_{11}NO$: 221.08, mass found: 221.00.

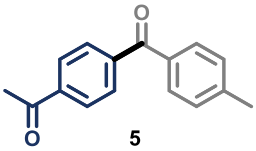

5

1-(4-(4-methylbenzoyl)phenyl)ethan-1-one (5). Prepared according to GP1 from 1-(4-bromophenyl)ethan-1-one (36 mg, 0.2 mmol, 1.0 equiv) and 2-oxo-2-(p-tolyl)acetic acid (66 mg, 0.4 mmol, 2.0 equiv) with phthalimide (29 mg, 0.2 mmol, 1.0 equiv), Cs_2CO_3 (130 mg, 0.4 mmol, 2.0 equiv), gCN (10 mg), $NiBr_2$.glyme (3.1 mg, 10 µmol, 5 mol %) and 2.2'-bipyridine (2.3 mg, 15 µmol, 7.5 mol %) for 16 h. Purified via flash column chromatography on silica gel (pentane:EtOAc 10:1) to afford the product as a yellow solid (32 mg, 68% yield).

Characterization data in accordance with literature.[10]

^{1}H NMR (400 MHz, $CDCl_3$) δ 8.04 (d, J = 8.4 Hz, 2H), 7.83 (d, J = 8.4 Hz, 2H), 7.71 (d, J = 8.2 Hz, 2H), 7.29 (d, J = 7.9 Hz, 2H), 2.66 (s, 3H), 2.45 (s, 3H).

^{13}C NMR (101 MHz, $CDCl_3$) δ 197.7, 195.8, 144.1, 141.9, 139.5, 134.4, 130.5, 130.0, 129.3, 128.2, 27.0, 21.8.

GCMS (m/z): [M]+ calcd. for $C_{16}H_{14}O_2$: 238.10, mass found: 238.00.

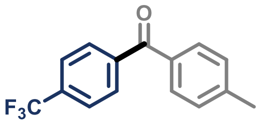

6

***p*-tolyl(4-(trifluoromethyl)phenyl)methanone (6).** Prepared according to GP1 from 1-bromo-4-(trifluoromethyl)benzene (45 mg, 0.2 mmol, 1.0 equiv) and 2-oxo-2-(p-tolyl)acetic acid (66 mg, 0.4 mmol, 2.0 equiv) with phthalimide (29 mg, 0.2 mmol, 1.0 equiv), Cs_2CO_3 (130 mg, 0.4 mmol, 2.0 equiv), gCN (10 mg), $NiBr_2$.glyme (3.1 mg, 10 µmol, 5 mol %) and 2.2'-bipyridine (2.3 mg, 15 µmol, 7.5 mol %) for 16 h. Purified via flash column chromatography on silica gel (pentane:EtOAc 60:1) to afford the product as a white solid (41 mg, 78% yield).

Characterization data in accordance with literature.[11]

^{1}H NMR (400 MHz, $CDCl_3$) δ 7.87 (d, J = 7.9 Hz, 2H), 7.74 (d, J = 9.6 Hz, 2H), 7.72 (d, J = 8.4 Hz, 2H), 7.31 (d, J = 7.9 Hz, 2H), 2.46 (s, 3H).

^{13}C NMR (101 MHz, $CDCl_3$) δ 195.42, 144.24, 141.27, 134.22, 133.65 (q, J = 32.7 Hz), 130.49, 130.15, 129.38, 125.43 (q, J = 3.7 Hz), 123.9 (q, J = 273.0 Hz), 21.86.

^{19}F NMR (282 MHz, $CDCl_3$) δ -62.97.

GCMS (m/z): [M]+ calcd. for $C_{15}H_{11}F_3O$: 264.08, mass found: 264.00.

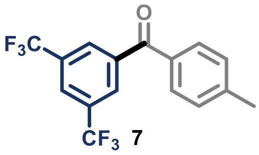

7

(3,5-bis(trifluoromethyl)phenyl)(p-tolyl)methanone methanone (7). Prepared according to GP1 from 1,3-bis(trifluoromethyl)-5-bromobenzene (59 mg, 0.2 mmol, 1.0 equiv) and 2-oxo-2-(p-tolyl)acetic acid (66 mg, 0.4 mmol, 2.0 equiv) with phthalimide (29 mg, 0.2 mmol, 1.0 equiv), Cs_2CO_3 (130 mg, 0.4 mmol, 2.0 equiv), gCN (10 mg), $NiBr_2$.glyme (3.1 mg, 10 µmol, 5 mol %) and 2.2'-bipyridine (2.3 mg, 15 µmol, 7.5 mol %) for 16 h. Purified via flash column chromatography on silica gel (pentane:EtOAc 70:1) to afford the product as a white solid (51 mg, 77% yield).

Characterization data in accordance with literature.[12]

^{1}H NMR (300 MHz, $CDCl_3$) δ 8.22 (s, 2H), 8.08 (s, 1H), 7.70 (d, J = 8.2 Hz, 2H), 7.35 (d, J = 7.9 Hz, 2H), 2.48 (s, 3H).

^{13}C NMR (75 MHz, $CDCl_3$) δ 193.4, 144.9, 139.9, 133.4, 132.1 (q, J = 34.1 Hz), 130.4, 129.9 (q, J = 3.7 Hz), 129.7, 125.8-125.3 (m), 123.1 (q, J = 273.0 Hz), 21.9.

^{19}F NMR (282 MHz, $CDCl_3$) δ -62.89.

GCMS (m/z): [M]+ calcd. for $C_{16}H_{10}F_6O$: 332.06, mass found: 332.10.

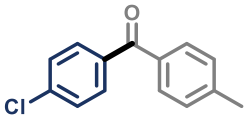

8

(4-chlorophenyl)(p-tolyl)methanone (8). Prepared according to GP1 from 1-bromo-4-chlorobenzene (38 mg, 0.2 mmol, 1.0 equiv) and 2-oxo-2-(p-tolyl)acetic acid (66 mg, 0.4 mmol, 2.0 equiv) with phthalimide (29 mg, 0.2 mmol, 1.0 equiv), Cs_2CO_3 (130 mg, 0.4 mmol, 2.0 equiv), gCN (10 mg), $NiBr_2$.glyme (3.1 mg, 10 µmol, 5 mol %) and 2.2'-bipyridine (2.3 mg, 15 µmol, 7.5 mol %) for 16 h. Purified via flash column chromatography on silica gel (pentane:DCM 5:1) to afford the product as a white solid (34 mg, 74% yield).

Characterization data in accordance with literature.[10]

^{1}H NMR (400 MHz, $CDCl_3$) δ 7.73 (d, J = 8.7 Hz, 2H), 7.69 (d, J = 7.8 Hz, 2H), 7.45 (d, J = 8.4 Hz, 2H), 7.29 (d, J = 7.8 Hz, 2H), 2.45 (s, 3H).

^{13}C NMR (101 MHz, $CDCl_3$) δ 195.4, 143.7, 138.7, 136.4, 134.7, 131.5, 130.3, 129.2, 128.7, 21.8.

GCMS (m/z): [M]+ calcd. for. $C_{14}H_{11}ClO$: 230.05, mass found: 230.10.

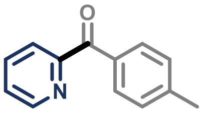

9

pyridin-2-yl(p-tolyl)methanone (9). Prepared according to GP1 from 2-bromopyridine (31 mg, 0.2 mmol, 1.0 equiv) and 2-oxo-2-(p-tolyl)acetic acid (66 mg, 0.4 mmol, 2.0 equiv) with phthalimide (29 mg, 0.2 mmol, 1.0 equiv), Cs_2CO_3 (130 mg, 0.4 mmol, 2.0 equiv), gCN (10 mg), $NiBr_2$.glyme (3.1 mg, 10 µmol, 5 mol %) and 2.2'-bipyridine (2.3 mg, 15 µmol, 7.5 mol %) for 16 h. Purified via flash column chromatography on silica gel (pentane:EtOAc 20:1) to afford the product as a white solid (16 mg, 41% yield).

Characterization data in accordance with literature.[13]

^{1}H NMR (300 MHz, $CDCl_3$) δ 8.72 (d, J = 4.7 Hz, 1H), 8.01 (d, J = 7.7 Hz, 1H), 7.98 (d, J = 8.2 Hz, 2H), 7.89 (app. td, J = 7.7, 1.7 Hz, 1H), 7.52 – 7.42 (m, 1H), 7.29 (d, J = 8.1 Hz, 2H), 2.43 (s, 3H).

^{13}C NMR (101 MHz, $CDCl_3$) δ 193.7, 155.6, 148.7, 143.9, 137.1, 133.8, 131.3, 129.1, 126.1, 124.7, 21.9.

GCMS (m/z): [M]+ calcd. for. $C_{13}H_{11}NO$: 197.08, mass found: 197.10.

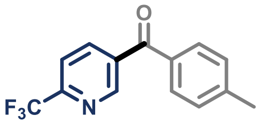

10

p-tolyl(6-(trifluoromethyl)pyridin-3-yl)methanone (10). Prepared according to GP1 from 5-bromo-2-(trifluoromethyl)pyridine (45 mg, 0.2 mmol, 1.0 equiv) and 2-oxo-2-(p-tolyl)acetic acid (66 mg, 0.4 mmol, 2.0 equiv) with phthalimide (29 mg, 0.2 mmol, 1.0 equiv), Cs_2CO_3 (130 mg, 0.4 mmol, 2.0 equiv), gCN (10 mg), $NiBr_2$.glyme (3.1 mg, 10 µmol, 5 mol %) and 2.2'-bipyridine (2.3 mg, 15 µmol, 7.5 mol %) for 16 h. Purified via flash column chromatography on silica gel (pentane:EtOAc 30:1) to afford the product as a white solid (39 mg, 73% yield).

Characterization data in accordance with literature.[12]

^{1}H NMR (400 MHz, $CDCl_3$) δ 9.04 (s, 1H), 8.25 (d, J = 7.7 Hz, 1H), 7.83 (d, J = 8.1 Hz, 1H), 7.73 (d, J = 8.1 Hz, 2H), 7.33 (d, J = 7.9 Hz, 2H), 2.46 (s, 3H).

^{13}C NMR (101 MHz, $CDCl_3$) δ 193.3, 150.7, 150.5 (q, J = 35.1 Hz), 145.1, 138.7, 136.1, 133.5, 130.4, 129.7, 121.3 (q, J = 274.5 Hz), 120.4 (q, J = 2.7 Hz), 21.9.

^{19}F NMR (282 MHz, $CDCl_3$) δ -68.14.

GCMS (m/z): [M]+ calcd. for. $C_{14}H_{10}F_3NO$: 265.07, mass found: 265.10.

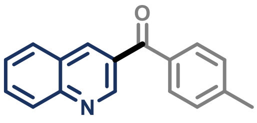

11

quinolin-3-yl(p-tolyl)methanone (11). Prepared according to GP1 from 3-bromoquinoline (42 mg, 0.2 mmol, 1.0 equiv) and 2-oxo-2-(p-tolyl)acetic acid (66 mg, 0.4 mmol, 2.0 equiv) with phthalimide (29 mg, 0.2 mmol, 1.0 equiv), Cs_2CO_3 (130 mg, 0.4 mmol, 2.0 equiv), gCN (10 mg), $NiBr_2$.glyme (3.1 mg, 10 µmol, 5 mol %) and 2.2'-bipyridine (2.3 mg, 15 µmol, 7.5 mol %) for 16 h. Purified via flash column chromatography on silica gel (pentane:EtOAc 3:1) to afford the product as a white solid (17 mg, 34% yield).

Characterization data in accordance with literature.[14]

^{1}H NMR (300 MHz, $CDCl_3$) δ 9.31 (d, *J* = 2.2 Hz, 1H), 8.55 (d, *J* = 1.9 Hz, 1H), 8.20 (d, *J* = 8.4 Hz, 1H), 7.96 – 7.90 (m, 1H), 7.89 – 7.82 (m, 1H), 7.79 (d, *J* = 8.2 Hz, 2H), 7.70 – 7.58 (m, 1H), 7.34 (d, *J* = 7.6 Hz, 2H), 2.48 (s, 3H).

^{13}C NMR (75 MHz, $CDCl_3$) δ 194.7, 150.5, 149.5, 144.2, 138.7, 134.5, 131.9, 130.6, 130.4, 129.6, 129.5, 129.2, 127.7, 126.8, 21.9.

GCMS (m/z): [M]+ calcd. for. $C_{17}H_{13}NO$: 247.10, mass found: 247.10.

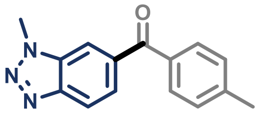

12

(1-methyl-1H-benzo[d][1,2,3]triazol-6-yl)(p-tolyl)methanone (12). Prepared according to GP1 6-bromo-1-methyl-1H-benzo[d][1,2,3]triazole (42 mg, 0.2 mmol, 1.0 equiv) and 2-oxo-2-(p-tolyl)acetic acid (66 mg, 0.4 mmol, 2.0 equiv) with phthalimide (29 mg, 0.2 mmol, 1.0 equiv), Cs_2CO_3 (130 mg, 0.4 mmol, 2.0 equiv), gCN (10 mg), $NiBr_2$.glyme (3.1 mg, 10 µmol, 5 mol %) and 2.2'-bipyridine (2.3 mg, 15 µmol, 7.5 mol %) for 16 h. Purified via flash column chromatography on silica gel (pentane:EtOAc 10:1) to afford the product as a yellow solid (29 mg, 58% yield).

^{1}H NMR (300 MHz, $CDCl_3$) δ 8.12 (d, *J* = 8.6 Hz, 1H), 8.01 – 7.95 (m, 1H), 7.77 (dd, *J* = 8.5, 1.4 Hz, 1H), 7.73 (d, *J* = 8.1 Hz, 2H), 7.31 (d, *J* = 7.8 Hz, 2H), 4.35 (s, 3H), 2.46 (s, 3H).

^{13}C NMR (101 MHz, $CDCl_3$) δ 195.9, 144.0, 137.2, 134.7, 134.4, 130.5, 129.3, 125.4, 123.7, 119.8, 111.9, 34.7, 21.8.

HRMS Mass calculated for $C_{15}H_{13}N_3O$ $[M+H]^+$: 252.1137, mass found: 252.1140.

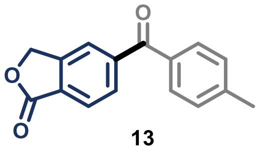

13

5-(4-methylbenzoyl)isobenzofuran-1(3H)-one (13). Prepared according to GP1 from 5-bromoisobenzofuran-1(3H)-one (43 mg, 0.2 mmol, 1.0 equiv) and 2-oxo-2-(p-tolyl)acetic acid (66 mg, 0.4 mmol, 2.0 equiv) with phthalimide (29 mg, 0.2 mmol, 1.0 equiv), Cs_2CO_3 (130 mg, 0.4 mmol, 2.0 equiv), gCN (10 mg), $NiBr_2$.glyme (3.1 mg, 10 µmol, 5 mol %) and 2.2'-bipyridine (2.3 mg, 15 µmol, 7.5 mol %) for 16 h. Purified via flash column chromatography on silica gel (pentane:EtOAc 80:1) to afford the product as a white solid (41 mg, 81% yield).

^{1}H NMR 300 MHz, $CDCl_3$) δ 8.02 (d, *J* = 8.4 Hz, 1H), 7.92 – 7.84 (m, 2H), 7.72 (d, *J* = 8.2 Hz, 2H), 7.32 (d, *J* = 7.9 Hz, 2H), 5.40 (s, 2H), 2.46 (s, 3H).

^{13}C NMR (101 MHz, $CDCl_3$) δ 195.4, 170.3, 146.6, 144.6, 143.5, 134.0, 130.6, 130.5, 129.5, 128.5, 125.8, 123.4, 69.8, 21.9.

HRMS Mass calculated for $C_{16}H_{12}O_3$ $[M]^+$: 252.0786, mass found: 252.0794.

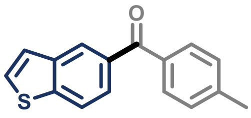

14

benzo[b]thiophen-5-yl(p-tolyl)methanone (14). Prepared according to GP1 from 5-bromoisobenzofuran-1(3H)-one (43 mg, 0.2 mmol, 1.0 equiv) and 2-oxo-2-(p-tolyl)acetic acid (99 mg, 0.6 mmol, 3.0 equiv) with phthalimide (29 mg, 0.2 mmol, 1.0 equiv), Cs_2CO_3 (195 mg, 0.6 mmol, 3.0 equiv), gCN (10 mg), $NiBr_2$.glyme (3.1 mg, 10 µmol, 5 mol %) and 2.2'-bipyridine (2.3 mg, 15 µmol, 7.5 mol %) for 16 h. Purified via flash column chromatography on silica gel (pentane:EtOAc 50:1) to afford the product as a yellow solid (26 mg, 52% yield).

^{1}H NMR (400 MHz, $CDCl_3$) δ 8.24 (d, J = 1.6 Hz, 1H), 7.97 (d, J = 8.4 Hz, 1H), 7.81 (dd, J = 8.4, 1.6 Hz, 1H), 7.75 (d, J = 8.2 Hz, 2H), 7.54 (d, J = 5.4 Hz, 1H), 7.42 (d, J = 5.5 Hz, 1H), 7.30 (d, J = 7.9 Hz, 2H), 2.46 (s, 3H).

^{13}C NMR (101 MHz, $CDCl_3$) δ 196.7, 143.7, 143.2, 139.2, 135.4, 134.4, 130.4, 129.1, 127.9, 126.3, 125.5, 124.7, 122.5, 21.8.

HRMS Mass calculated for $C_{16}H_{12}OS$ $[M]^+$: 252.0609, mass found: 252.0616.

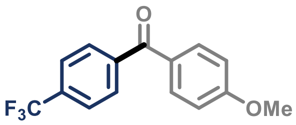

15

(4-methoxyphenyl)(4-(trifluoromethyl)phenyl)methanone (15). Prepared according to GP1 from methyl 1-bromo-4(trifluoromethyl)benzene (45 mg, 0.2 mmol, 1.0 equiv) and 2-(4-methoxyphenyl)-2-oxoacetic acid (72 mg, 0.4 mmol, 2.0 equiv) with phthalimide (29 mg, 0.2 mmol, 1.0 equiv), Cs_2CO_3 (130 mg, 0.4 mmol, 2.0 equiv), gCN (10 mg), $NiBr_2$.glyme (3.1 mg, 10 µmol, 5 mol %) and 2.2'-bipyridine (2.3 mg, 15 µmol, 7.5 mol %) for 16 h. Purified via flash column chromatography on silica gel (pentane:EtOAc 20:1) to afford the product as a white solid (41 mg, 73% yield).

^{1}H NMR ^{1}H NMR (400 MHz, $CDCl_3$) δ 7.88 – 7.79 (m, 4H), 7.74 (d, J = 8.11 Hz, 2H), 6.98 (d, J = 8.81 Hz, 2H), 3.90 (s, 3H).

^{13}C NMR (101 MHz, $CDCl_3$) δ 194.4, 163.9, 141.7, 133.4 (q, J = 32.56 Hz), 132.8, 129.9, 129.5, 125.4 (q, J = 3.83 Hz), 123.9 (q, J = 272.41 Hz), 114.0, 55.7.

^{19}F NMR (282 MHz, $CDCl_3$) δ -62.94.

GCMS (m/z): [M]+ calcd. for $C_{15}H_{11}F_3O_2$: 280.07, mass found: 280.10

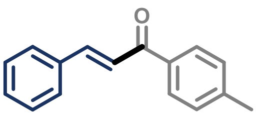

16

3-phenyl-1-(p-tolyl)prop-2-en-1-one (16). Prepared according to GP1 from (E)-(2-bromovinyl)benzene (37 mg, 0.2 mmol, 1.0 equiv) and 2-oxo-2-(p-tolyl)acetic acid (99 mg, 0.6 mmol, 3.0 equiv) with phthalimide (29 mg, 0.2 mmol, 1.0 equiv), Cs2CO3 (195 mg, 0.6 mmol, 3.0 equiv), gCN (10 mg), NiBr2.glyme (3.1 mg, 10 µmol, 5 mol %) and 2.2'-bipyridine (2.3 mg, 15 µmol, 7.5 mol %) for 16 h. Purified via flash column chromatography on silica gel (pentane:EtOAc 100:1) to afford the product as a white solid (16 mg, 36% yield).

Characterization data in accordance with literature.[16]

^{1}H NMR (400 MHz, $CDCl_3$) δ 7.94 (d, J = 8.3 Hz, 2H), 7.81 (d, J = 15.7 Hz, 1H), 7.67 – 7.62 (m, 2H), 7.54 (d, J = 15.7 Hz, 1H), 7.45 – 7.39 (m, 3H), 7.31 (d, J = 8.1 Hz, 2H), 2.44 (s, 3H).

^{13}C NMR (101 MHz, $CDCl_3$) δ 190.2, 144.6, 143.8, 135.8, 135.2, 130.6, 129.5, 129.1, 128.8, 128.6, 122.3, 21.8.

GCMS (m/z): [M]+ calcd. for $C_{16}H_{14}O$: 222.10, mass found: 222.10.

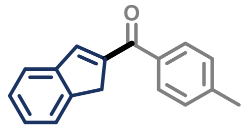

17

(1H-inden-2-yl)(p-tolyl)methanone (17). Prepared according to GP1 from 2-bromo-1H-indene (39 mg, 0.2 mmol, 1.0 equiv) and 2-oxo-2-(p-tolyl)acetic acid (66 mg, 0.4 mmol, 2.0 equiv) with phthalimide (29 mg, 0.2 mmol, 1.0 equiv), Cs2CO3 (130 mg, 0.4 mmol, 2.0 equiv), gCN (10 mg), NiBr2.glyme (3.1 mg, 10 µmol, 5 mol %) and 2.2'-bipyridine (2.3 mg, 15 µmol, 7.5 mol %) for 16 h. Purified via flash column chromatography on silica gel (pentane:EtOAc 50:1) to afford the product as a yellow solid (24 mg, 51% yield).

Characterization data in accordance with literature.[17]

^{1}H NMR (400 MHz, CDCl3) δ 7.76 (d, J = 8.0 Hz, 2H), 7.60 – 7.51 (m, 2H), 7.49 (t, J = 2.1 Hz, 1H), 7.42 – 7.33 (m, 2H), 7.30 (d, J = 7.9 Hz, 2H), 3.89 (s, 2H), 2.45 (s, 3H).

^{13}C NMR (101 MHz, CDCl3) δ 192.8, 145.4, 145.1, 143.3, 143.2, 142.7, 136.4, 129.2, 129.2, 128.2, 127.1, 124.6, 124.0, 38.8, 21.7.

GCMS (m/z): [M]+ calcd. for $C_{17}H_{14}O$: 234.10, mass found: 234.10.

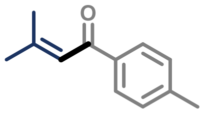

18

3-methyl-1-(p-tolyl)but-2-en-1-one (18). Prepared according to GP1 from 1-bromo-2-methylprop-1-ene (27 mg, 0.2 mmol, 1.0 equiv) and 2-oxo-2-(p-tolyl)acetic acid (66 mg, 0.4 mmol, 2.0 equiv) with phthalimide (29 mg, 0.2 mmol, 1.0 equiv), DBU (60 µl, 61 mg, 0.4 mmol, 2.0 equiv), gCN (10 mg), NiBr2.glyme (3.1 mg, 10 µmol, 5 mol %) and 2.2'-bipyridine (2.3 mg, 15 µmol, 7.5 mol %) for 16 h. Purified via flash column chromatography on silica gel (pentane:EtOAc 80:1) to afford the product as a white solid (10 mg, 29% yield).

Characterization data in accordance with literature.[18]

^{1}H NMR (300 MHz, $CDCl_3$) δ 7.8 (d, J = 8.22 Hz, 2H), 7.2 (d, J = 7.84 Hz, 2H), 6.7 (p, J = 1.29 Hz, 1H), 2.4 (s, 3H), 2.2 (d, J = 1.30 Hz, 3H), 2.0 (d, J = 1.29 Hz, 3H).

^{13}C NMR (101 MHz, $CDCl_3$) δ 191.4, 156.0, 143.1, 136.9, 129.3, 128.5, 121.4, 28.1, 21.7, 21.3.

GCMS (m/z): [M]+ calcd. for $C_{12}H_{14}O$: 174.10, mass found: 174.10.

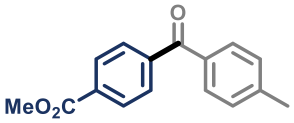

19

methyl 4-(4-methylbenzoyl)benzoate (19). Prepared according to GP1 from methyl 4-bromobenzoate (43 mg, 0.2 mmol, 1.0 equiv) and 2-oxo-2-(p-tolyl)acetic acid (66 mg, 0.4 mmol, 2.0 equiv) with phthalimide (29 mg, 0.2 mmol, 1.0 equiv), Cs_2CO_3 (130 mg, 0.4 mmol, 2.0 equiv), gCN (10 mg), $NiBr_2$.glyme (3.1 mg, 10 µmol, 5 mol %) and 2.2'-bipyridine (2.3 mg, 15 µmol, 7.5 mol %) for 16 h. Purified via flash column chromatography on silica gel (pentane:EtOAc 30:1) to afford the product as a white solid (47 mg, 93% yield).

Characterization data in accordance with literature.[10]

^{1}H NMR (300 MHz, $CDCl_3$) δ 8.12 (d, J = 8.7 Hz, 2H), 7.80 (d, J = 8.6 Hz, 2H), 7.70 (d, J = 8.2 Hz, 2H), 7.27 (d, J = 7.8 Hz, 2H), 3.94 (s, 3H), 2.42 (s, 3H).

^{13}C NMR (75 MHz, $CDCl_3$) δ 195.7, 166.4, 143.9, 141.8, 134.3, 133.0, 130.4, 129.7, 129.5, 129.2, 52.5, 21.7.

GCMS (m/z): [M]+ calcd. for $C_{16}H_{14}O_3$: 254.09, mass found: 254.00.

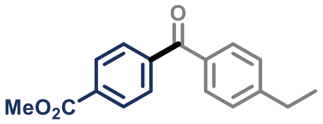
20

methyl 4-(4-ethylbenzoyl)benzoateate (20). Prepared according to GP1 from methyl 4-bromobenzoate (43 mg, 0.2 mmol, 1.0 equiv) and 2-(4-ethylphenyl)-2-oxoacetic acid (71 mg, 0.4 mmol, 2.0 equiv) with phthalimide (29 mg, 0.2 mmol, 1.0 equiv), Cs_2CO_3 (130 mg, 0.4 mmol, 2.0 equiv), gCN (10 mg), $NiBr_2$.glyme (3.1 mg, 10 µmol, 5 mol %) and 2.2'-bipyridine (2.3 mg, 15 µmol, 7.5 mol %) for 16 h. Purified via flash column chromatography on silica gel (pentane:EtOAc 20:1) to afford the product as a white solid (50 mg, 94% yield).

^{1}H NMR (400 MHz, $CDCl_3$) δ 8.13 (d, J = 8.5 Hz, 2H), 7.81 (d, J = 8.4 Hz, 2H), 7.73 (d, J = 8.2 Hz, 2H), 7.31 (d, J = 8.0 Hz, 2H), 3.95 (s, 3H), 2.73 (q, J = 7.6 Hz, 2H), 1.28 (t, J = 7.6 Hz, 3H).

^{13}C NMR (101 MHz, $CDCl_3$) δ 195.8, 166.5, 150.2, 141.8, 134.6, 130.5, 133.1, 129.8, 129.5, 128.1, 52.5, 29.1, 15.3.

HRMS Mass calculated for $C_{17}H_{16}O_3$ $[M]^+$: 268.1099, mass found: 268.1099.

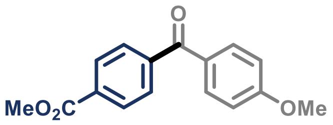
21

methyl 4-(4-methoxybenzoyl)benzoate (21). Prepared according to GP1 from methyl 4-bromobenzoate (43 mg, 0.2 mmol, 1.0 equiv) and 2-(4-methoxyphenyl)-2-oxoacetic acid (108 mg, 0.6 mmol, 3.0 equiv) with phthalimide (29 mg, 0.2 mmol, 1.0 equiv), Cs_2CO_3 (195 mg, 0.6 mmol, 3.0 equiv), gCN (10 mg), $NiBr_2$.glyme (3.1 mg, 10 µmol, 5 mol %) and 2.2'-bipyridine (2.3 mg, 15 µmol, 7.5 mol %) for 16 h. Purified via flash column chromatography on silica gel (pentane:EtOAc 20:1) to afford the product as a yellow solid (47 mg, 93% yield).

Characterization data in accordance with literature.[19]

^{1}H NMR (400 MHz, $CDCl_3$) δ 8.13 (d, J = 8.2 Hz, 2H), 7.86-7.74 (m, 4H), 6.97 (d, J = 8.8 Hz, 2H), 3.96 (s, 3H), 3.89 (s, 3H).

^{13}C NMR (101 MHz, $CDCl_3$) δ 194.9, 166.5, 163.7, 142.3, 132.9, 132.7, 129.7, 129.6, 113.9, 55.7, 52.5.

GCMS (m/z): [M]+ calcd. for $C_{16}H_{14}O_4$: 270.08, mass found: 270.10.

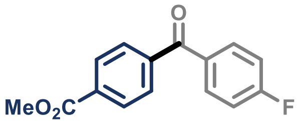
22

methyl 4-(4-fluorobenzoyl)benzoate (22). Prepared according to GP1 from methyl 4-bromobenzoate (86 mg, 0.4 mmol, 1.0 equiv) and 2-(4-fluorophenyl)-2-oxoacetic acid (134 mg, 0.8 mmol, 2.0 equiv) with phthalimide (58 mg, 0.4 mmol, 1.0 equiv), Cs_2CO_3 (260 mg, 0.8 mmol, 2.0 equiv), gCN (10 mg), $NiBr_2$.glyme (6.2 mg, 20 µmol, 5 mol %) and 2.2'-bipyridine (4.6 mg, 30 µmol, 7.5 mol %) for 16 h. Purified via flash column chromatography on silica gel (pentane:EtOAc 20:1) to afford the product as a white solid (56 mg, 50% yield).

Characterization data in accordance with literature.[20]

^{1}H NMR (300 MHz, $CDCl_3$) δ 8.15 (d, J = 8.7 Hz, 2H), 7.89 – 7.77 (m, 4H), 7.18 (t, J = 8.6 Hz, 2H), 3.97 (s, 3H).

^{13}C NMR (75 MHz, $CDCl_3$) δ 194.7, 167.5, 166.4, 164.1, 141.4, 133.4, 133.3 (d, J = 3.17 Hz), 132.9 (d, J = 9.28 Hz), 129.7 (d, J = 1.55 Hz), 115.9 (d, J = 22.02 Hz), 52.6.

^{19}F NMR (282 MHz, $CDCl_3$) δ -104.85.

GCMS (m/z): [M]+ calcd. for $C_{15}H_{11}FO_3$: 258.06, mass found: 258.00.

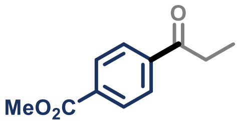

23

methyl 4-propionylbenzoate (23). Prepared according to GP1 from methyl 4-bromobenzoate (43 mg, 0.2 mmol, 1.0 equiv) and 2-oxobutanoic acid (41 mg, 0.4 mmol, 2.0 equiv) with phthalimide (29 mg, 0.2 mmol, 1.0 equiv), Cs_2CO_3 (130 mg, 0.4 mmol, 2.0 equiv), gCN (10 mg), $NiBr_2$.glyme (3.1 mg, 10 µmol, 5 mol %) and 2.2'-bipyridine (2.3 mg, 15 µmol, 7.5 mol %) for 16 h. Purified via flash column chromatography on silica gel (pentane:EtOAc 30:1) to afford the product as a white solid (17 mg, 43% yield).

Characterization data in accordance with literature.[21]

^{1}H NMR (400 MHz, $CDCl_3$) δ 8.11 (d, J = 8.6 Hz, 2H), 8.00 (d, J = 8.4 Hz, 2H), 3.94 (s, 3H), 3.03 (q, J = 7.2 Hz, 2H), 1.23 (t, J = 7.1 Hz, 3H).

^{13}C NMR (101 MHz, $CDCl_3$) δ 200.4, 166.4, 140.3, 133.8, 129.9, 128.0, 52.6, 32.3, 8.2.

GCMS (m/z): [M]+ calcd. for $C_{11}H_{12}O_3$: 192.08, mass found: 192.10

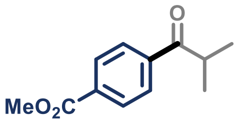

24

methyl 4-isobutyrylbenzoate (24). Prepared according to GP1 from methyl 4-bromobenzoate (43 mg, 0.2 mmol, 1.0 equiv) and 3-methyl-2-oxobutanoic acid (46 mg, 0.4 mmol, 2.0 equiv) with phthalimide (29 mg, 0.2 mmol, 1.0 equiv), Cs_2CO_3 (130 mg, 0.4 mmol, 2.0 equiv), gCN (10 mg), $NiBr_2$.glyme (3.1 mg, 10 µmol, 5 mol %) and 2.2'-bipyridine (2.3 mg, 15 µmol, 7.5 mol %) for 16 h. Purified via flash column chromatography on silica gel (pentane:EtOAc 30:1) to afford the product as a colourless oil (18 mg, 44% yield).

Characterization data in accordance with literature.[22]

^{1}H NMR 400 MHz, $CDCl_3$) δ 8.12 (d, J = 8.5 Hz, 2H), 7.99 (d, J = 8.5 Hz, 2H), 3.95 (s, 3H), 3.55 (hept, J = 6.8 Hz, 1H), 1.22 (d, J = 6.9 Hz, 6H).

^{13}C NMR (101 MHz, $CDCl_3$) δ 204.1, 166.4, 139.7, 133.7, 130.0, 128.3, 52.6, 36.0, 19.1.

GCMS (m/z): [M]+ calcd. for $C_{12}H_{14}O_3$: 206.09, mass found: 206.10

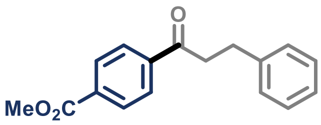

25

methyl 4-(3-phenylpropanoyl)benzoate (25). Prepared according to GP1 from methyl 4-bromobenzoate (43 mg, 0.2 mmol, 1.0 equiv) and 2-oxo-2-phenyl-butanoic acid (63 mg, 0.4 mmol, 2.0 equiv) with phthalimide (29 mg, 0.2 mmol, 1.0 equiv), Cs_2CO_3 (130 mg, 0.4 mmol, 2.0 equiv), gCN (10 mg), $NiBr_2$.glyme (3.1 mg, 10 µmol, 5 mol %) and 2.2'-bipyridine (2.3 mg, 15 µmol, 7.5 mol %) for 16 h, under use of 456 nm irradiation. Purified via flash column chromatography on silica gel (pentane:EtOAc 30:1) to afford the product as a white solid (50 mg, 93% yield).

Characterization data in accordance with literature.[23]

^{1}H NMR (300 MHz, $CDCl_3$) δ 8.11 (d, J = 8.7 Hz, 2H), 8.00 (d, J = 8.7 Hz, 2H), 7.37 – 7.15 (m, 5H), 3.95 (s, 3H), 3.33 (t, J = 7.6 Hz, 2H), 3.08 (t, J = 7.6 Hz, 2H).

^{13}C NMR (101 MHz, $CDCl_3$) δ 198.9, 166.4, 141.1, 140.2, 134.0, 130.0, 128.7, 128.6, 128.1, 126.4, 52.6, 41.0, 30.1.

GCMS (m/z): [M]+ calcd. for $C_{17}H_{16}O_3$: 268.10, mass found: 268.10

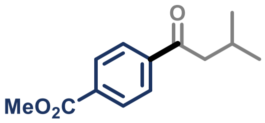

26

methyl 4-(3-methylbutanoyl)benzoate (26). Prepared according to GP1 from methyl 4-bromobenzoate (43 mg, 0.2 mmol, 1.0 equiv) and 4-methyl-2-oxopentanoic acid (52 mg, 0.4 mmol, 2.0 equiv) with phthalimide (29 mg, 0.2 mmol, 1.0 equiv), Cs_2CO_3 (130 mg, 0.4 mmol, 2.0 equiv), gCN (10 mg), $NiBr_2$.glyme (3.1 mg, 10 µmol, 5 mol %) and 2.2'-bipyridine (2.3 mg, 15 µmol, 7.5 mol %) for 16 h, under use of 456 nm irradiation. Purified via flash column chromatography on silica gel (pentane:EtOAc 30:1) to afford the product as a white solid (24 mg, 54% yield).

Characterization data in accordance with literature.[24]

^{1}H NMR (400 MHz, $CDCl_3$) δ 8.11 (d, J = 7.8 Hz, 2H), 7.98 (d, J = 7.9 Hz, 2H), 3.94 (s, 3H), 2.85 (d, J = 6.7 Hz, 2H), 2.28 (tq, J = 14.4, 6.8 Hz, 1H), 1.00 (d, J = 6.6 Hz, 6H).

^{13}C NMR (101 MHz, $CDCl_3$) δ 199.8, 166.4, 140.7, 133.8, 129.9, 128.1, 52.6, 47.9, 25.2, 22.8.

GCMS (m/z): [M]+ calcd. for $C_{13}H_{16}O_3$: 220.11, mass found: 220.10

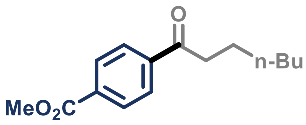

27

methyl 4-heptanoylbenzoate (27). Prepared according to GP1 from methyl 4-bromobenzoate (43 mg, 0.2 mmol, 1.0 equiv) and 2-oxooctanoic acid (63 mg, 0.4 mmol, 2.0 equiv) with phthalimide (29 mg, 0.2 mmol, 1.0 equiv), Cs_2CO_3 (130 mg, 0.4 mmol, 2.0 equiv), gCN (10 mg), $NiBr_2$.glyme (3.1 mg, 10 µmol, 5 mol %) and 2.2'-bipyridine (2.3 mg, 15 µmol, 7.5 mol %) for 16 h, under use of 456 nm irradiation. Purified via flash column chromatography on silica gel (pentane:DCM 1:1) to afford the product as a white solid (18 mg, 46% yield).

Characterization data in accordance with literature.[25]

^{1}H NMR (300 MHz, $CDCl_3$) δ 8.12 (d, J = 8.4 Hz, 2H), 8.00 (d, J = 8.6 Hz, 2H), 3.95 (s, 3H), 2.98 (t, J = 7.4 Hz, 2H), 1.74 (p, J = 7.4 Hz, 2H), 1.45 – 1.23 (m, 6H), 1.11 – 0.78 (m, 3H).

^{13}C NMR (75 MHz, $CDCl_3$) δ 200.2, 166.4, 140.5, 133.8, 130.0, 128.1, 52.6, 39.1, 31.8, 29.1, 24.3, 22.7, 14.2.

GCMS (m/z): [M]+ calcd. for $C_{15}H_{20}O_3$: 248.14, mass found: 248.10

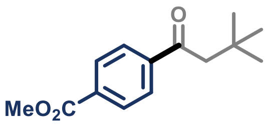

28

methyl 4-(3,3-dimethylbutanoyl)benzoate (28). Prepared according to GP1 from methyl 4-bromobenzoate (43 mg, 0.2 mmol, 1.0 equiv) and 4,4-dimethyl-2-oxopentanoic acid (63 mg, 0.4 mmol, 2.0 equiv) with phthalimide (29 mg, 0.2 mmol, 1.0 equiv), Cs_2CO_3 (130 mg, 0.4 mmol, 2.0 equiv), gCN (10 mg), $NiBr_2$.glyme (3.1 mg, 10 µmol, 5 mol %) and 2.2'-bipyridine (2.3 mg, 15 µmol, 7.5 mol %) for 16, h under use of 456 nm irradiation. Purified via flash column chromatography on silica gel (pentane:EtOAc 20:1) to afford the product as a white solid (34 mg, 68% yield).

^{1}H NMR (300 MHz, $CDCl_3$) δ 8.10 (d, J = 8.6 Hz, 2H), 7.97 (d, J = 8.7 Hz, 2H), 3.94 (s, 3H), 2.88 (s, 2H), 1.06 (s, 9H).

^{13}C NMR (101 MHz, $CDCl_3$) δ 200.1, 166.4, 141.9, 133.7, 129.9, 128.2, 52.6, 50.6, 31.6, 30.2.

GCMS (m/z): [M]+ calcd. for $C_{14}H_{18}O_3$: 234.14, mass found: 234.10

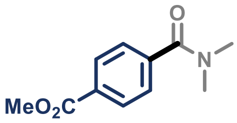

29

methyl 4-(dimethylcarbamoyl)benzoate (29). Prepared according to GP1 from methyl 4-bromobenzoate (43 mg, 0.2 mmol, 1.0 equiv) and 2-(dimethylamino)-2-oxoacetic acid (47 mg, 0.4 mmol, 2.0 equiv) with phthalimide (29 mg, 0.2 mmol, 1.0 equiv), Cs_2CO_3 (130 mg, 0.4 mmol, 2.0 equiv), gCN (10 mg), $NiBr_2$.glyme (3.1 mg, 10 µmol, 5 mol %) and 2.2'-bipyridine (2.3 mg, 15 µmol, 7.5 mol %) for 16 h. Purified via flash column chromatography on silica gel (pentane:EtOAc 1:1) to afford the product as a clear oil (28 mg, 68% yield).

Characterization data in accordance with literature.[26]

^{1}H NMR (300 MHz, $CDCl_3$) δ 8.06 (d, J = 8.4 Hz, 2H), 7.46 (d, J = 8.4 Hz, 2H), 3.92 (s, 3H), 3.11 (s, 3H), 2.94 (s, 3H).

^{13}C NMR (75 MHz, $CDCl_3$) δ 170.7, 166.5, 140.8, 131.1, 129.8, 127.1, 52.4, 39.5, 35.4.

GCMS (m/z): [M]+ calcd. for $C_{11}H_{13}NO_3$: 207.09, mass found: 207.00

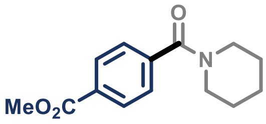

30

methyl 4-(piperidine-1-carbonyl)benzoate (30). Prepared according to GP1 from methyl 4-bromobenzoate (43 mg, 0.2 mmol, 1.0 equiv) and 2-oxo-2-(piperidin-1-yl)acetic acid (63 mg, 0.4 mmol, 2.0 equiv) with phthalimide (29 mg, 0.2 mmol, 1.0 equiv), Cs_2CO_3 (130 mg, 0.4 mmol, 2.0 equiv), gCN (10 mg), $NiBr_2$.glyme (3.1 mg, 10 µmol, 5 mol %) and 2.2'-bipyridine (2.3 mg, 15 µmol, 7.5 mol %) for 16 h. Purified via flash column chromatography on silica gel (pentane:EtOAc 3:1) to afford the product as a yellow oil (30 mg, 61% yield).

Characterization data in accordance with literature.[27]

^{1}H NMR (400 MHz, $CDCl_3$) δ 8.07 (d, J = 8.3 Hz, 2H), 7.45 (d, J = 8.5 Hz, 2H), 3.93 (s, 3H), 3.72 (s, 2H), 3.29 (s, 2H), 1.68 (s, 4H), 1.51 (s, 2H).

^{13}C NMR (101 MHz, $CDCl_3$) δ 169.4, 166.6, 141.0, 131.0, 129.9, 126.9, 52.4, 48.8, 43.2, 26.7, 25.7, 24.7.

GCMS (m/z): $[M_2+Na]^+$ calcd. for $C_{28}H_{34}N_2O_6Na$: 517.23, mass found: 517.23

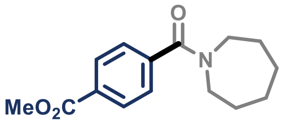

31

methyl 4-(azepane-1-carbonyl)benzoate (31). Prepared according to GP1 from methyl 4-bromobenzoate (43 mg, 0.2 mmol, 1.0 equiv) and 2-(azepan-1-yl)-2-oxoacetic acid (69 mg, 0.4 mmol, 2.0 equiv) with phthalimide (29 mg, 0.2 mmol, 1.0 equiv), Cs_2CO_3 (130 mg, 0.4 mmol, 2.0 equiv), gCN (10 mg), $NiBr_2$.glyme (3.1 mg, 10 µmol, 5 mol %) and 2.2'-bipyridine (2.3 mg, 15 µmol, 7.5 mol %) for 16 h. Purified via flash column chromatography on silica gel (pentane:EtOAc 3:1) to afford the product as a clear oil (35 mg, 67% yield).

Characterization data in accordance with literature.[28]

^{1}H NMR (300 MHz, $CDCl_3$) δ 8.06 (d, J = 8.6 Hz, 2H), 7.42 (d, J = 8.5 Hz, 2H), 3.92 (s, 3H), 3.72 – 3.62 (m, 2H), 3.35 – 3.25 (m, 2H), 1.90 – 1.76 (m, 2H), 1.70 – 1.53 (m, 6H).

^{13}C NMR (101 MHz, $CDCl_3$) δ 170.7, 166.6, 141.8, 130.7, 129.9, 126.6, 52.4, 49.7, 46.5, 29.6, 27.9, 27.3, 26.6.

GCMS (m/z): [M]+ calcd. for $C_{15}H_{19}NO_3$: 261.14, mass found: 261.20.

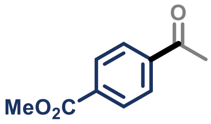

33

methyl 4-acetylbenzoate (33). Prepared according to GP1 from methyl 4-bromobenzoate (43 mg, 0.2 mmol, 1.0 equiv) and 2-oxooctanoic acid (63 mg, 0.4 mmol, 2.0 equiv) with phthalimide (29 mg, 0.2 mmol, 1.0 equiv), Cs_2CO_3 (130 mg, 0.4 mmol, 2.0 equiv), gCN (10 mg), $NiBr_2$.glyme (3.1 mg, 10 µmol, 5 mol %) and 2.2'-bipyridine (2.3 mg, 15 µmol, 7.5 mol %) for 16 h. Purified via flash column chromatography on silica gel (pentane:EtOAc 20:1) to afford the product as a white solid (20 mg, 55% yield).

Characterization data in accordance with literature.[29]

^{1}H NMR (300 MHz, $CDCl_3$) δ 8.12 (d, J = 8.8 Hz, 1H), 8.00 (d, J = 8.7 Hz, 1H), 3.95 (s, 3H), 2.64 (s, 3H).
^{13}C NMR (101 MHz, $CDCl_3$) δ 197.7, 166.4, 140.4, 134.0, 130.0, 128.4, 52.6, 27.0.
GCMS (m/z): [M]+ calcd. for $C_{10}H_{10}O_3$: 178.06, mass found: 178.00

13. Limitations of the scope

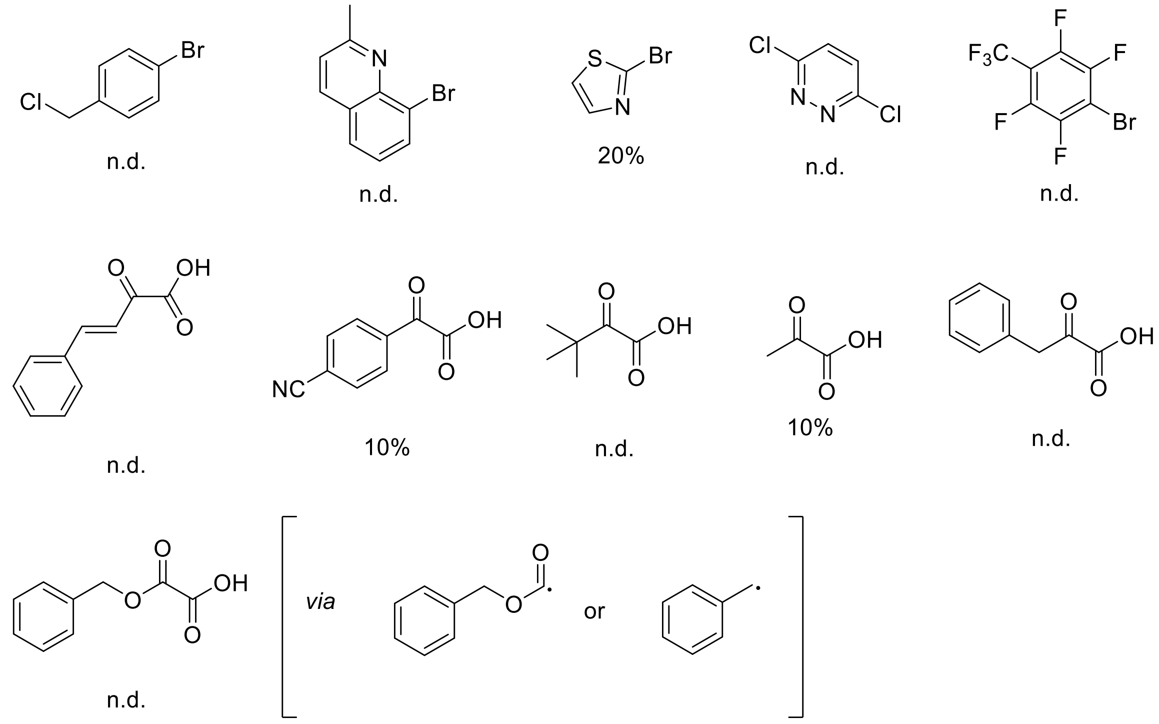

Figure S9: Limitations of the scope

14. NMR spectra of isolated compounds

Methyl 4-benzoylbenzoate (3)

^{1}H (300 MHz, $CDCl_3$)

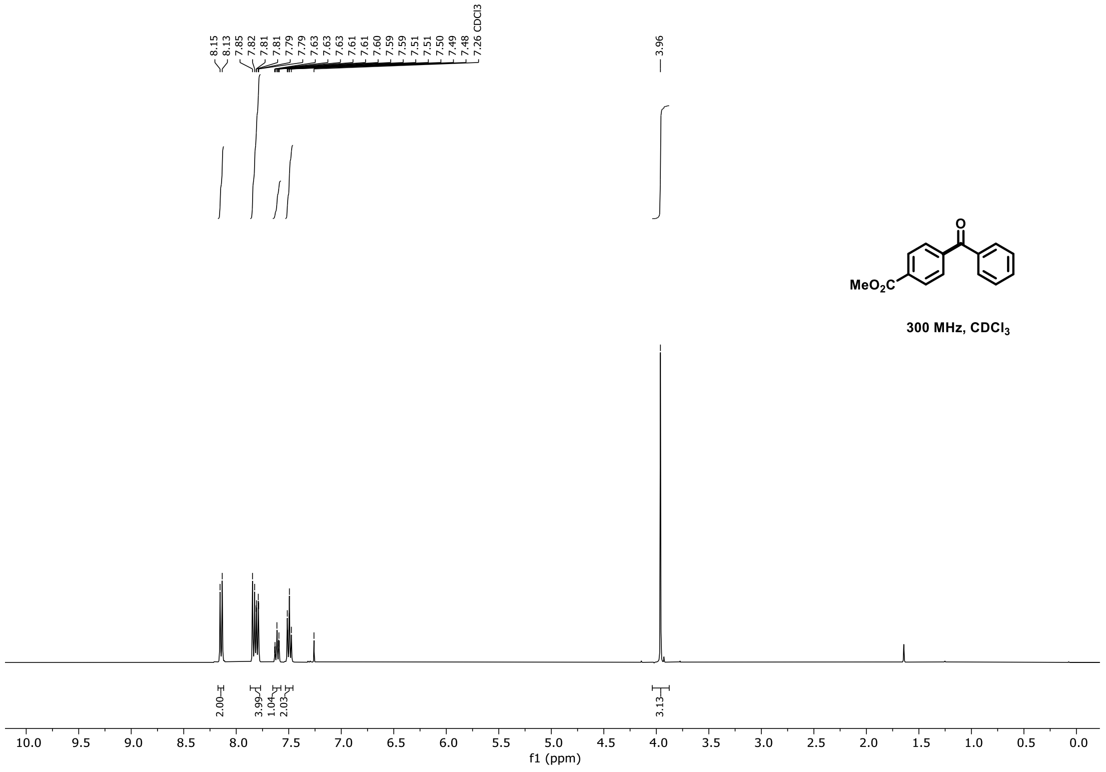

^{13}C (75 MHz, $CDCl_3$)

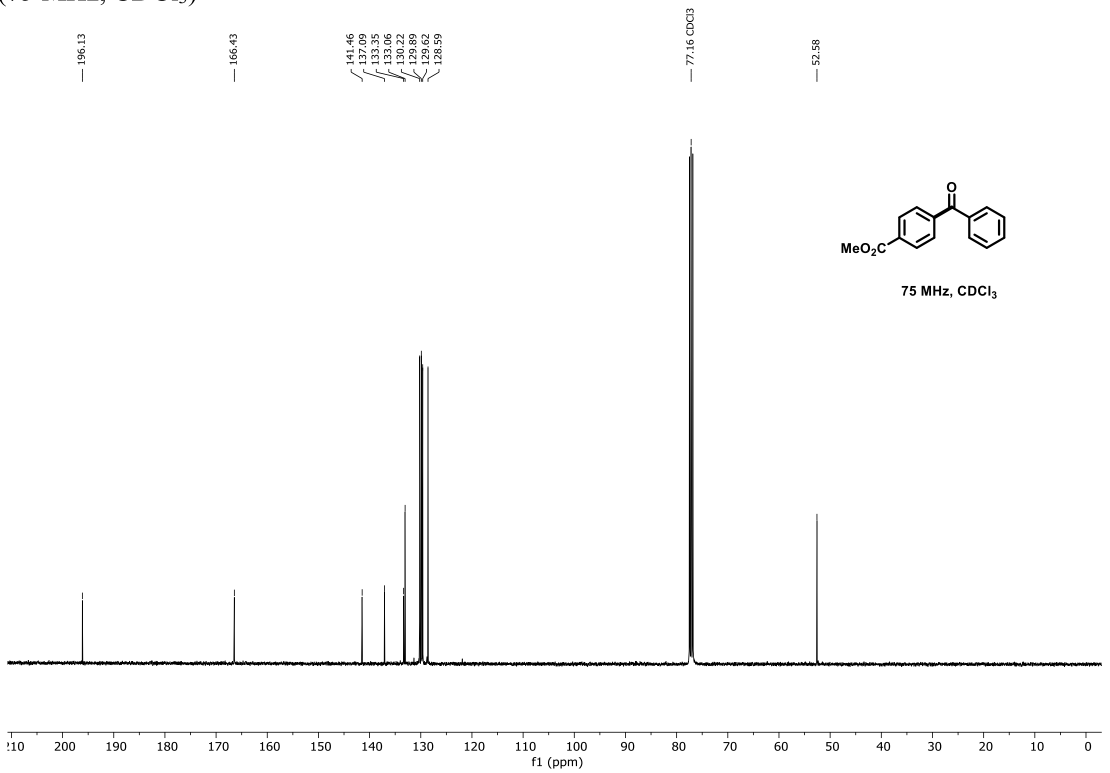

4-(4-Methylbenzoyl)benzonitrile (4)

^{1}H (300 MHz, $CDCl_3$)

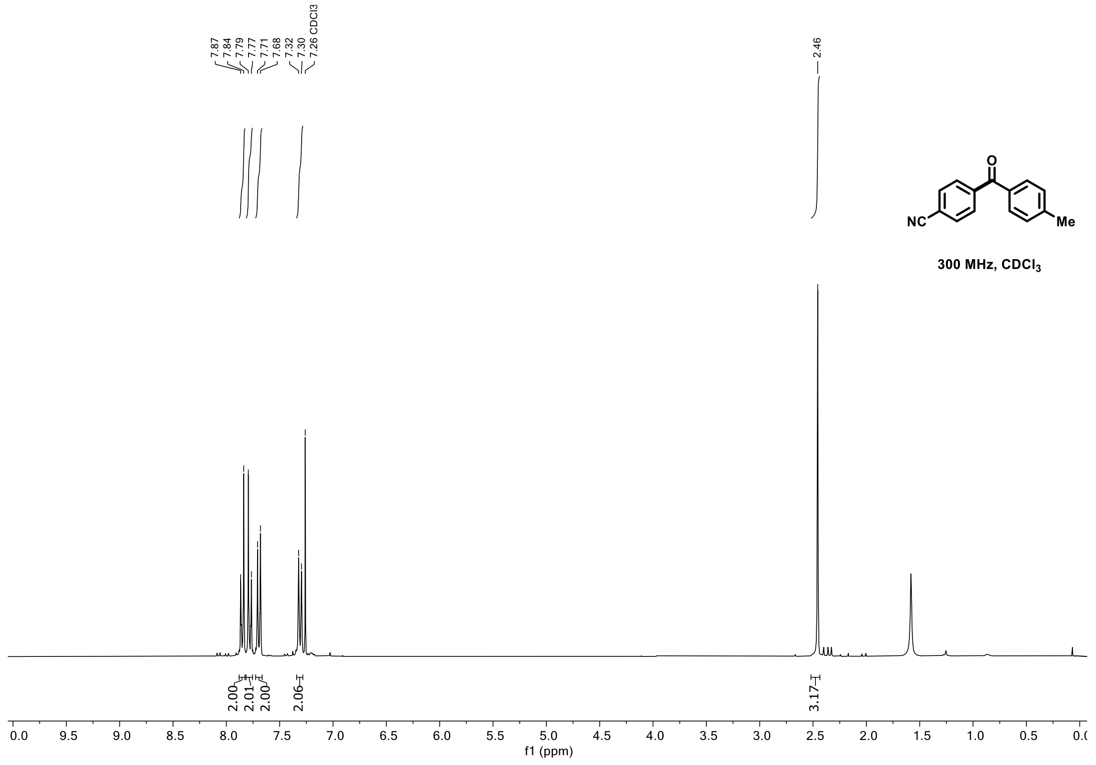

^{13}C (75 MHz, $CDCl_3$)

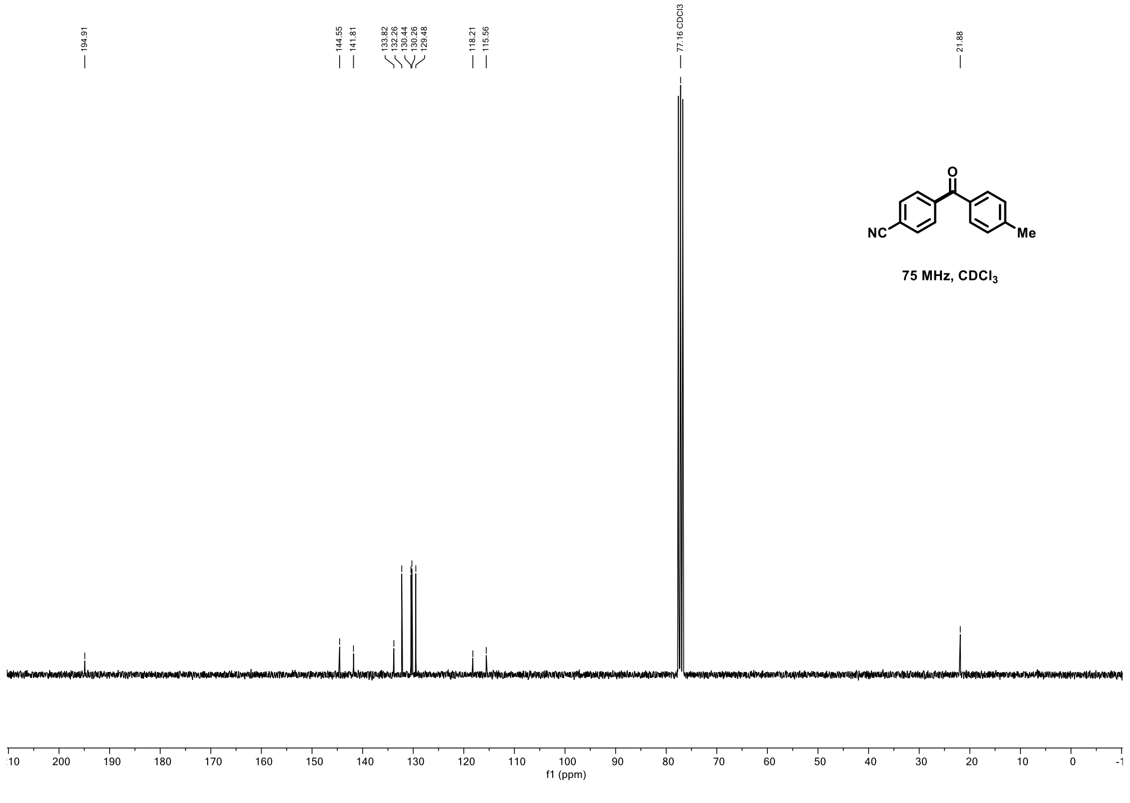

1-(4-(4-Methylbenzoyl)phenyl)ethan-1-one (5)

^{1}H (400 MHz, $CDCl_3$)

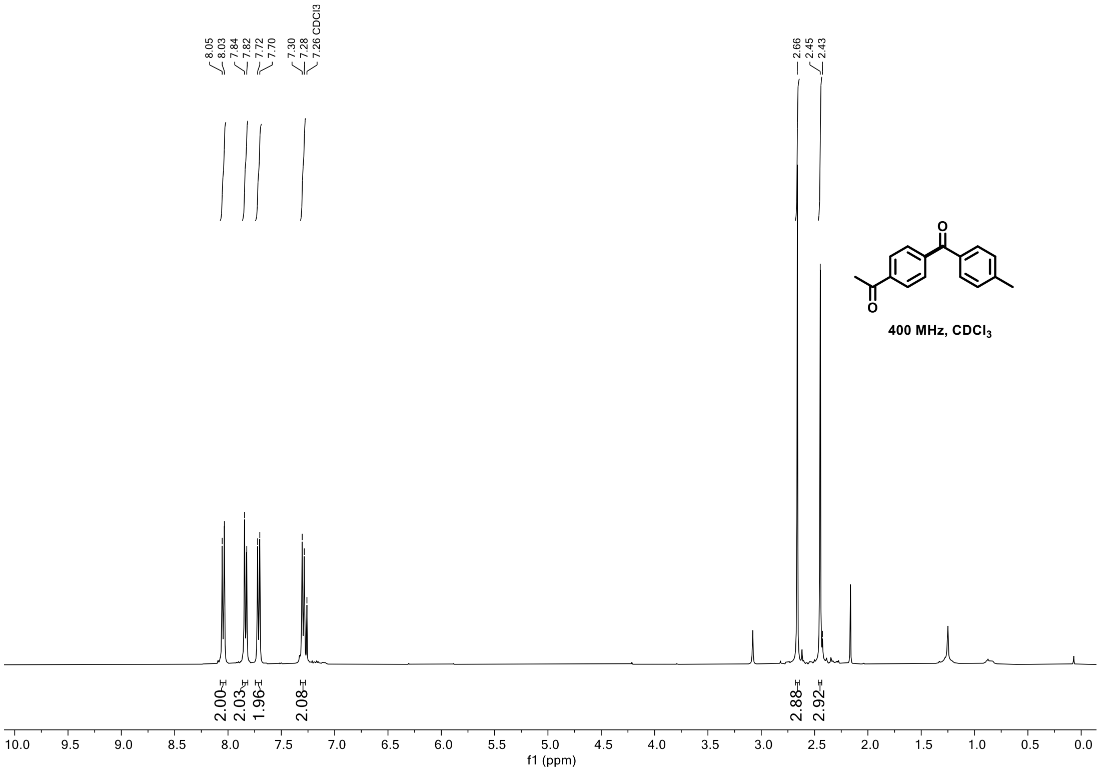

^{13}C (101 MHz, $CDCl_3$)

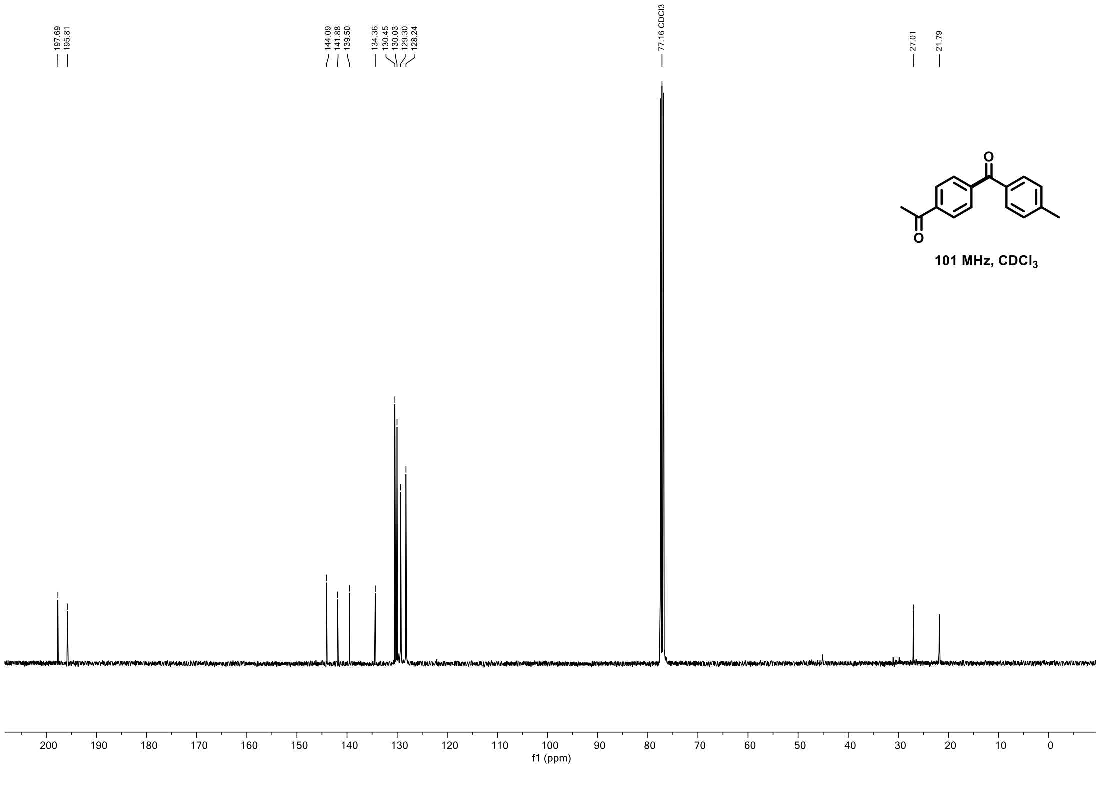

p-Tolyl(4-(trifluoromethyl)phenyl)methanone (6)

^{1}H (400 MHz, $CDCl_3$)

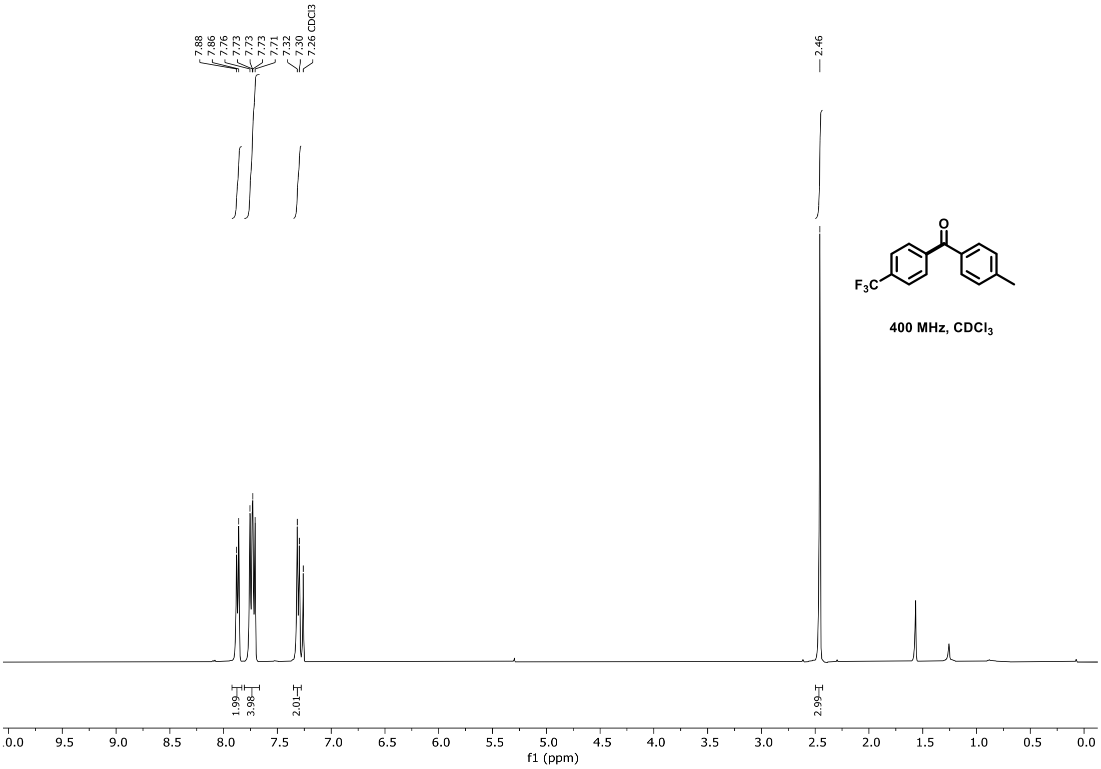

^{13}C (101 MHz, $CDCl_3$)

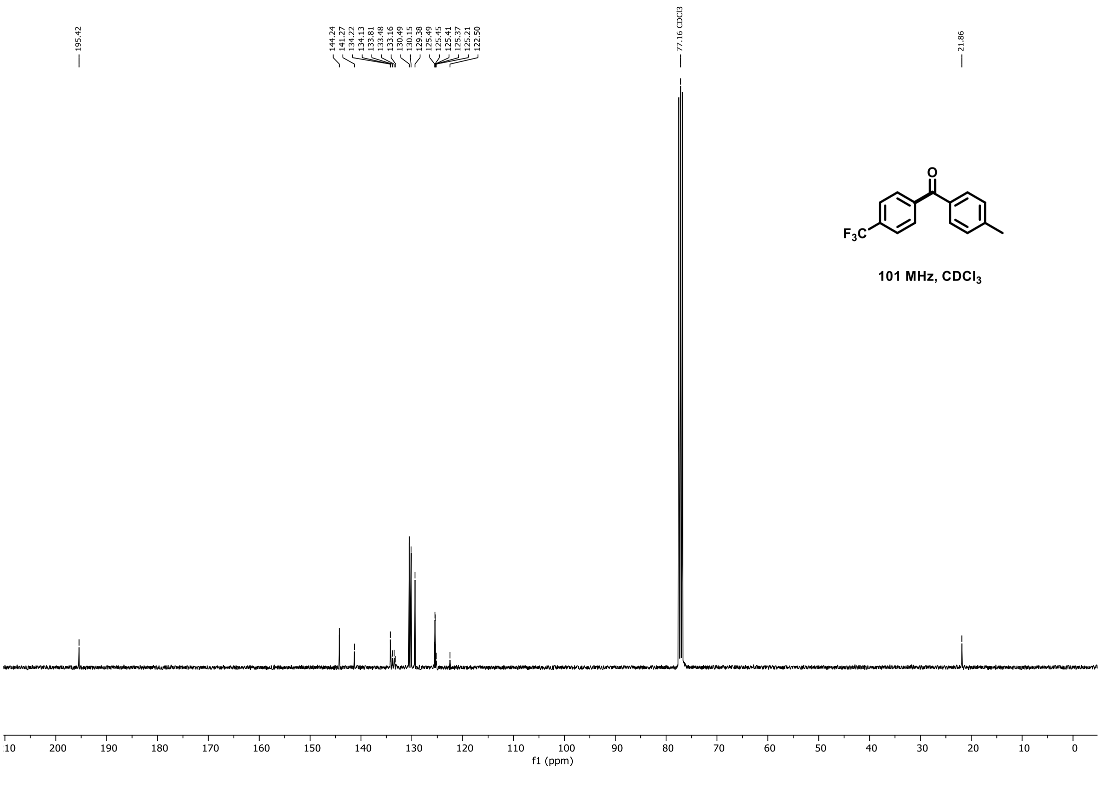

19F (282 MHz, CDCl3)

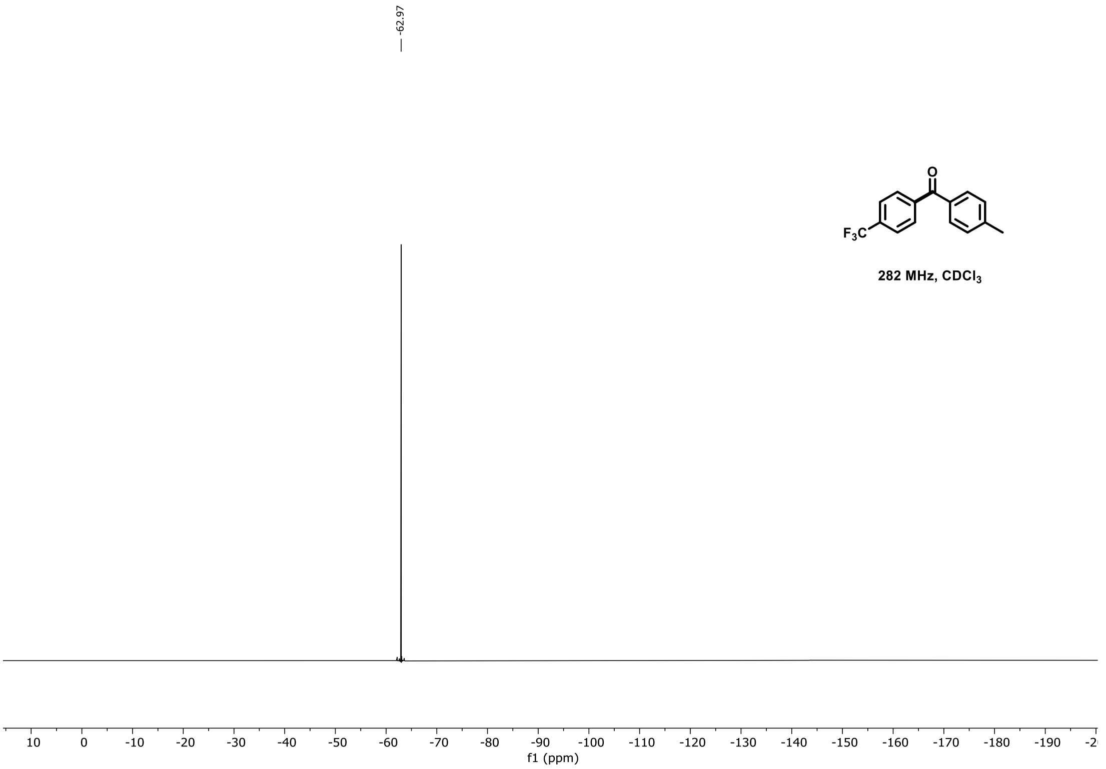

(3,5-bis(Trifluoromethyl)phenyl)(p-tolyl)methanone (7)

^{1}H (300 MHz, $CDCl_3$)

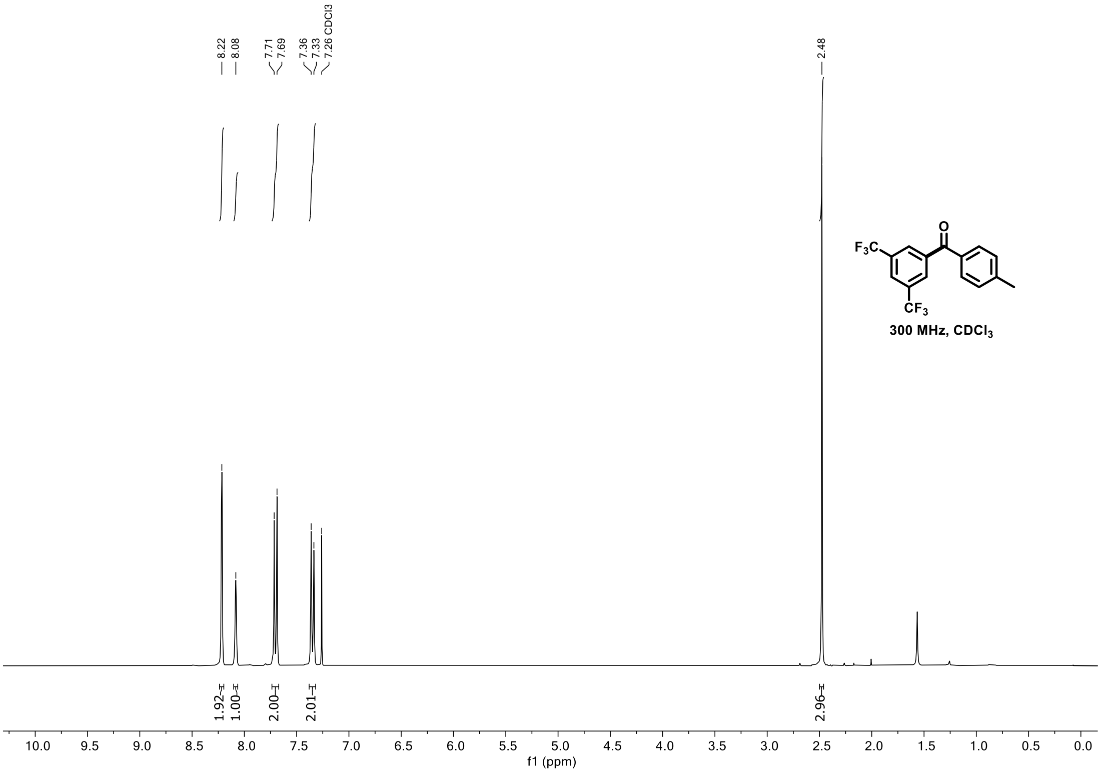

^{13}C (75 MHz, $CDCl_3$)

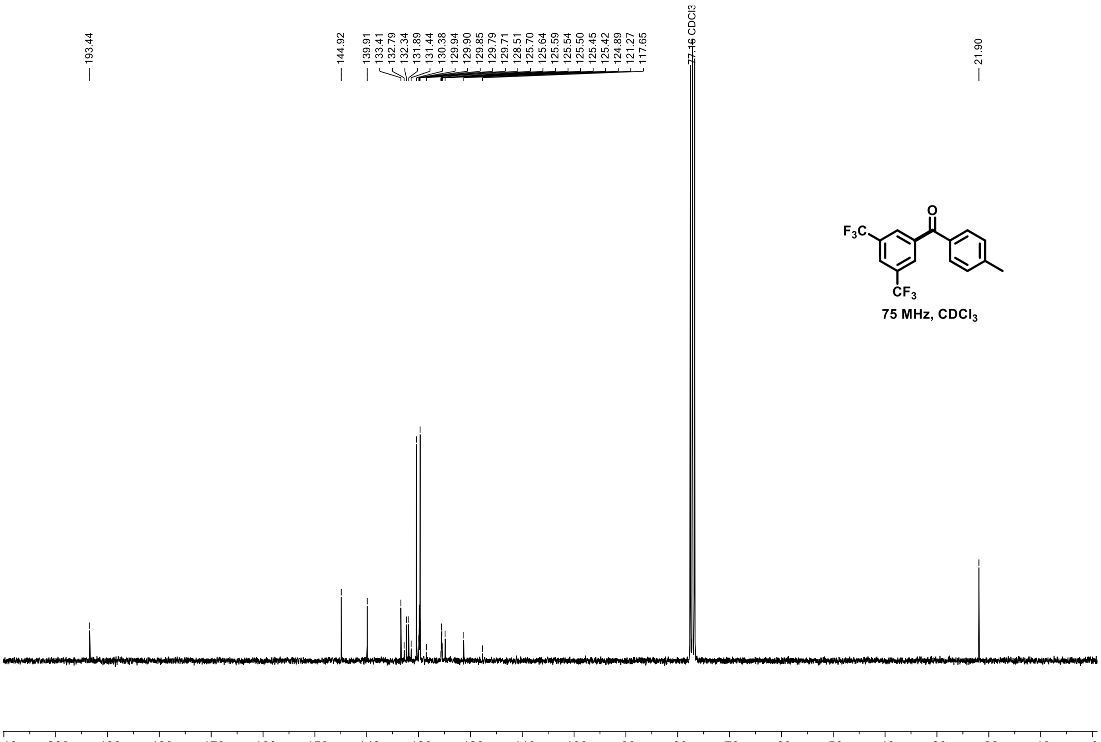

^{19}F (282 MHz, $CDCl_3$)

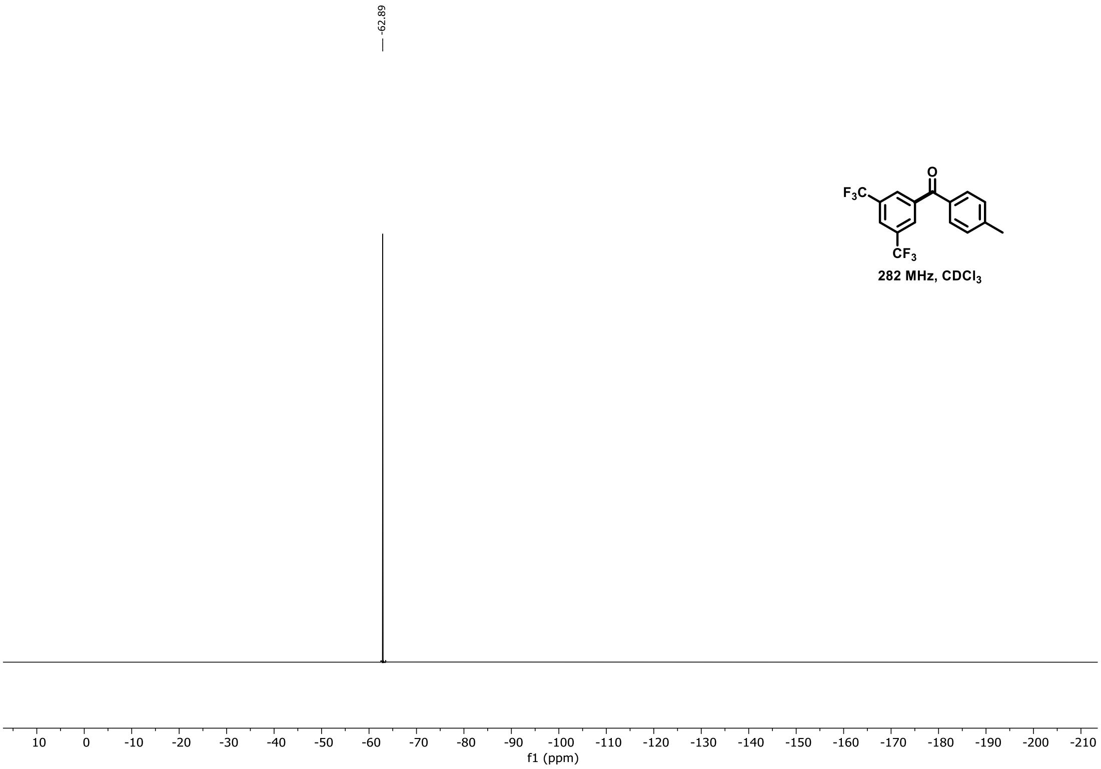

(4-Chlorophenyl)(p-tolyl)methanone (8)

^{1}H (400 MHz, $CDCl_3$)

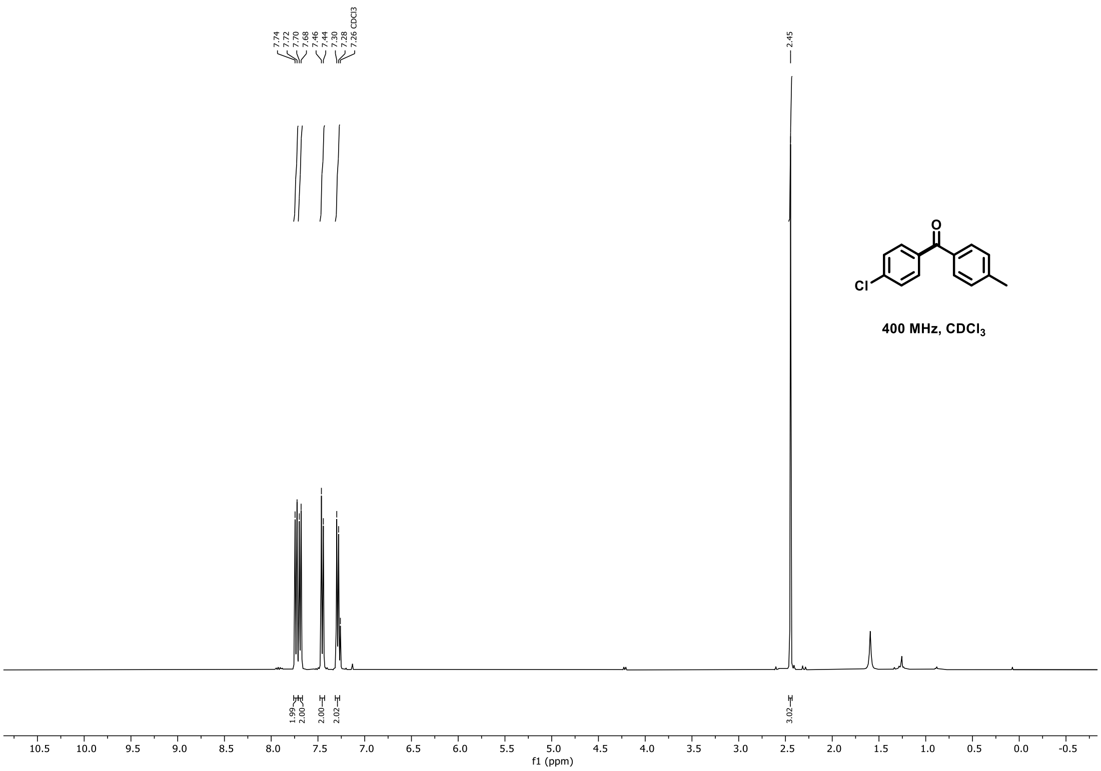

^{13}C (101 MHz, $CDCl_3$)

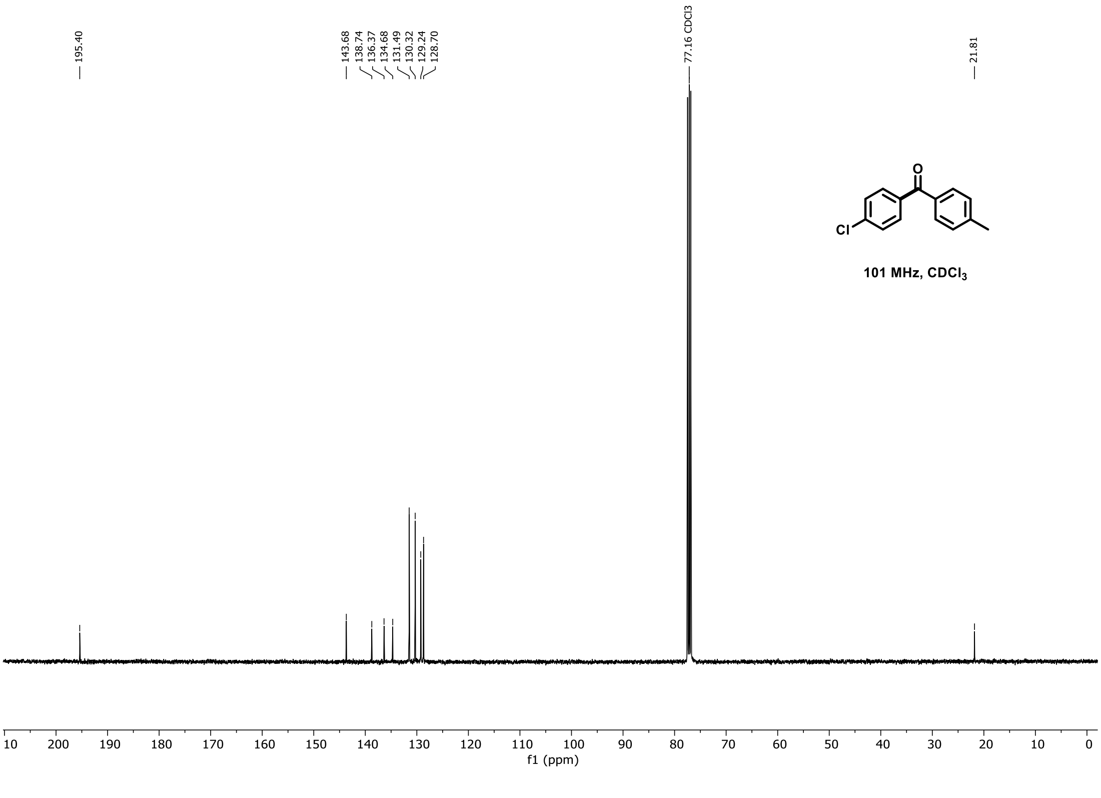

Pyridin-2-yl(p-tolyl)methanone (9)

^{1}H (300 MHz, $CDCl_3$)

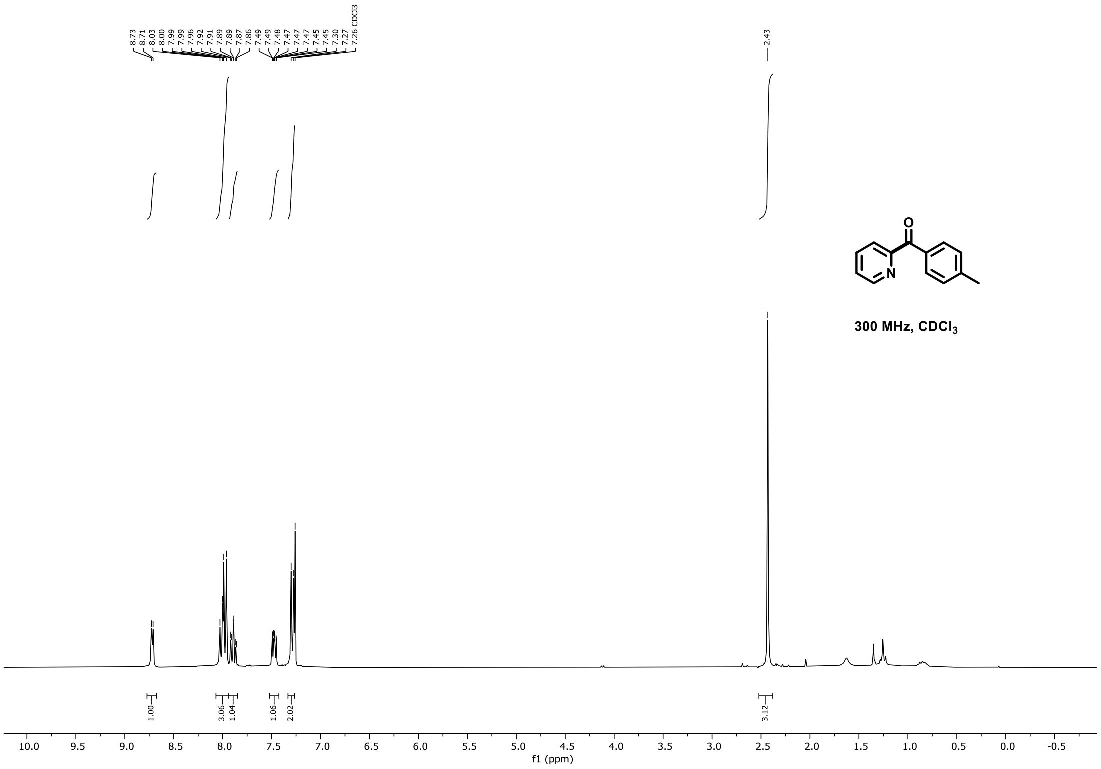

^{13}C (75 MHz, $CDCl_3$)

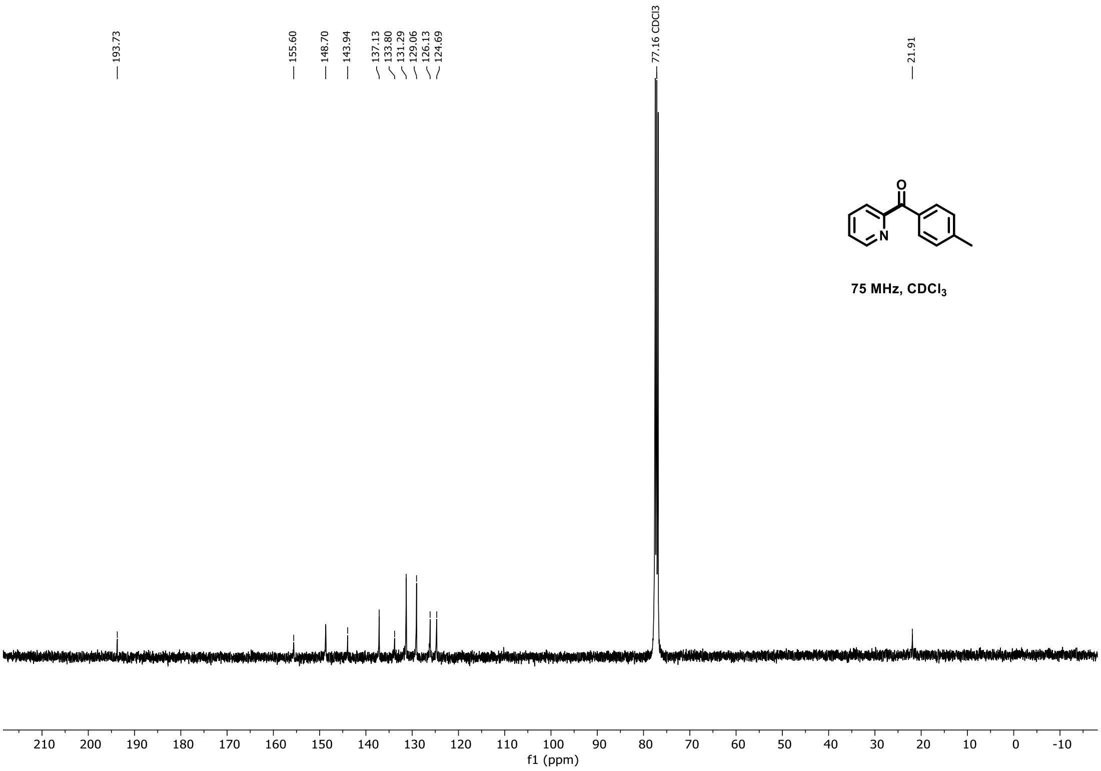

p-Tolyl(6-(trifluoromethyl)pyridin-3-yl)methanone (10)

^{1}H (400 MHz, $CDCl_3$)

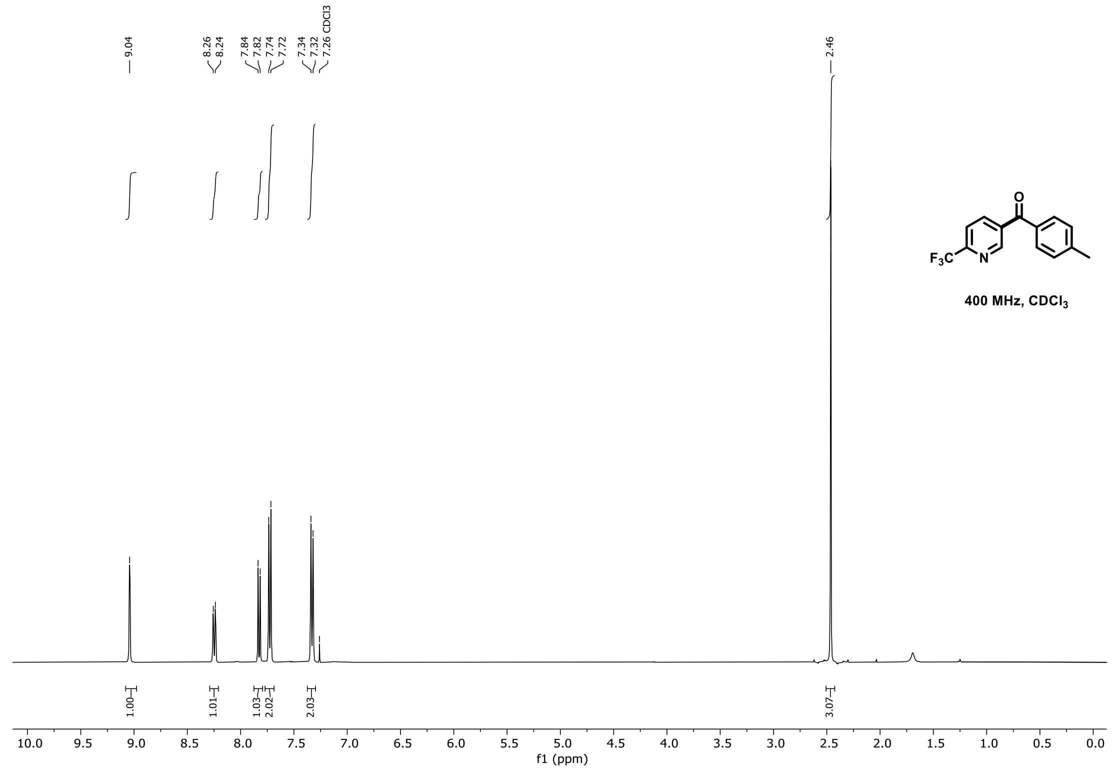

^{13}C (101 MHz, $CDCl_3$)

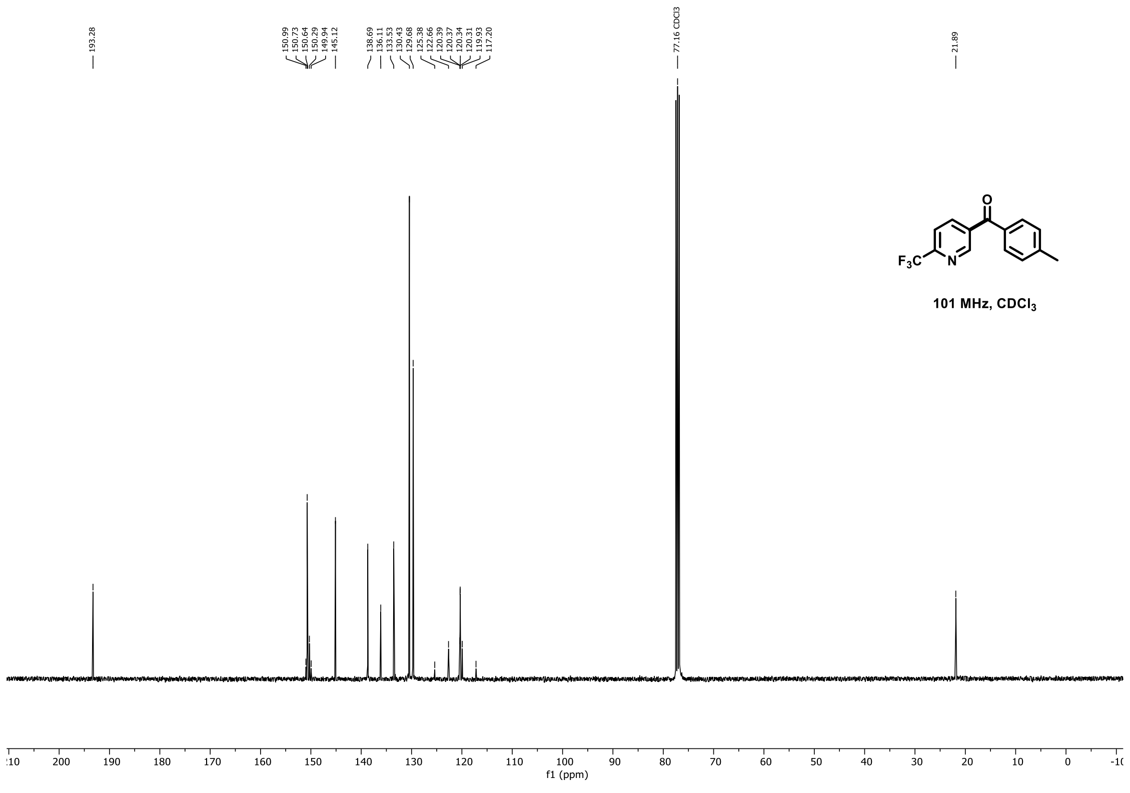

^{19}F (282 MHz, $CDCl_3$)

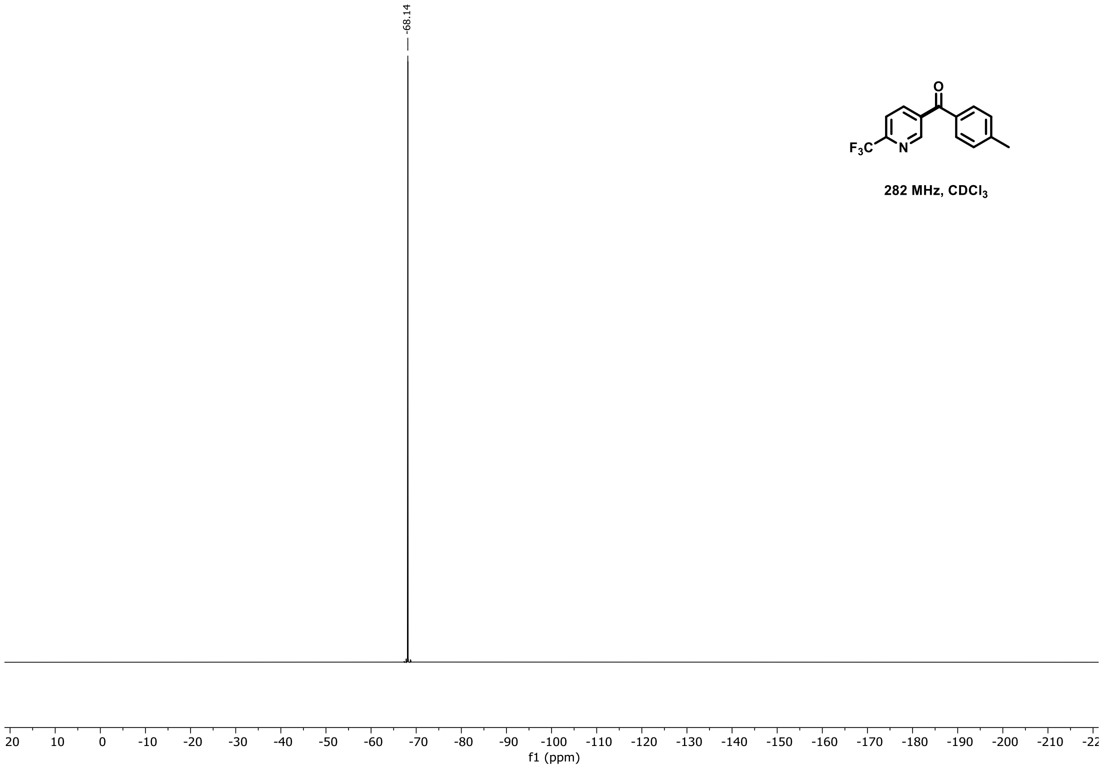

Isoquinolin-3-yl(phenyl)methanone (11)

^{1}H (300 MHz, $CDCl_3$)

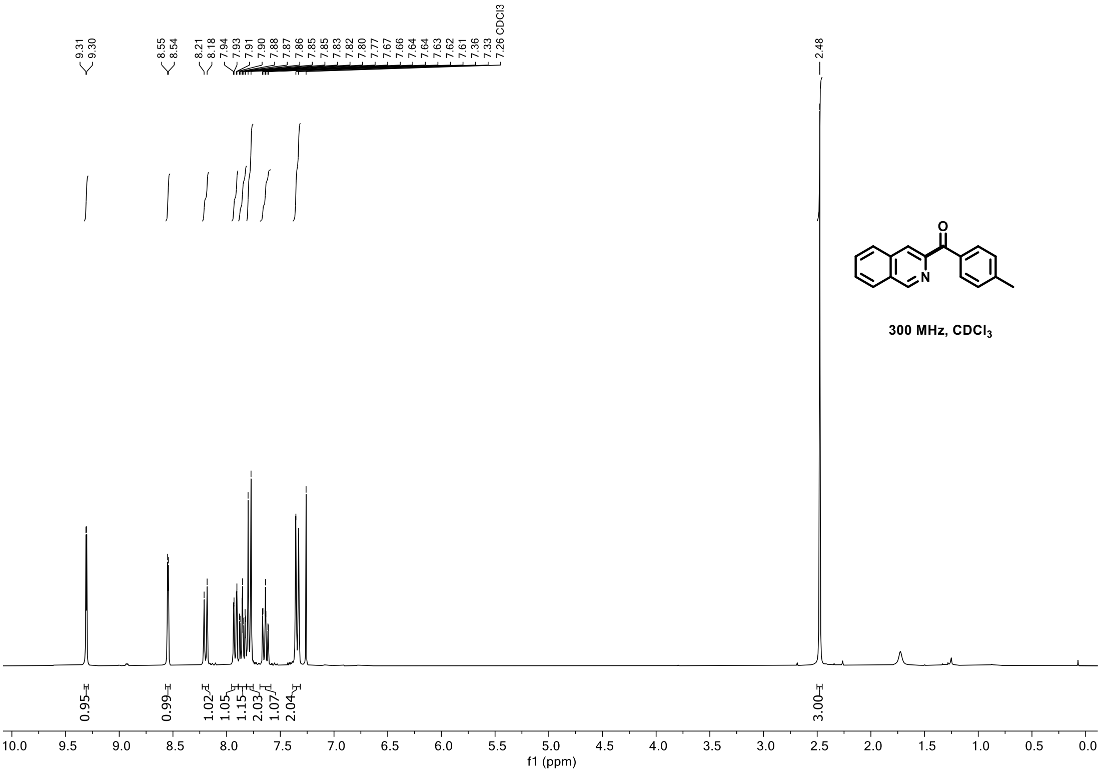

^{13}C (75 MHz, $CDCl_3$)

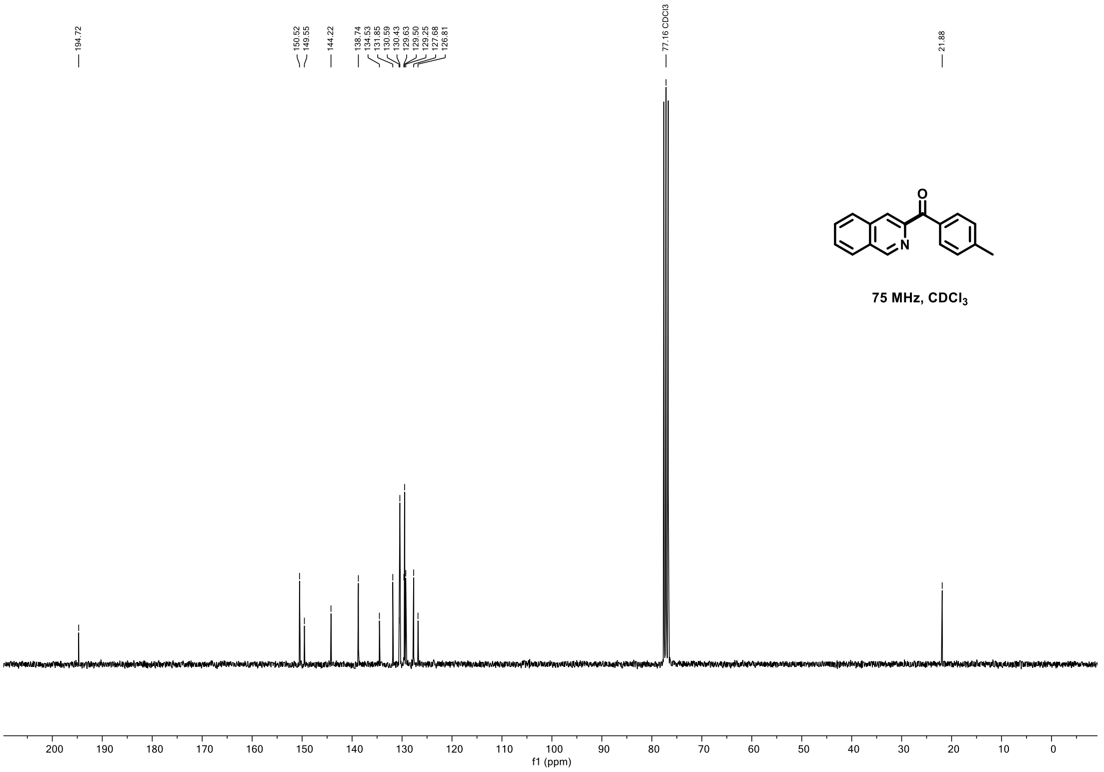

(1-Methyl-1H-benzo[d][1,2,3]triazol-6-yl)(p-tolyl)methanone (12)

^{1}H (400 MHz, CDCl$_3$)

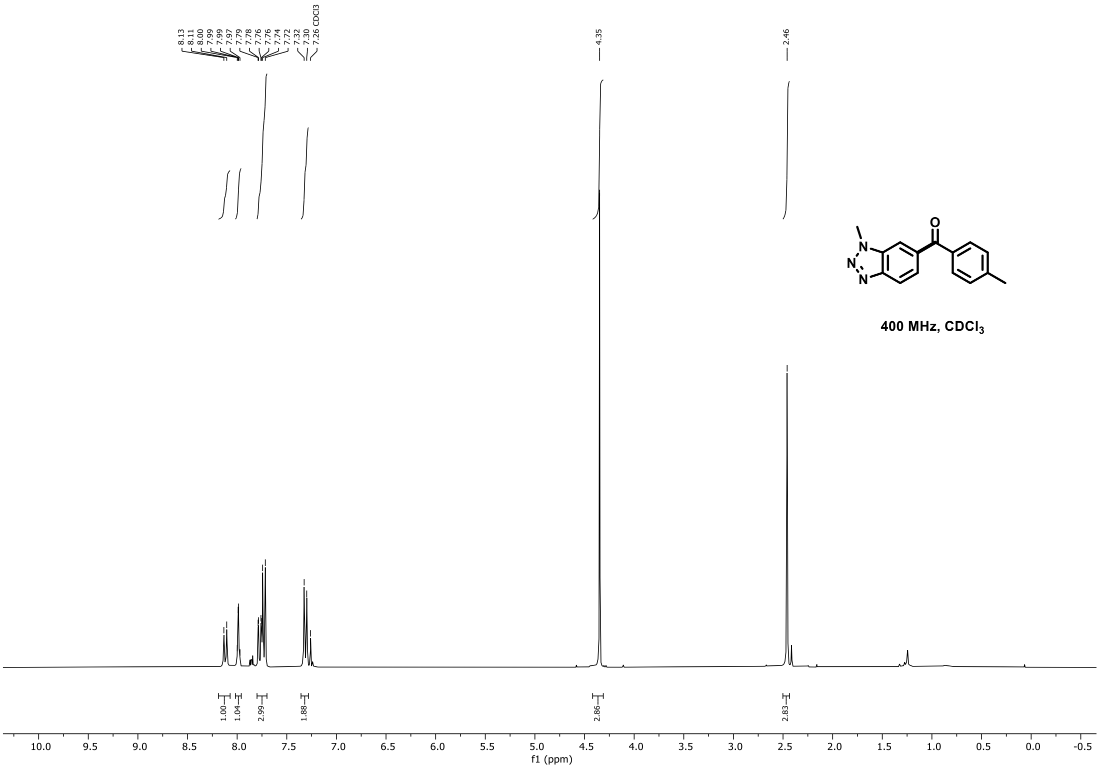

^{13}C (101 MHz, CDCl$_3$)

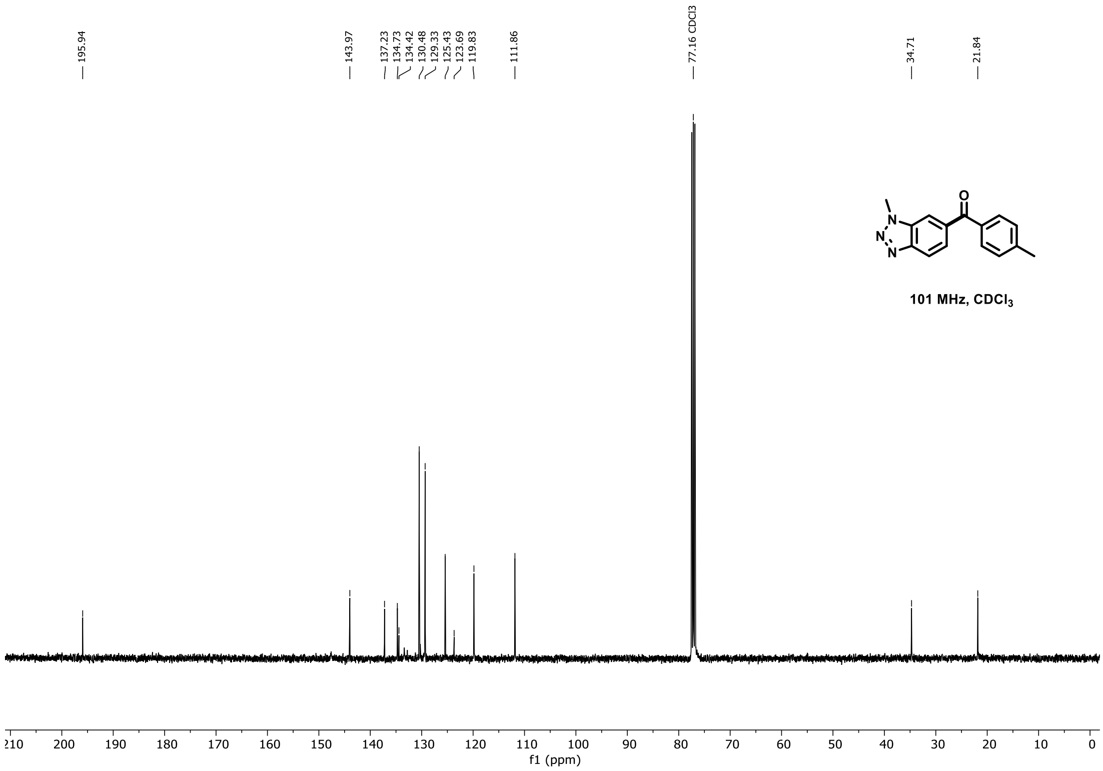

5-(4-Methylbenzoyl)isobenzofuran-1(3H)-one (13)

^{1}H (300 MHz, $CDCl_3$)

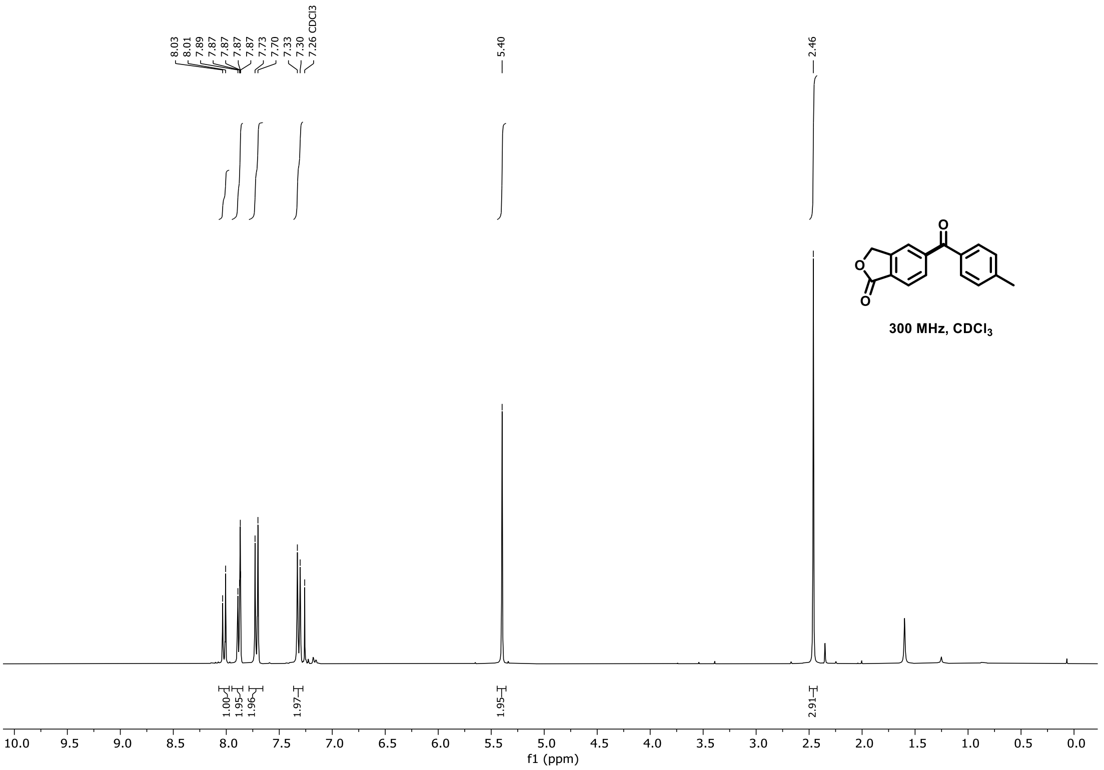

^{13}C (101 MHz, $CDCl_3$)

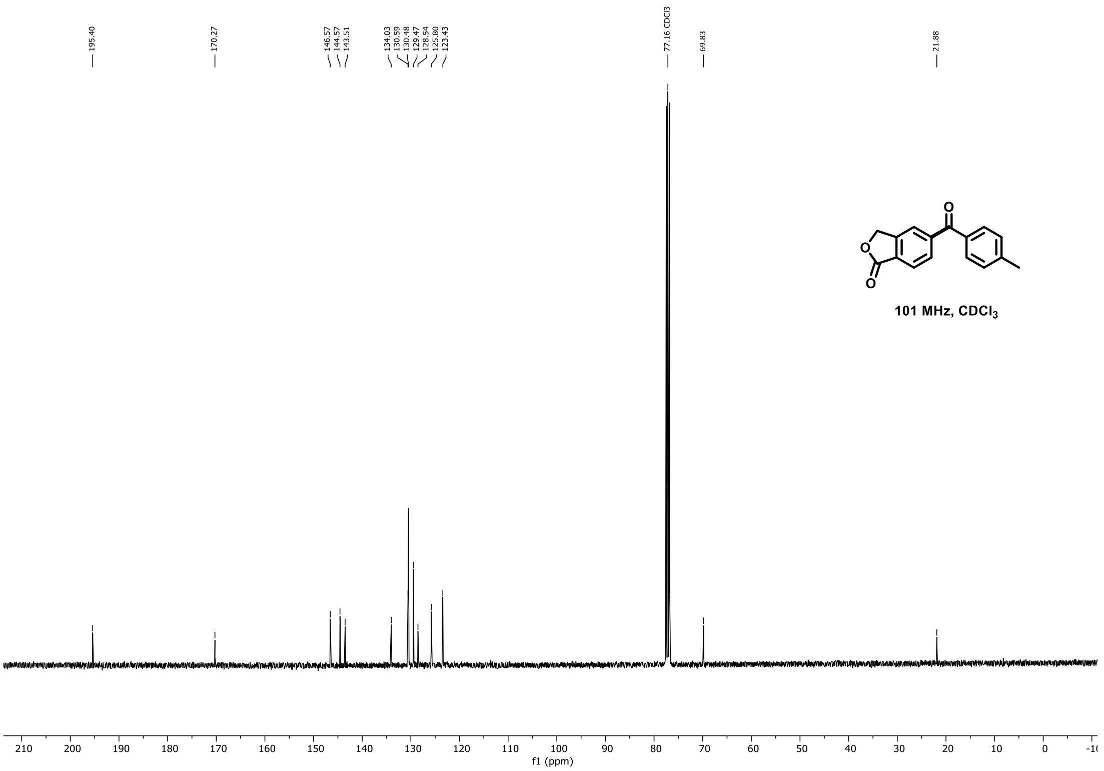

Benzo[b]thiophen-5-yl(p-tolyl)methanone (14)

^{1}H (400 MHz, $CDCl_3$)

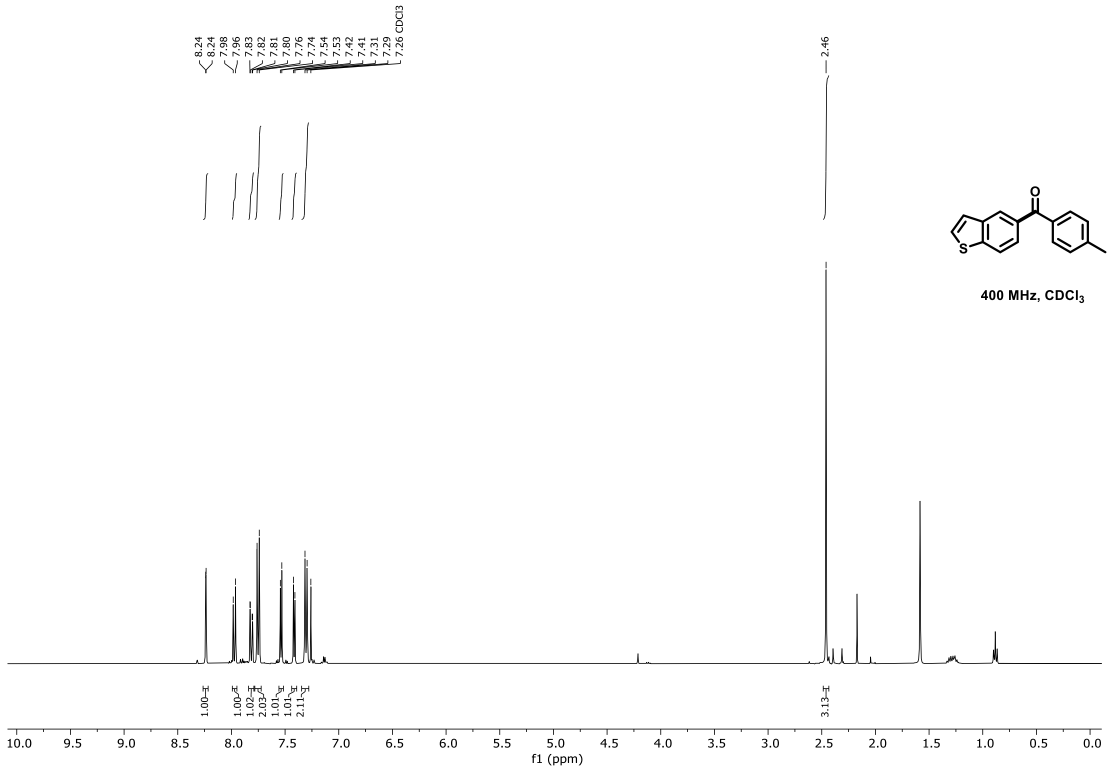

^{13}C (101 MHz, $CDCl_3$)

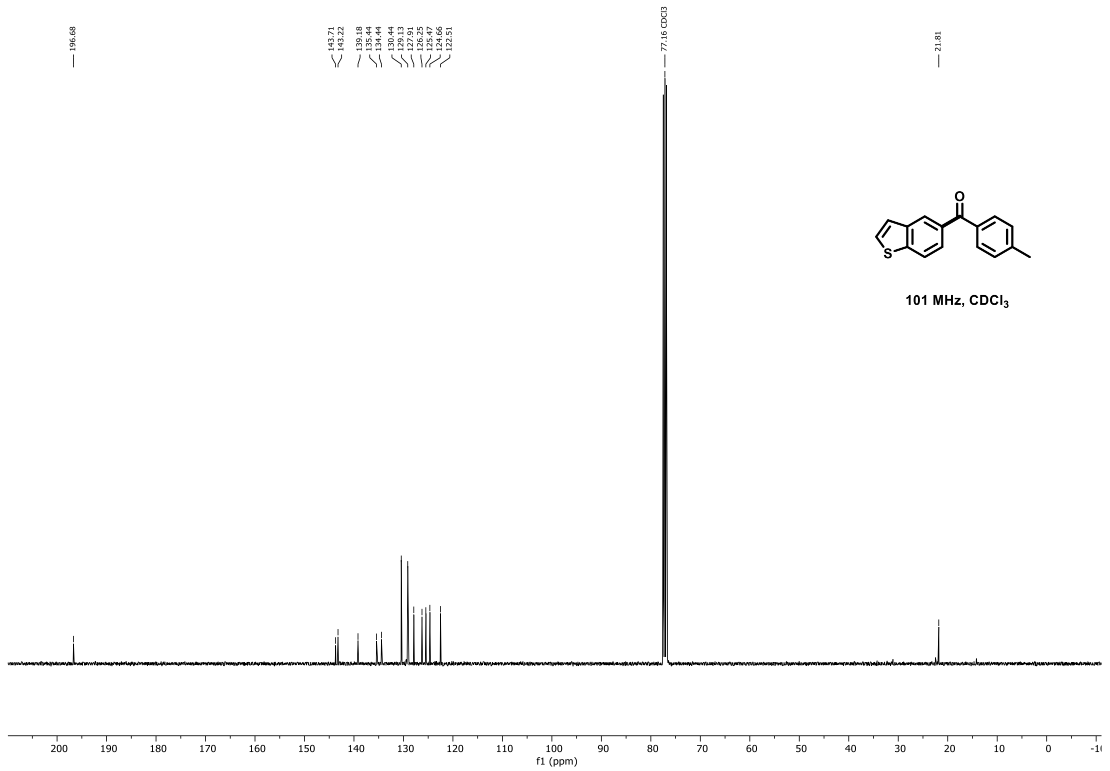

(4-Methoxyphenyl)(4-(trifluoromethyl)phenyl)methanone (15)

^{1}H (400 MHz, $CDCl_3$)

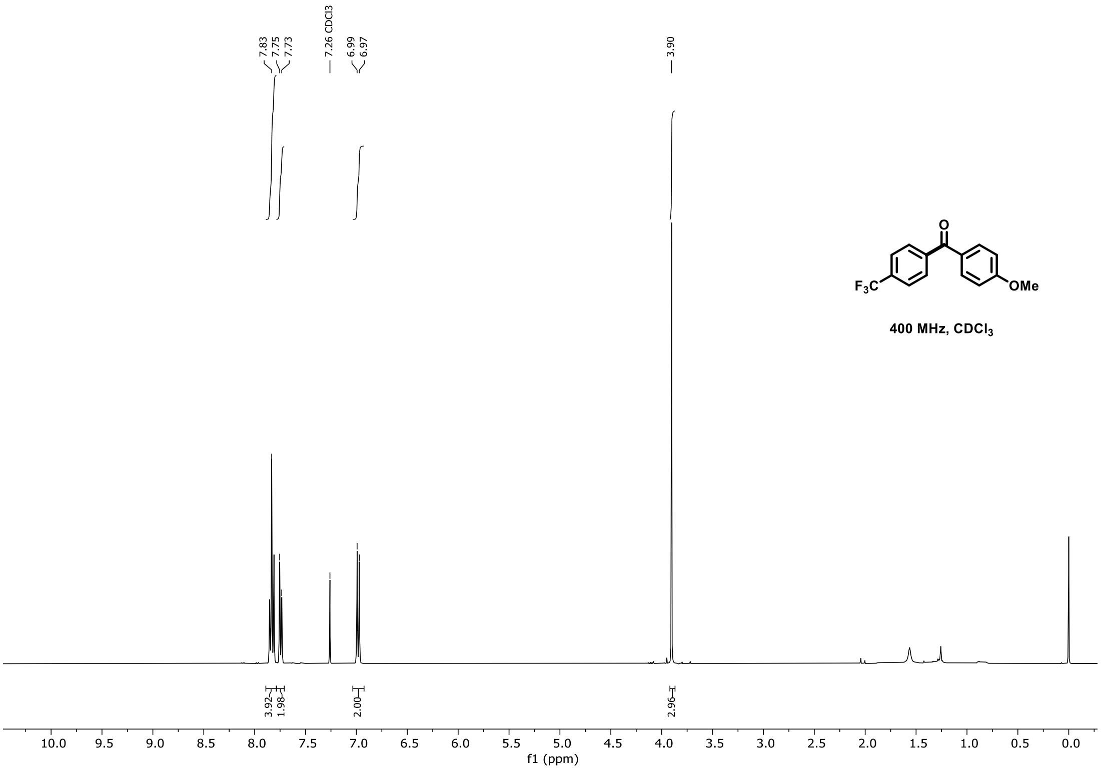

^{13}C (101 MHz, $CDCl_3$)

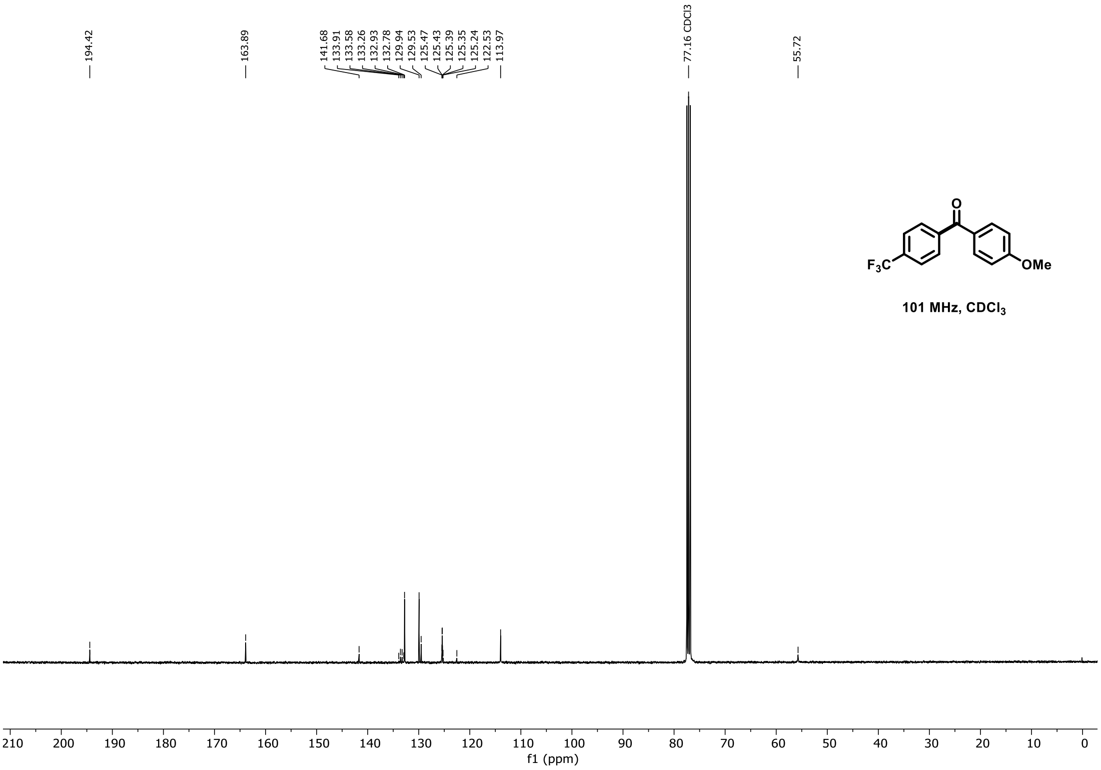

^{19}F (282 MHz, $CDCl_3$)

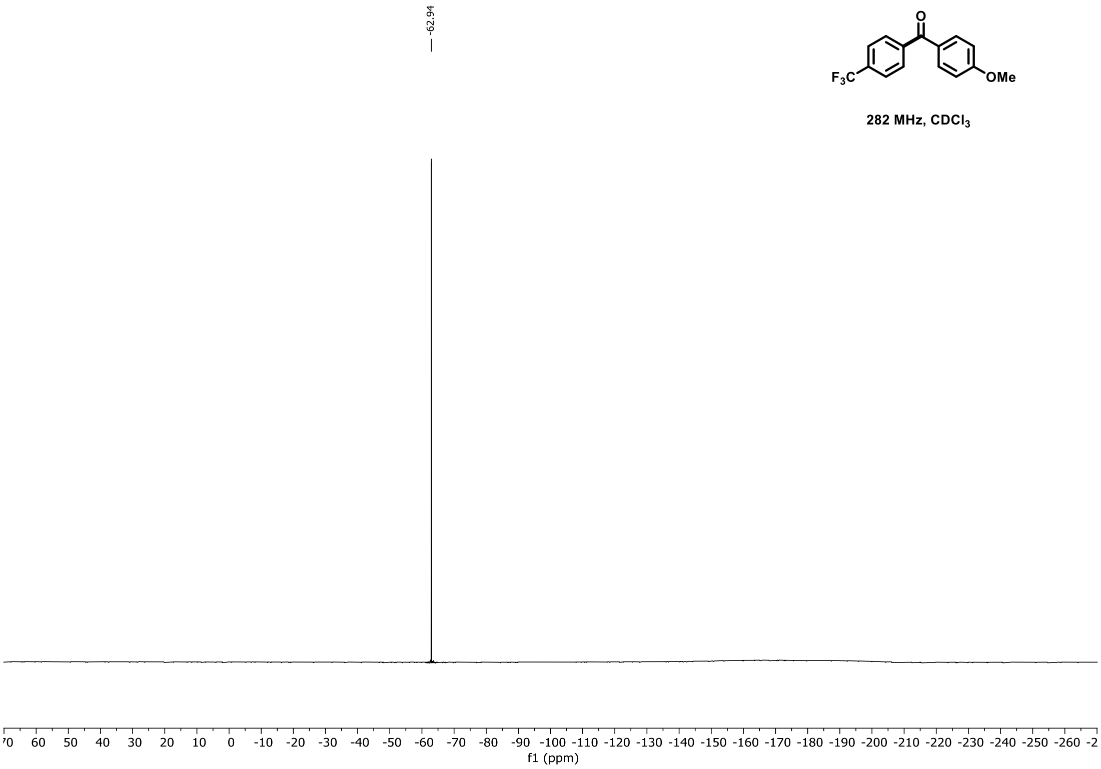

(E)-3-Phenyl-1-(p-tolyl)prop-2-en-1-one (16)

^{1}H (400 MHz, $CDCl_3$)

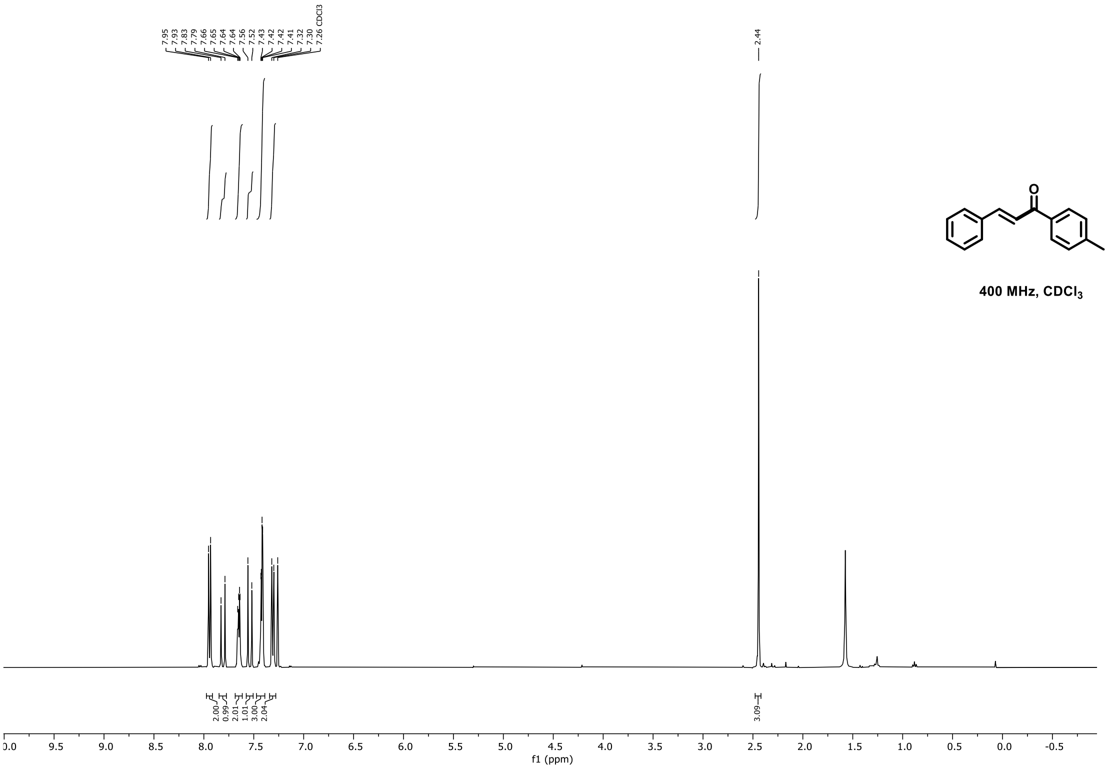

^{13}C (101 MHz, $CDCl_3$)

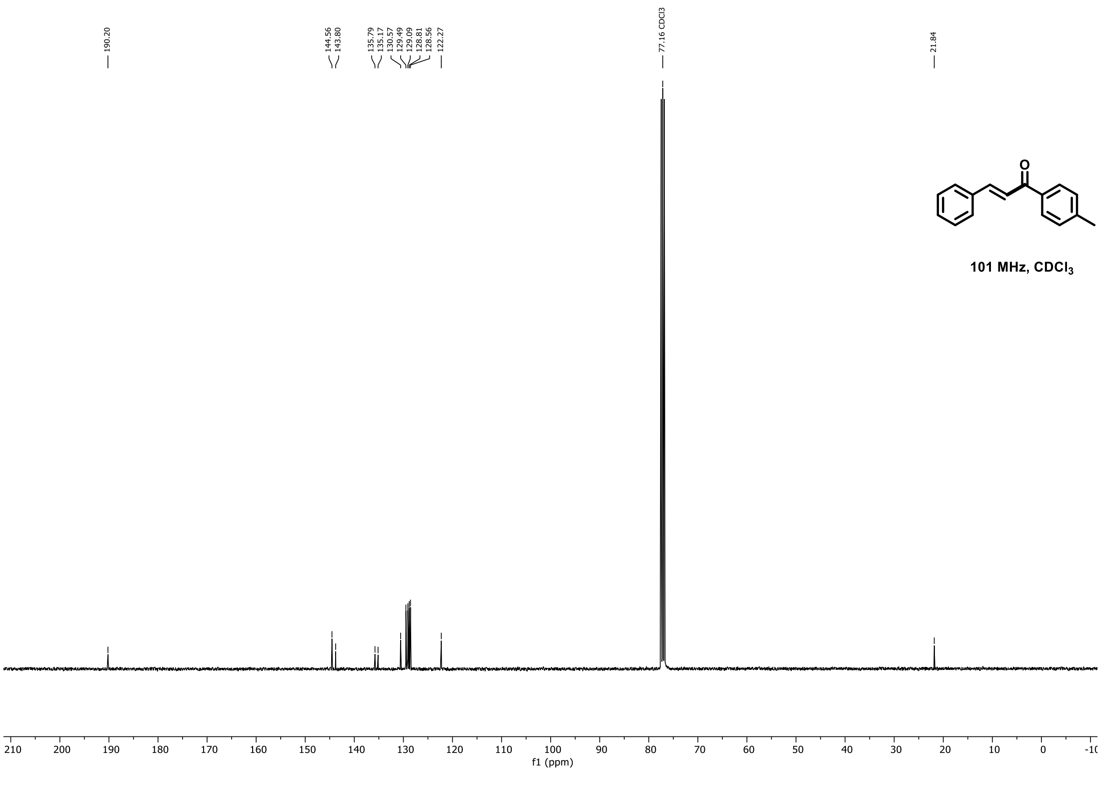

(1H-Inden-2-yl)(p-tolyl)methanone (17)

^{1}H (400 MHz, $CDCl_3$)

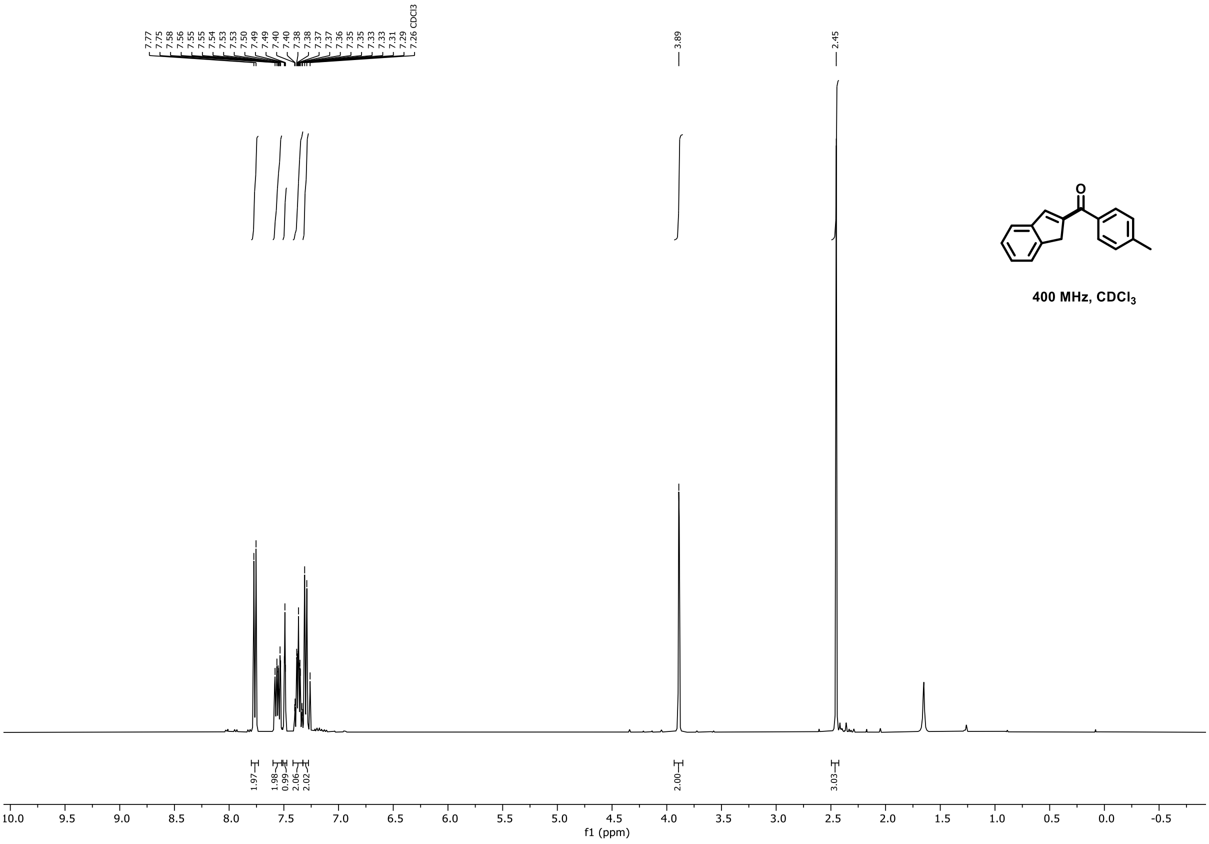

^{13}C (101 MHz, $CDCl_3$)

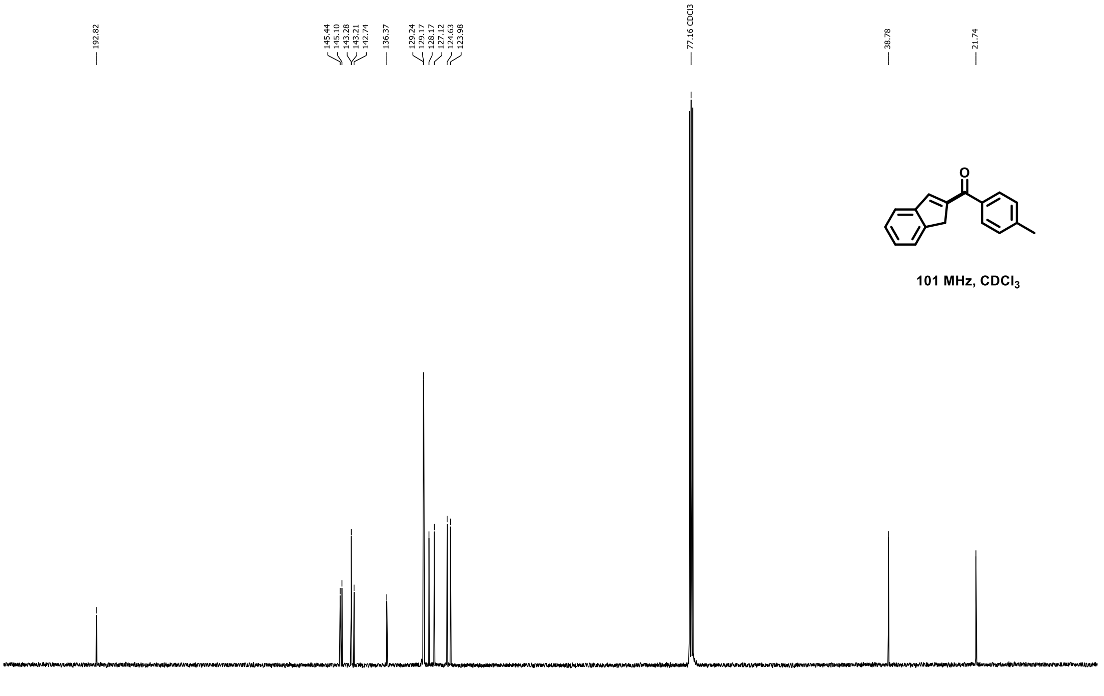

3-Methyl-1-(p-tolyl)but-2-en-1-one (18)

^{1}H (400 MHz, CDCl$_3$)

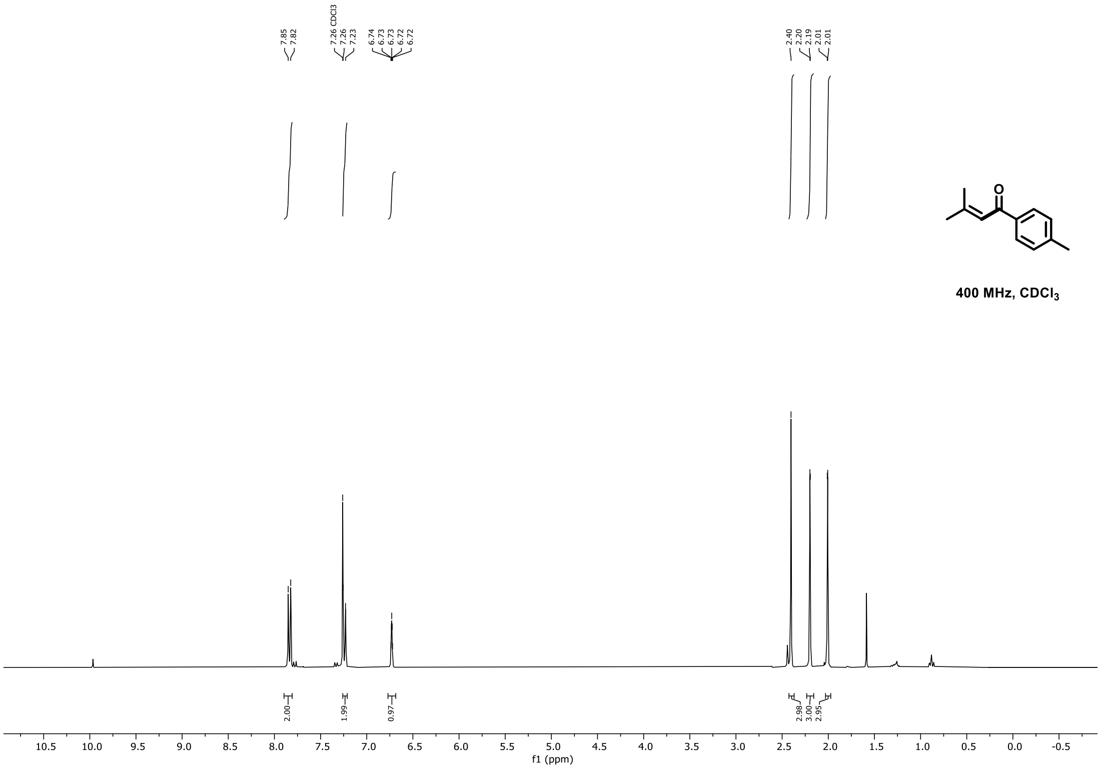

^{13}C (101 MHz, CDCl$_3$)

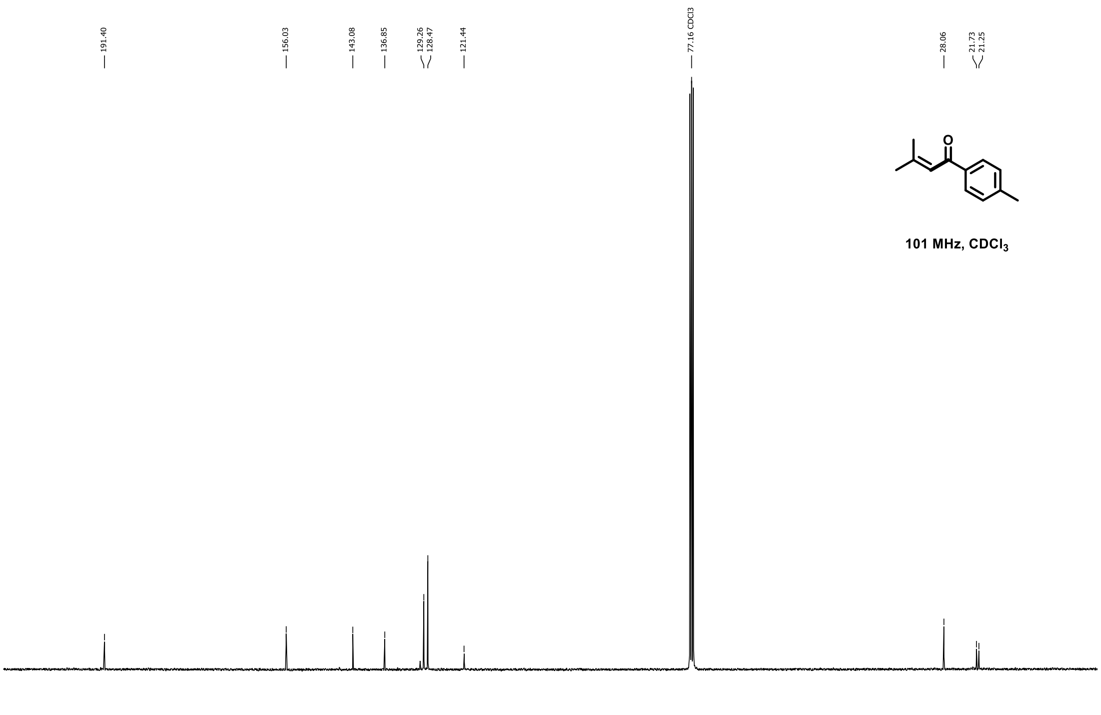

Methyl 4-(4-methylbenzoyl)benzoate (19)

^{1}H (300 MHz, $CDCl_3$)

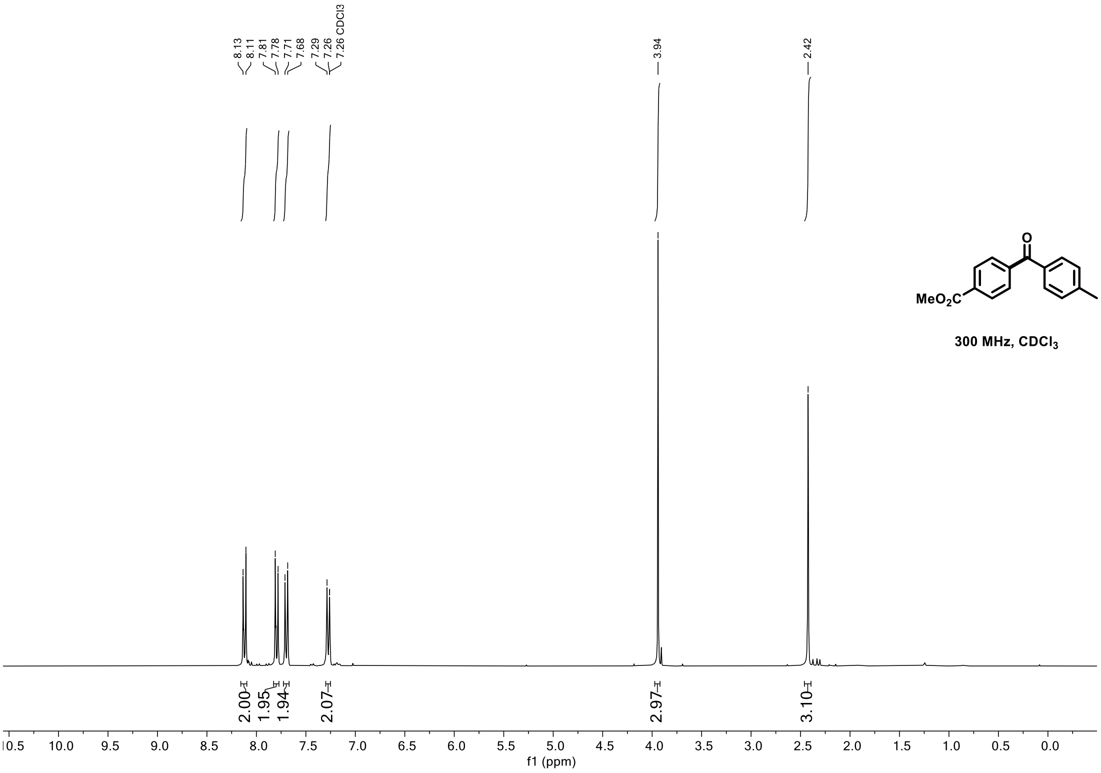

^{13}C (75 MHz, $CDCl_3$)

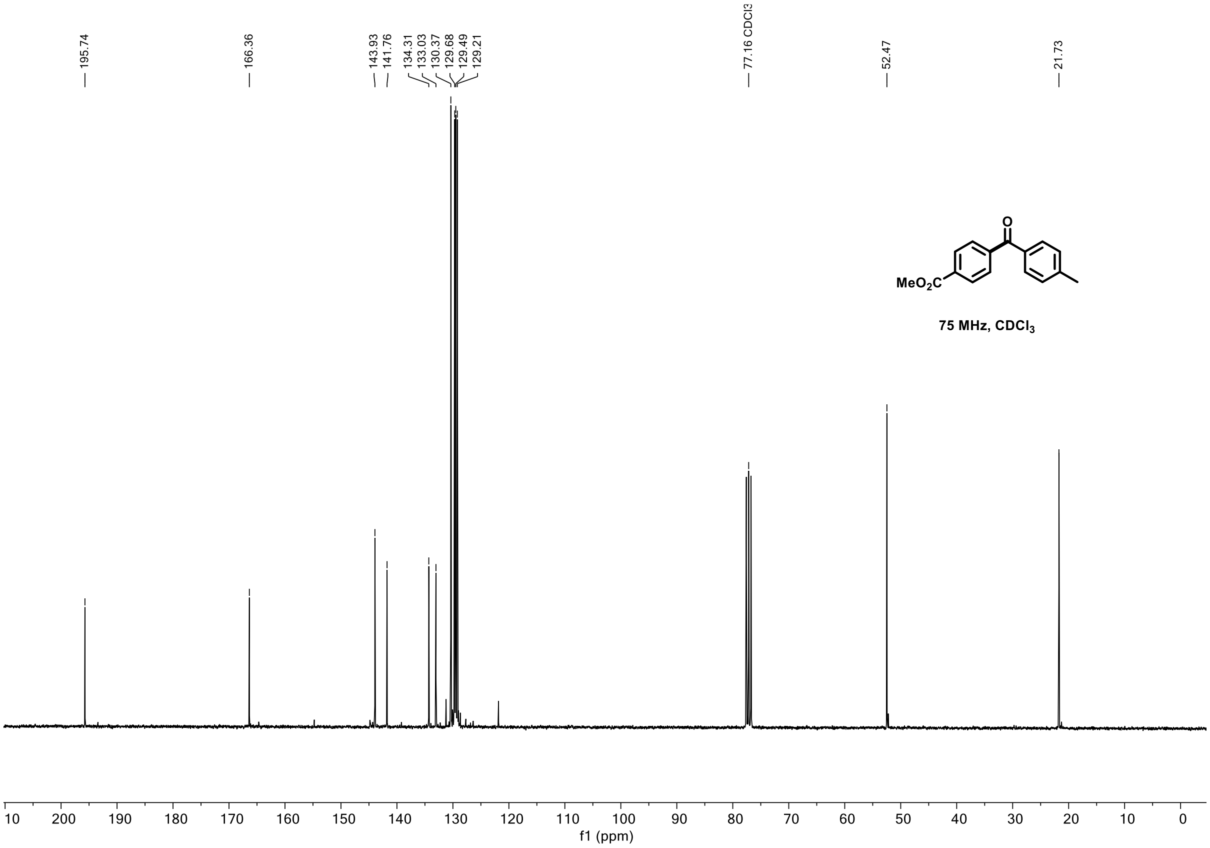

M*ethyl 4-(4-ethylbenzoyl)benzoate (20)*

^{1}H (400 MHz, $CDCl_3$)

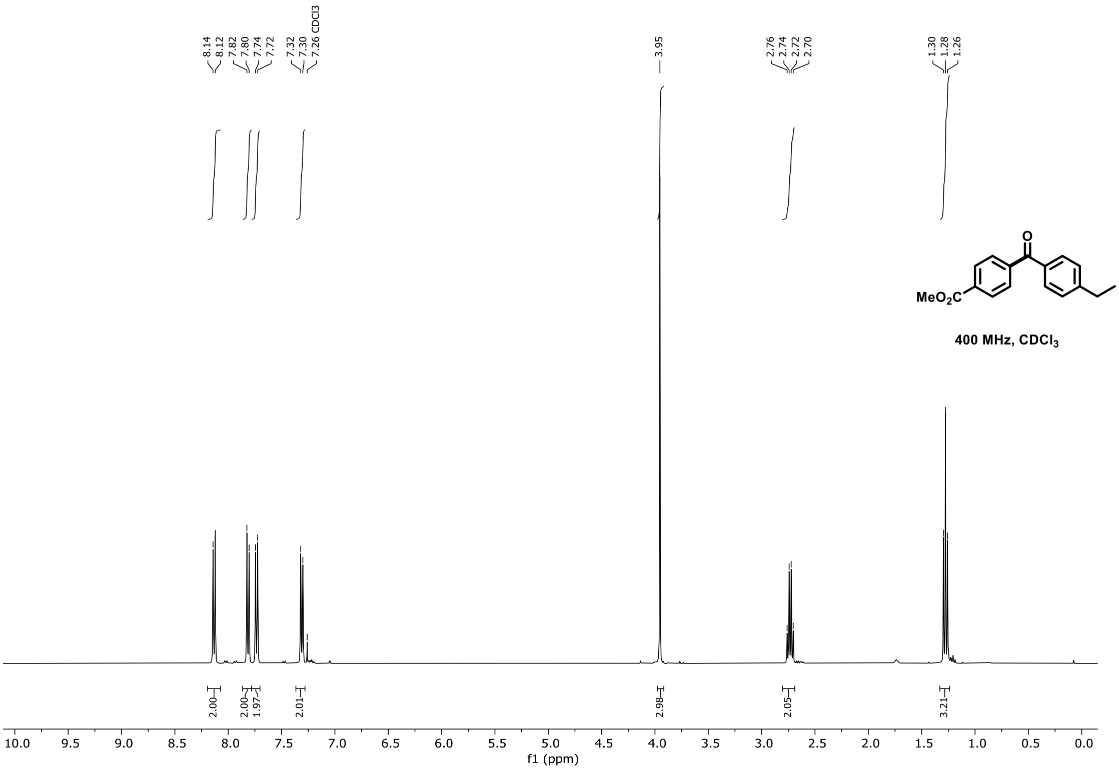

^{13}C (101 MHz, $CDCl_3$)

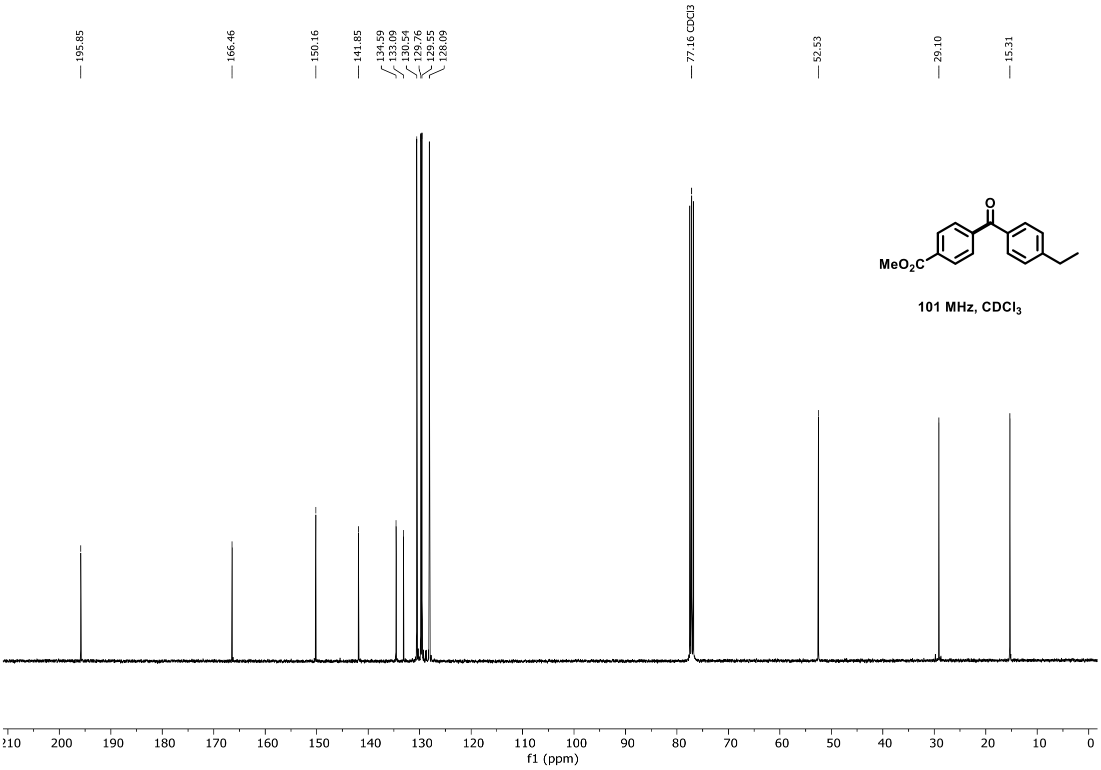

Methyl 4-(4-methoxybenzoyl)benzoate (21)

^{1}H (400 MHz, $CDCl_3$)

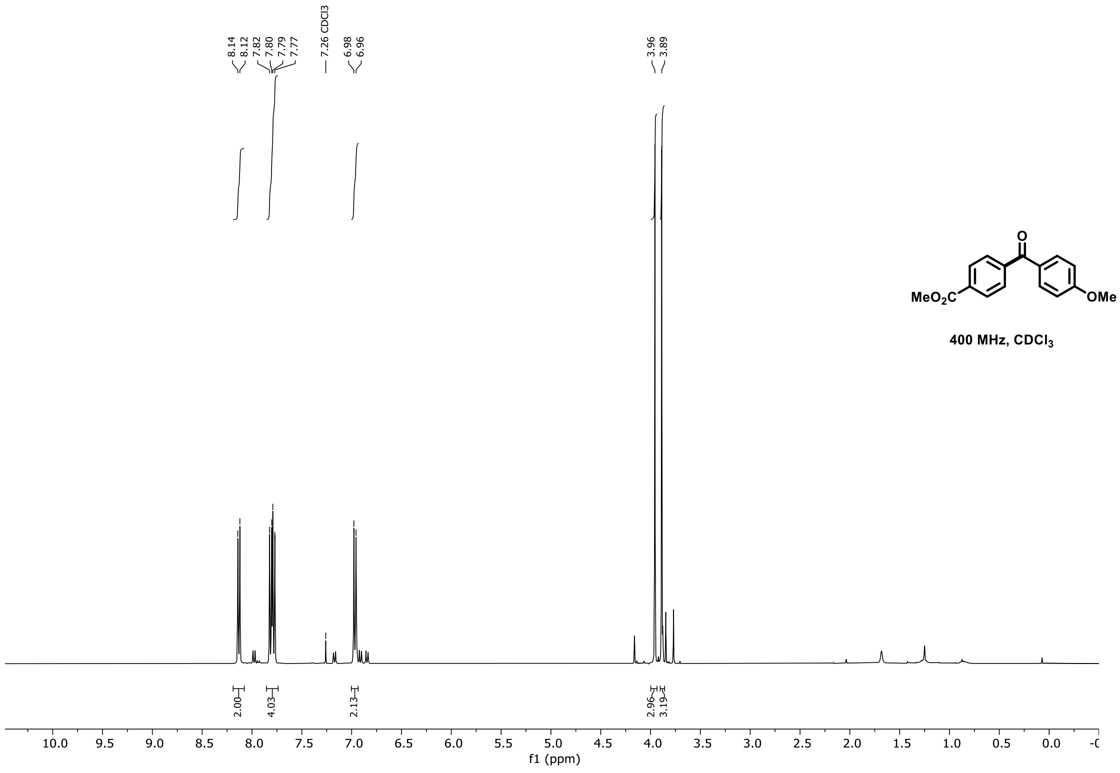

^{13}C (101 MHz, $CDCl_3$)

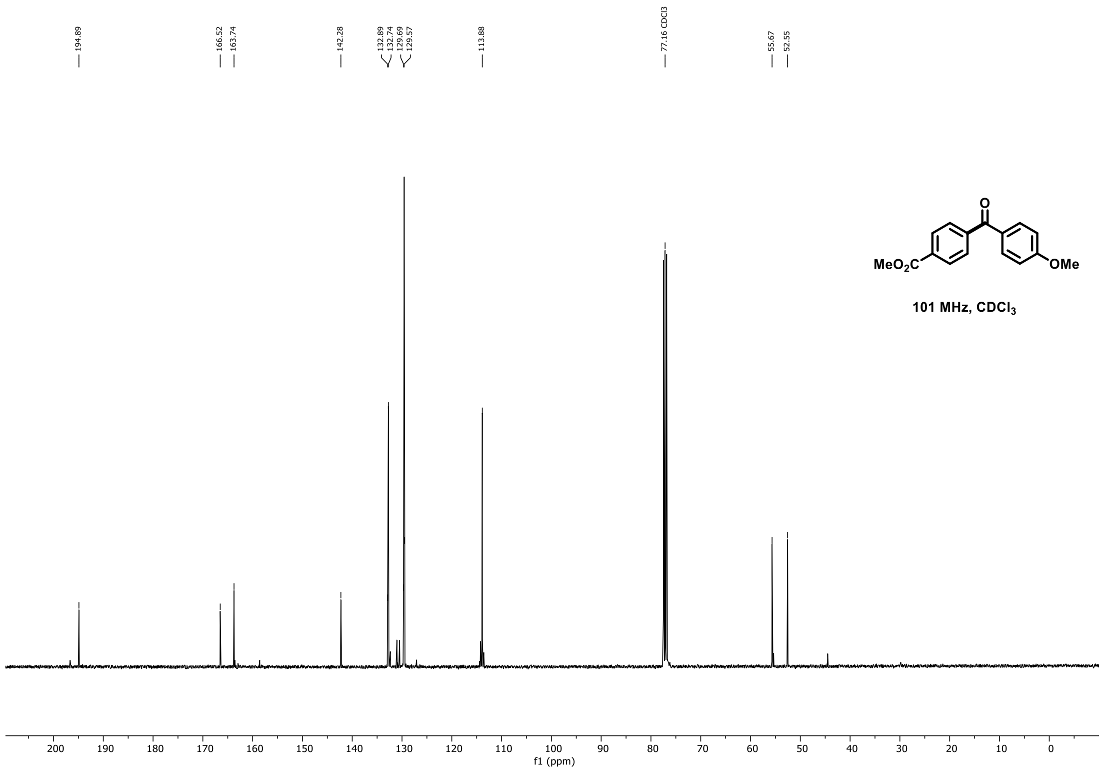

Methyl 4-(4-fluorobenzoyl)benzoate (22)

^{1}H (300 MHz, $CDCl_3$)

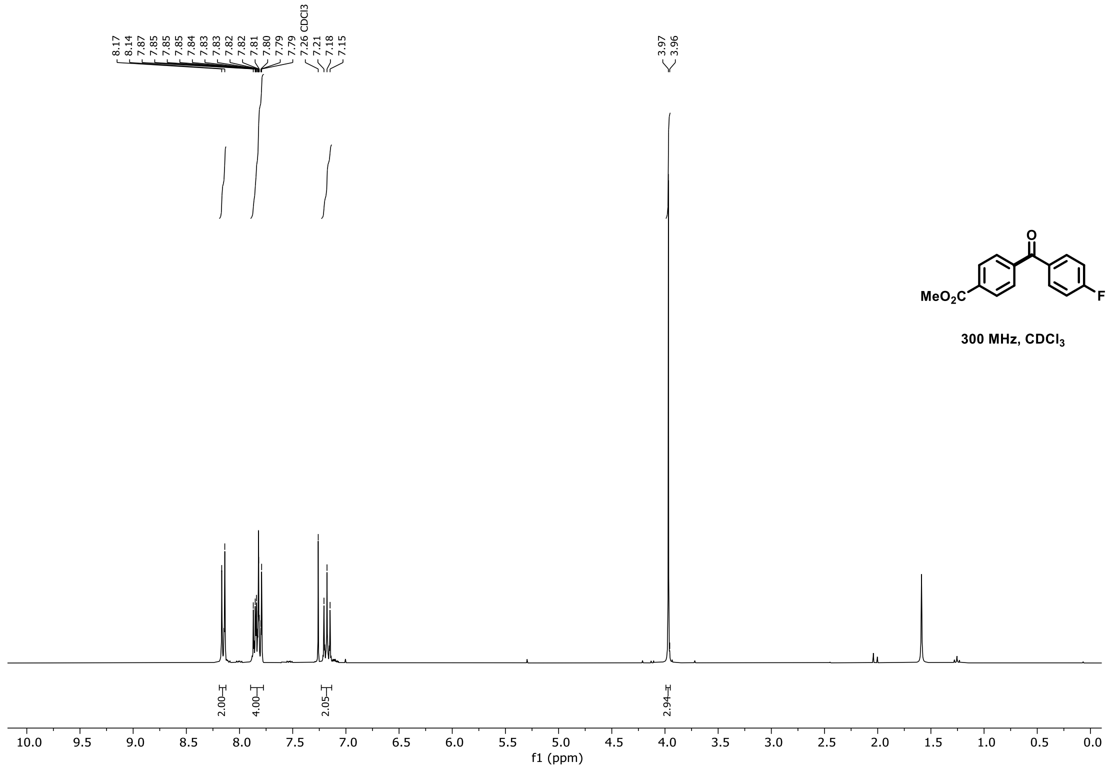

^{13}C (75 MHz, $CDCl_3$)

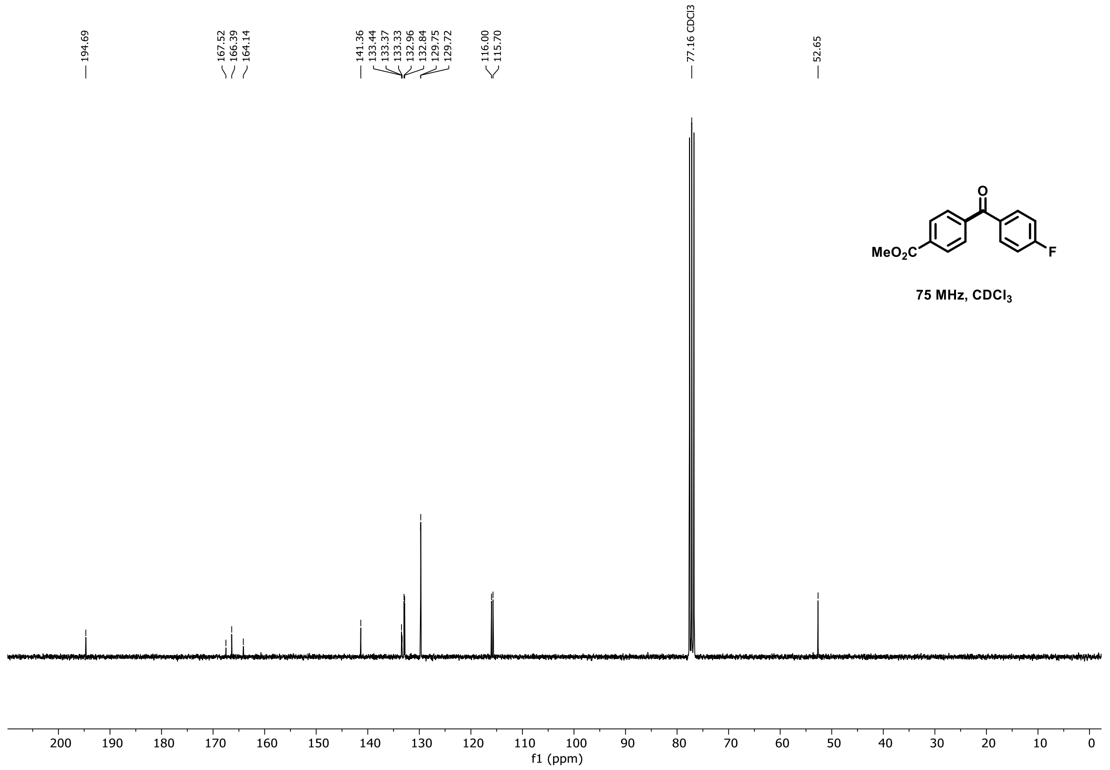

^{19}F (282 MHz, $CDCl_3$)

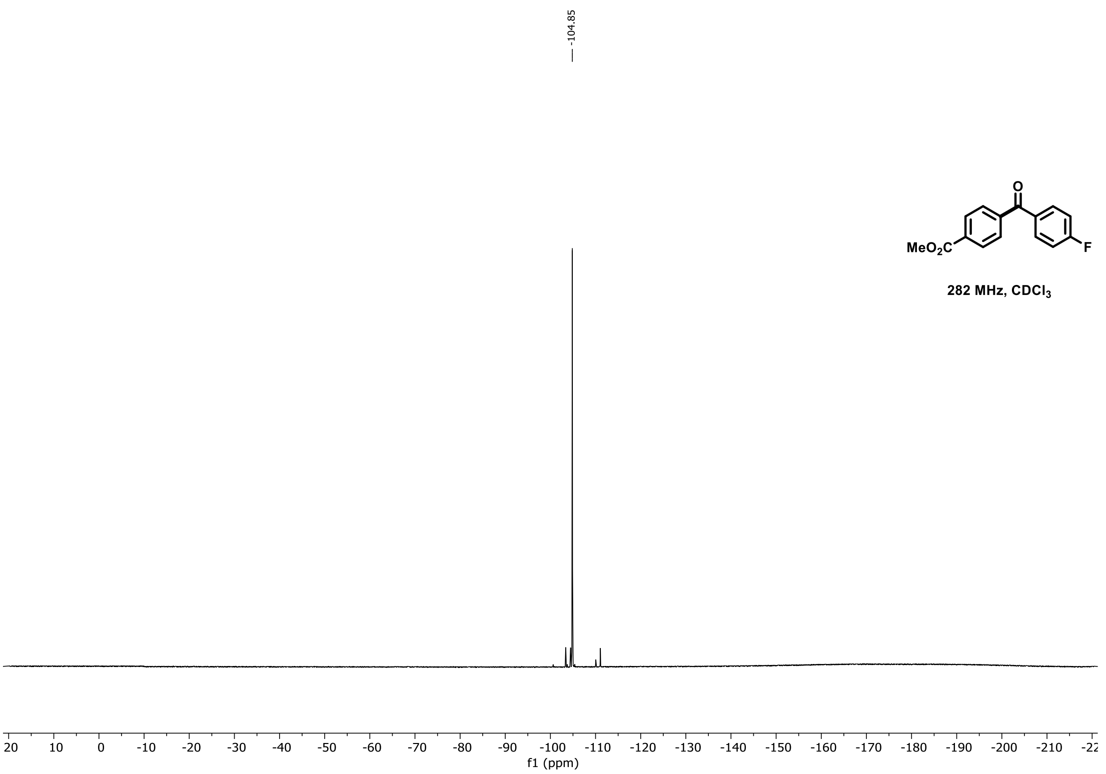

Methyl 4-propionylbenzoate (23)

^{1}H (400 MHz, $CDCl_3$)

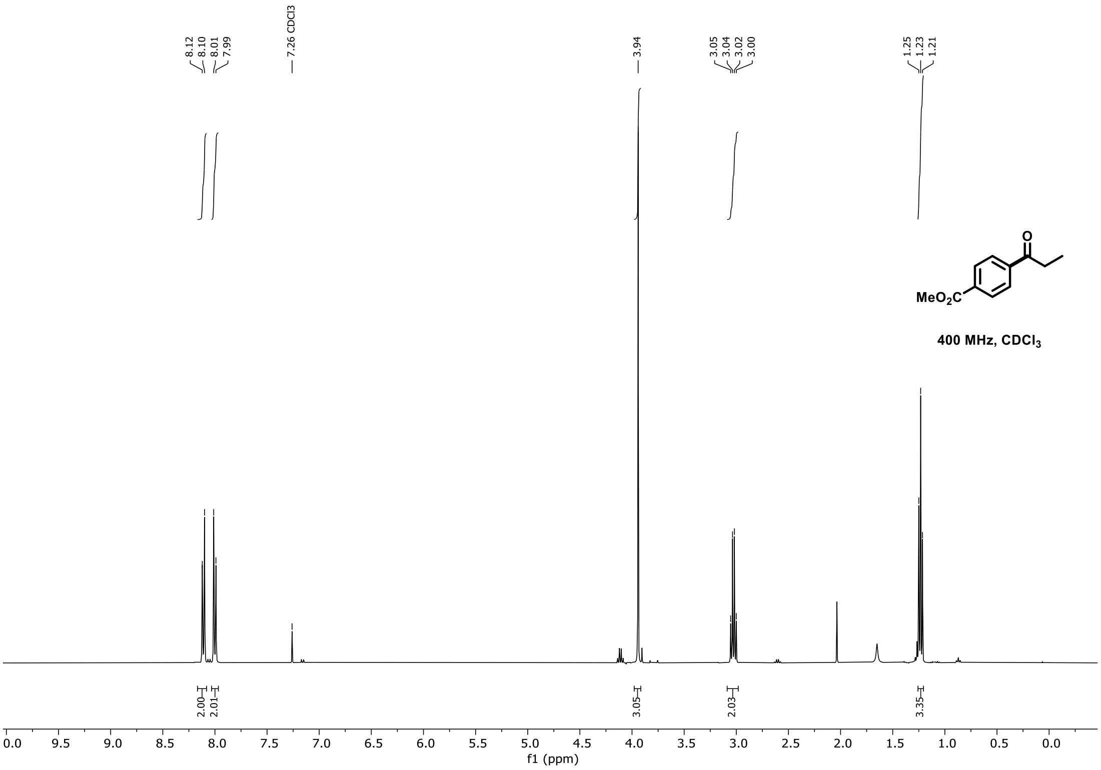

^{13}C (101 MHz, $CDCl_3$)

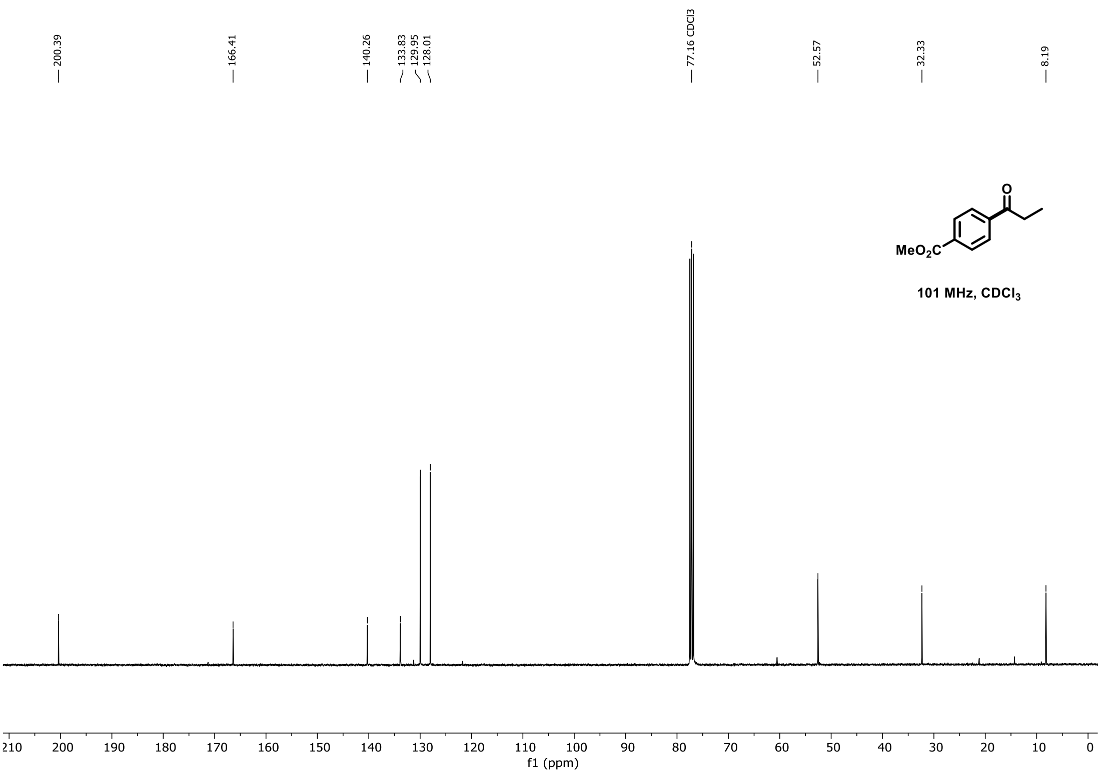

Methyl 4-isobutyrylbenzoate (24)

^{1}H (400 MHz, $CDCl_3$)

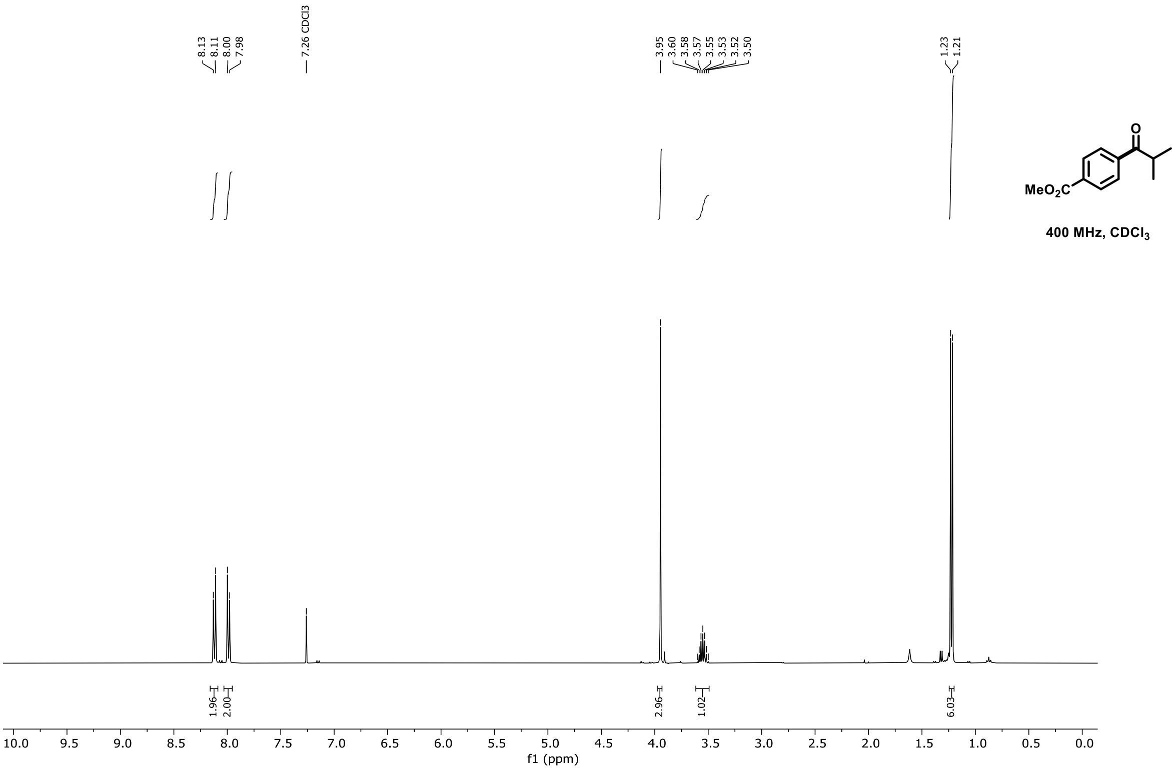

^{13}C (101 MHz, $CDCl_3$)

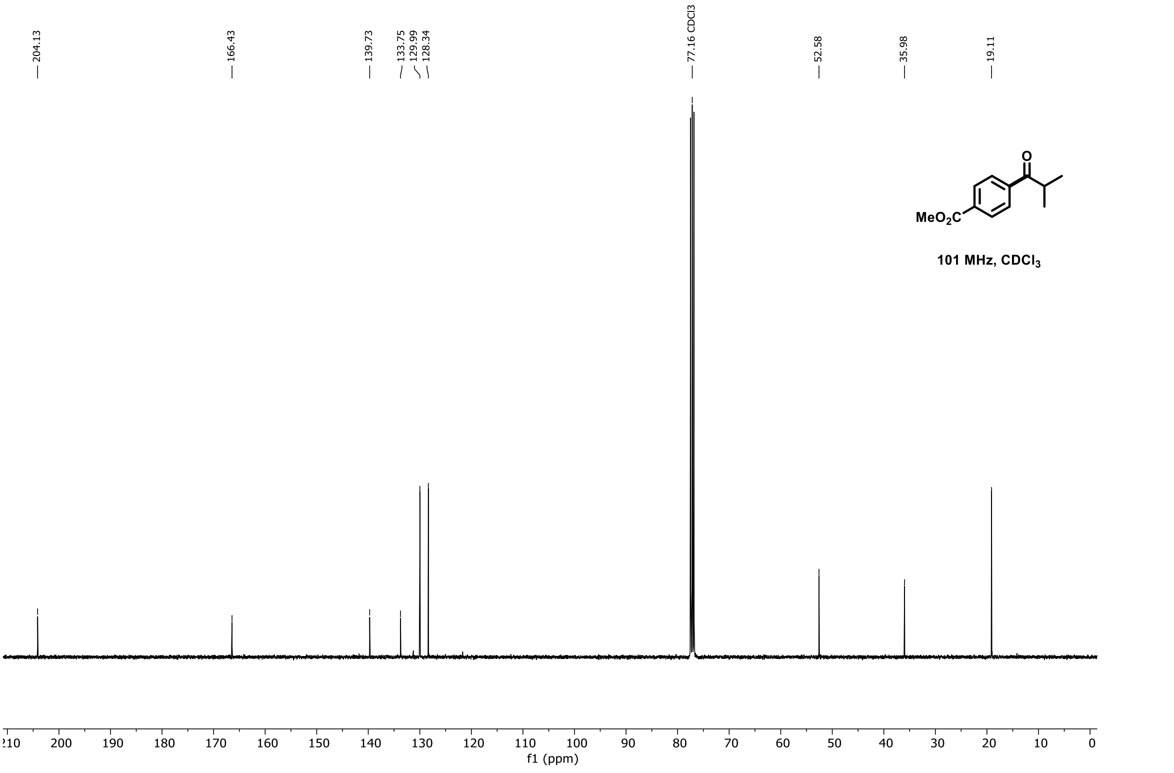

Methyl 4-(3-phenylpropanoyl)benzoate (25)

^{1}H (300 MHz, $CDCl_3$)

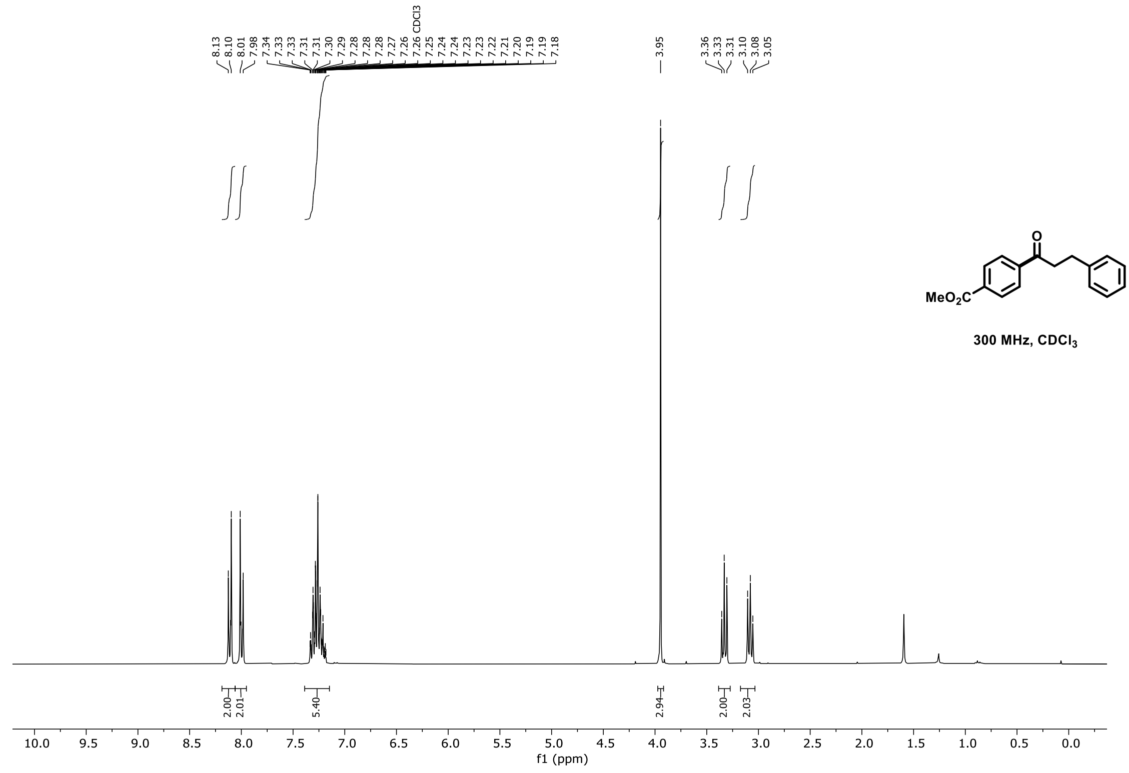

^{13}C (101 MHz, $CDCl_3$)

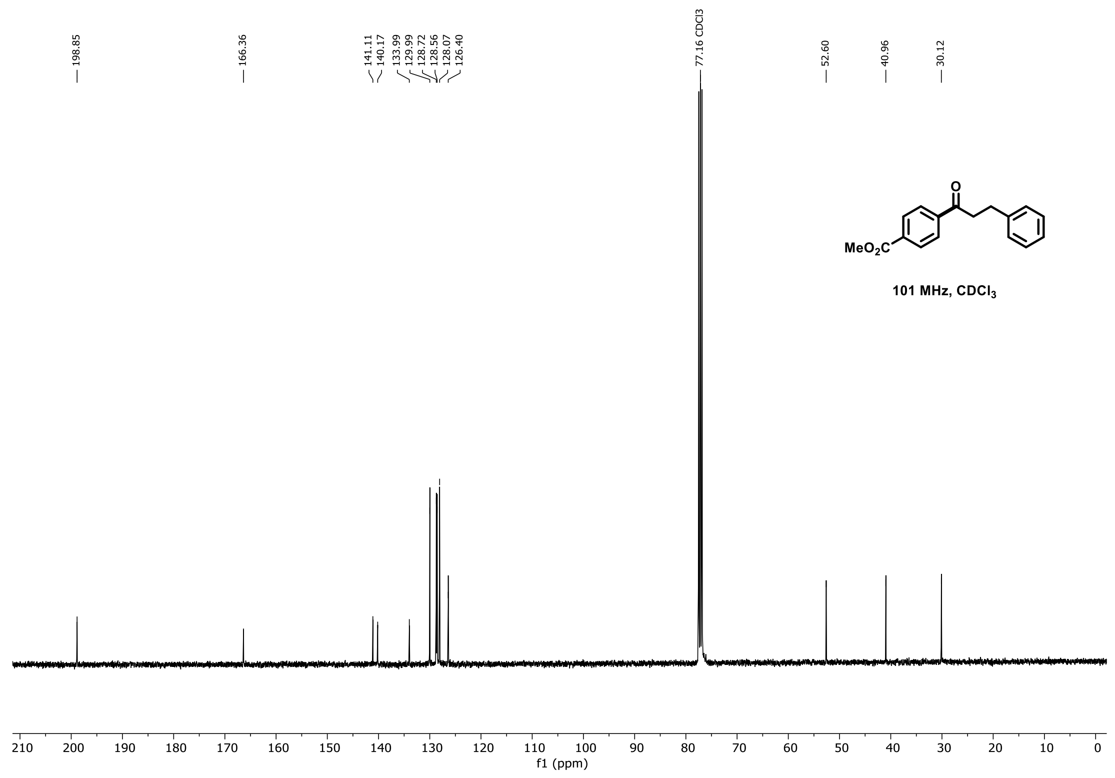

Methyl 4-(3-methylbutanoyl)benzoate (26)

^{1}H (400 MHz, $CDCl_3$)

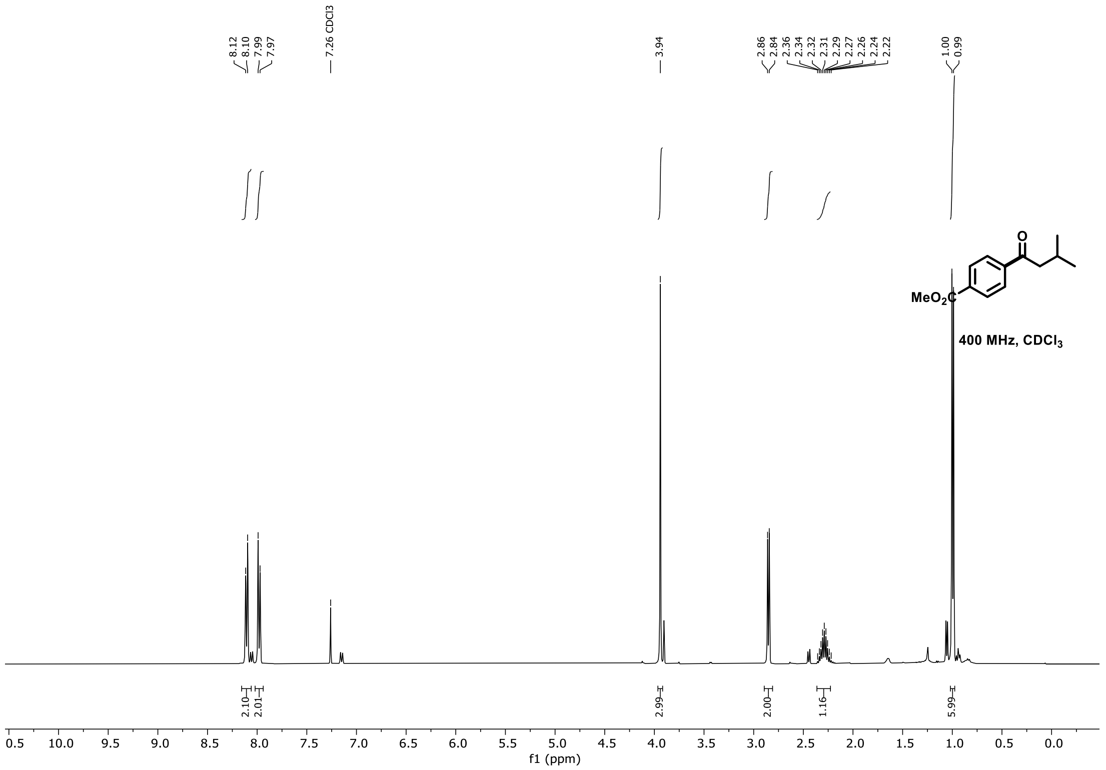

^{13}C (101 MHz, $CDCl_3$)

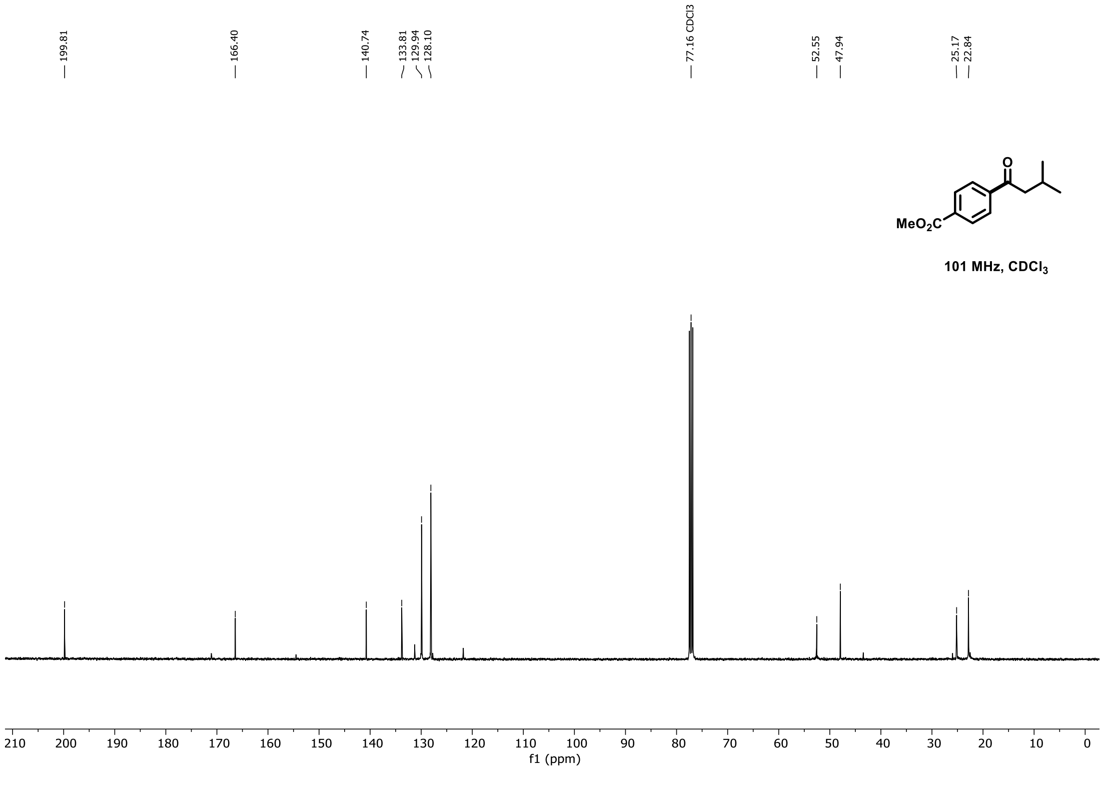

Methyl 4-heptanoylbenzoate (27)

^{1}H (300 MHz, $CDCl_3$)

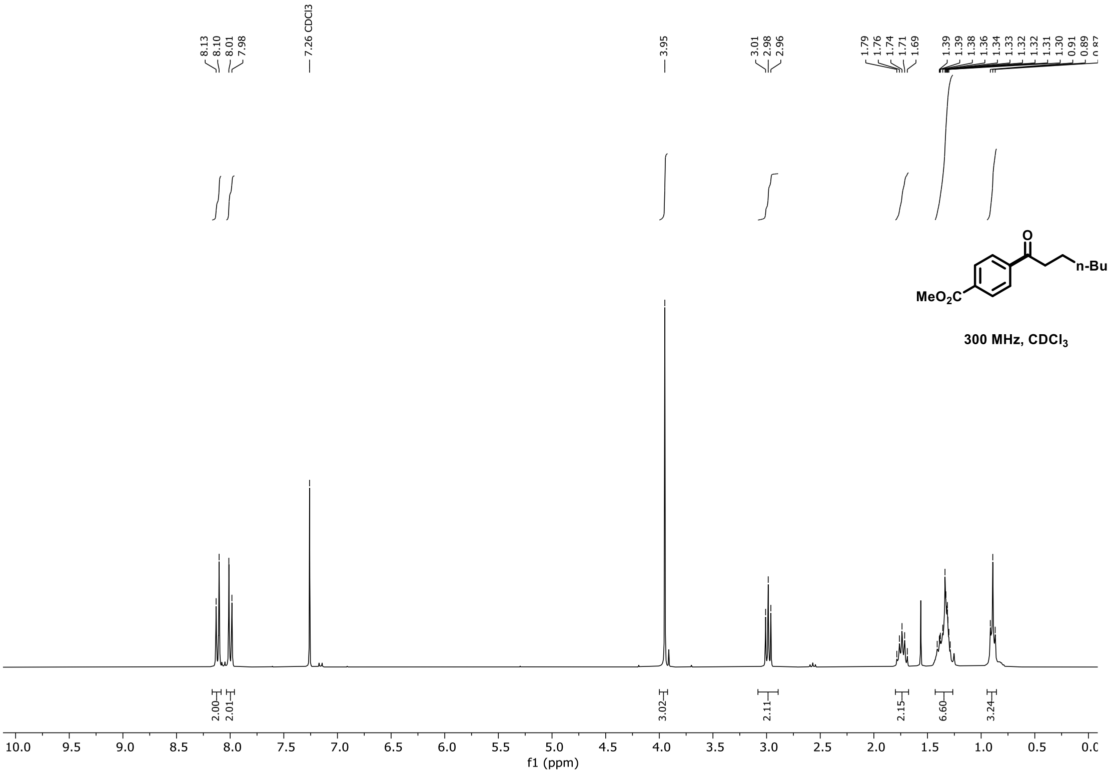

^{13}C (75 MHz, $CDCl_3$)

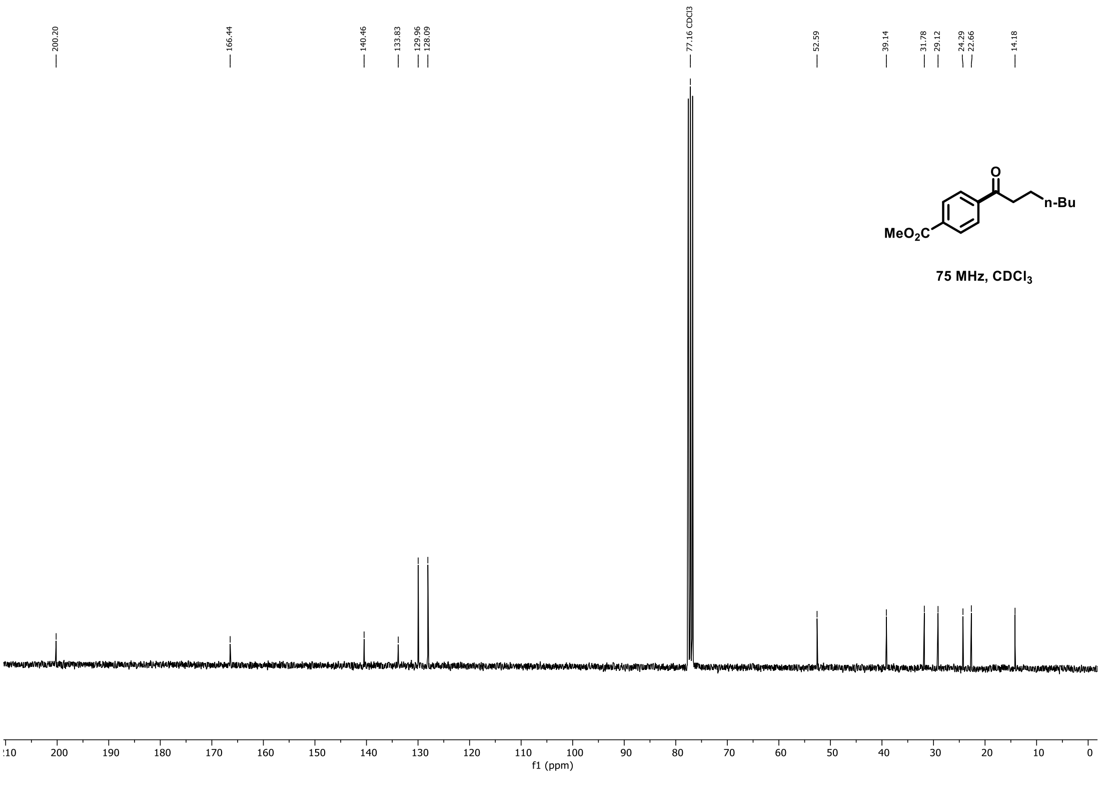

Methyl 4-(3,3-dimethylbutanoyl)benzoate (28)

^{1}H (300 MHz, $CDCl_3$)

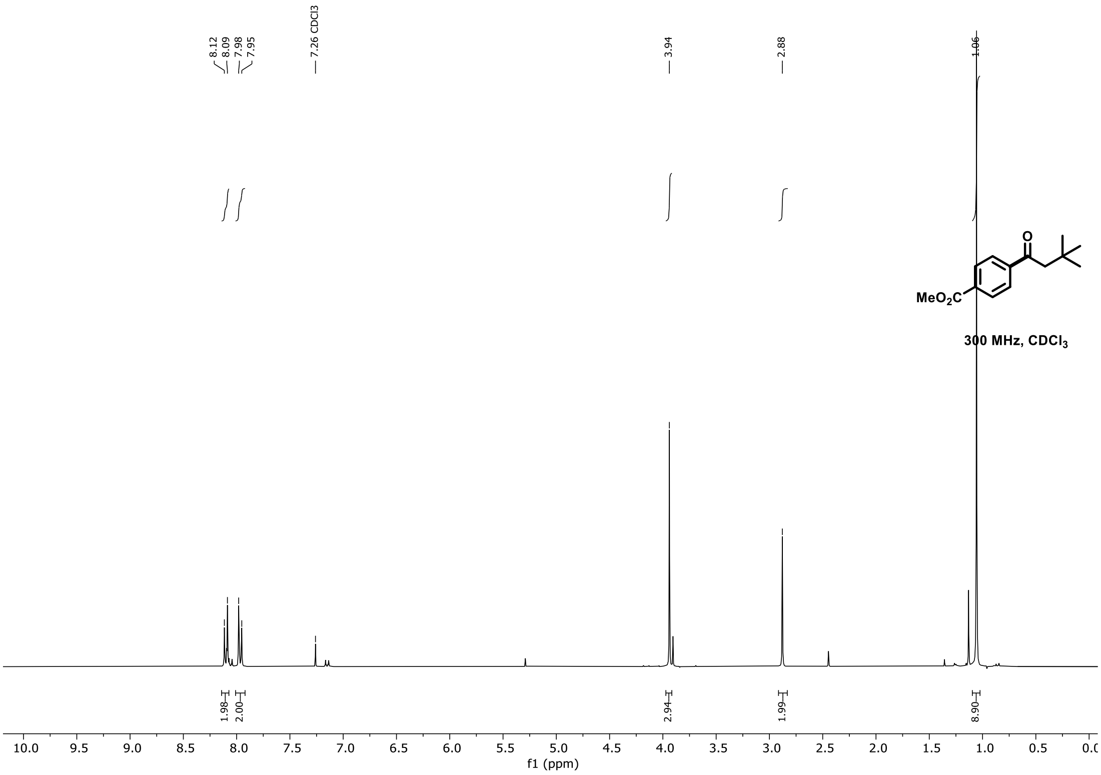

^{13}C (101 MHz, $CDCl_3$)

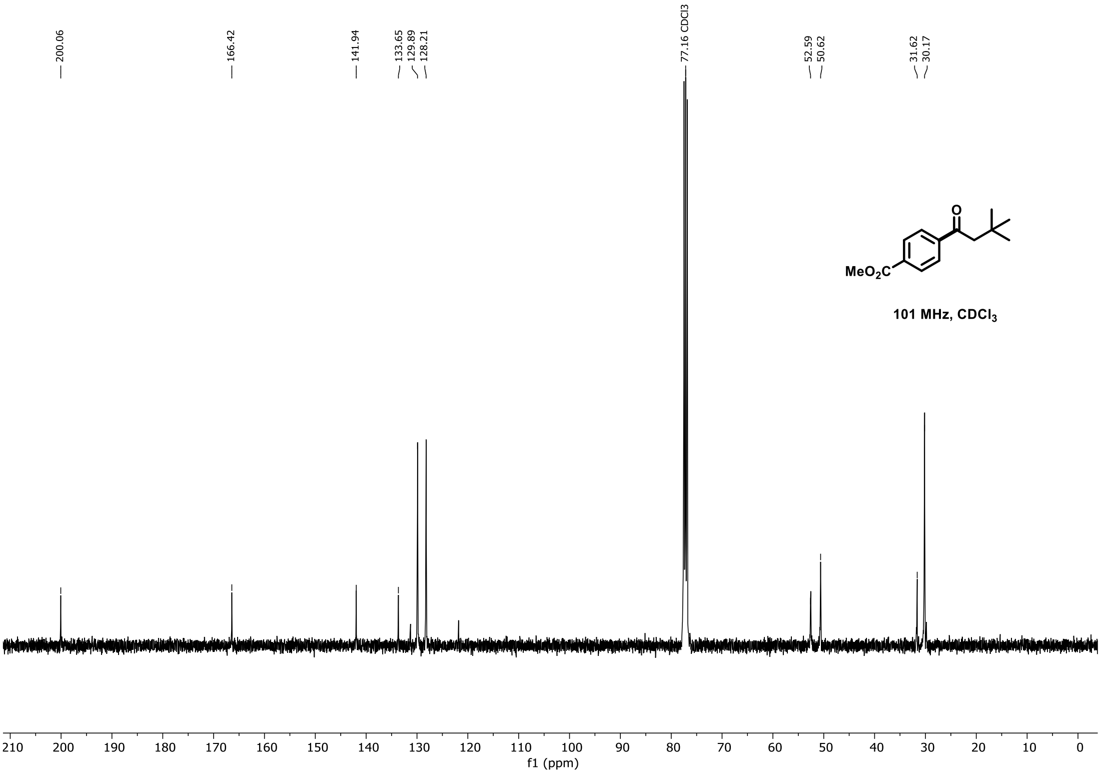

Methyl 4-(dimethylcarbamoyl)benzoate (29)

^{1}H (300 MHz, $CDCl_3$)

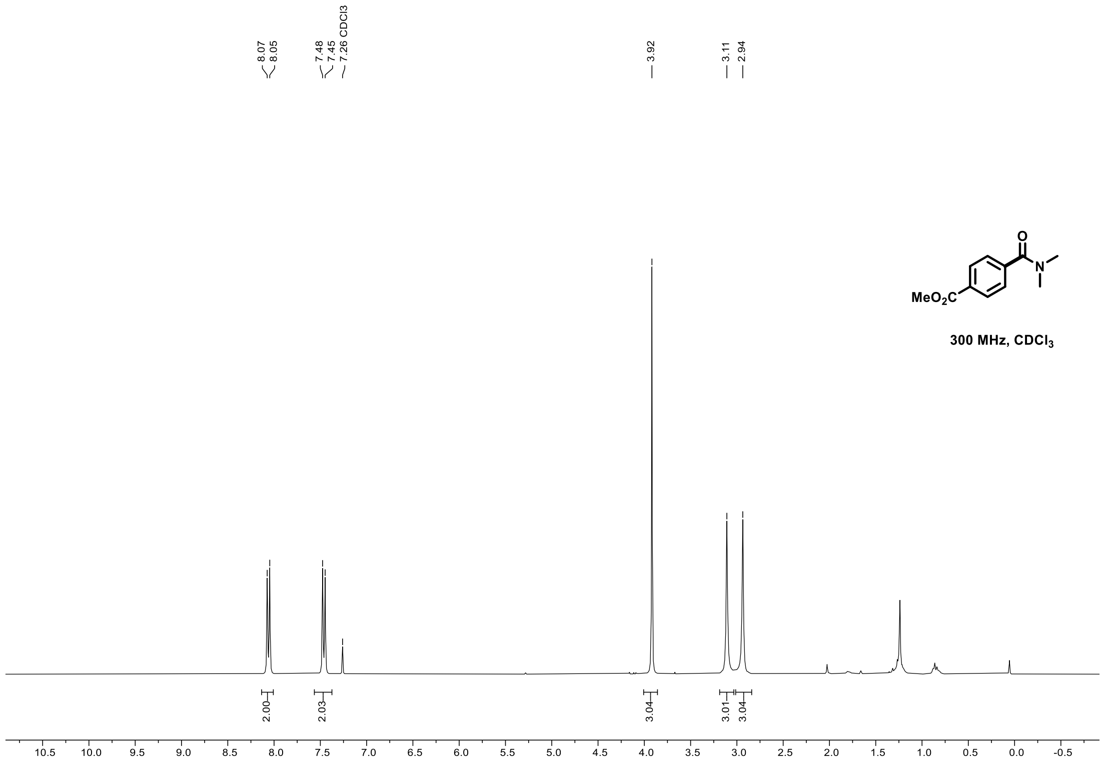

^{13}C (75 MHz, $CDCl_3$)

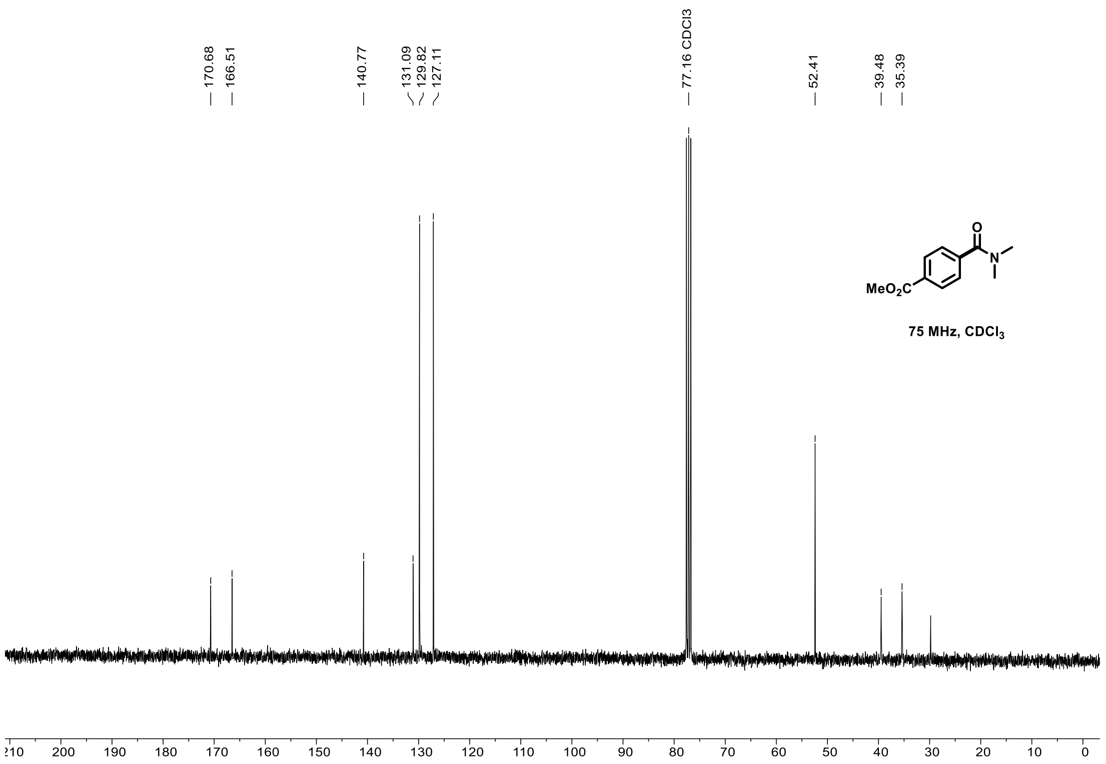

Methyl 4-(piperidine-1-carbonyl)benzoate (30)

^{1}H (400 MHz, $CDCl_3$)

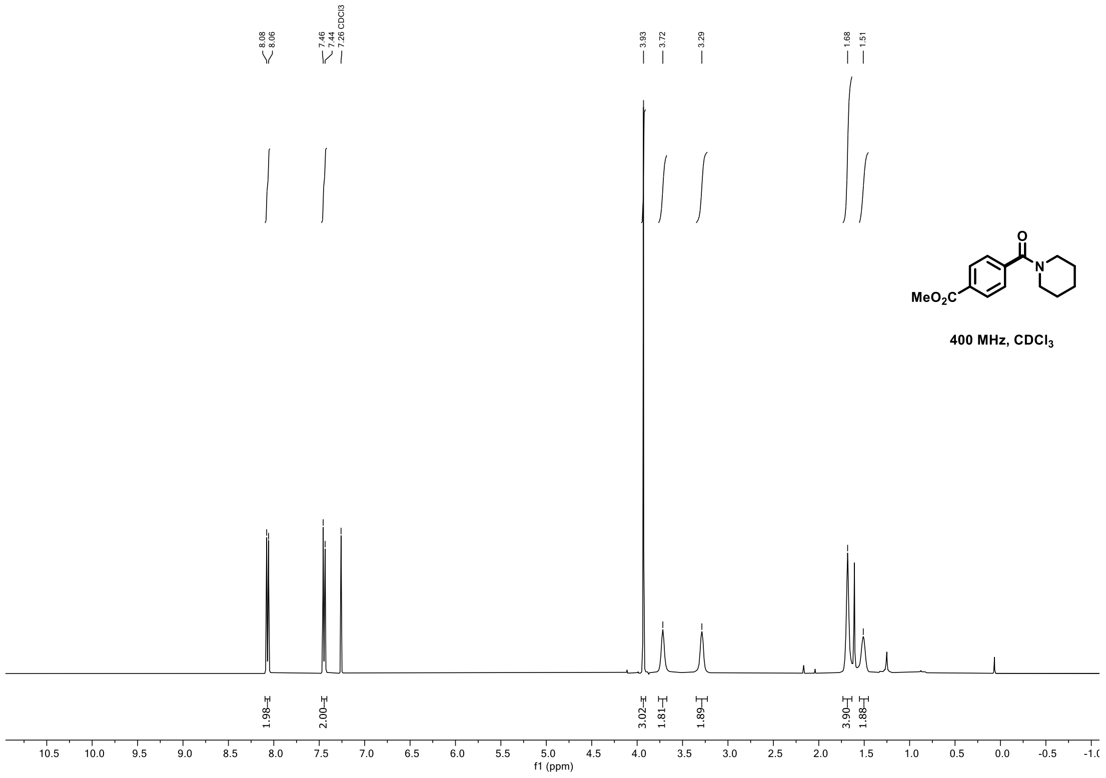

^{13}C (101 MHz, $CDCl_3$)

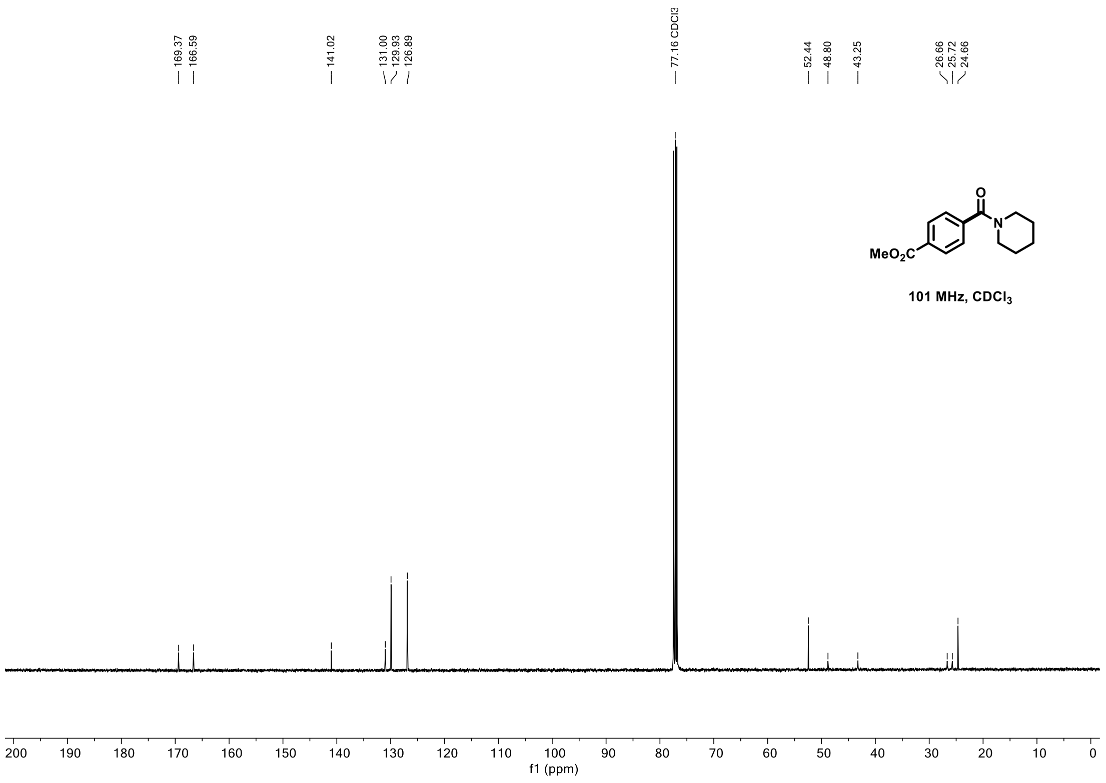

Methyl 4-(azepane-1-carbonyl)benzoate (31)

^{1}H (300 MHz, $CDCl_3$)

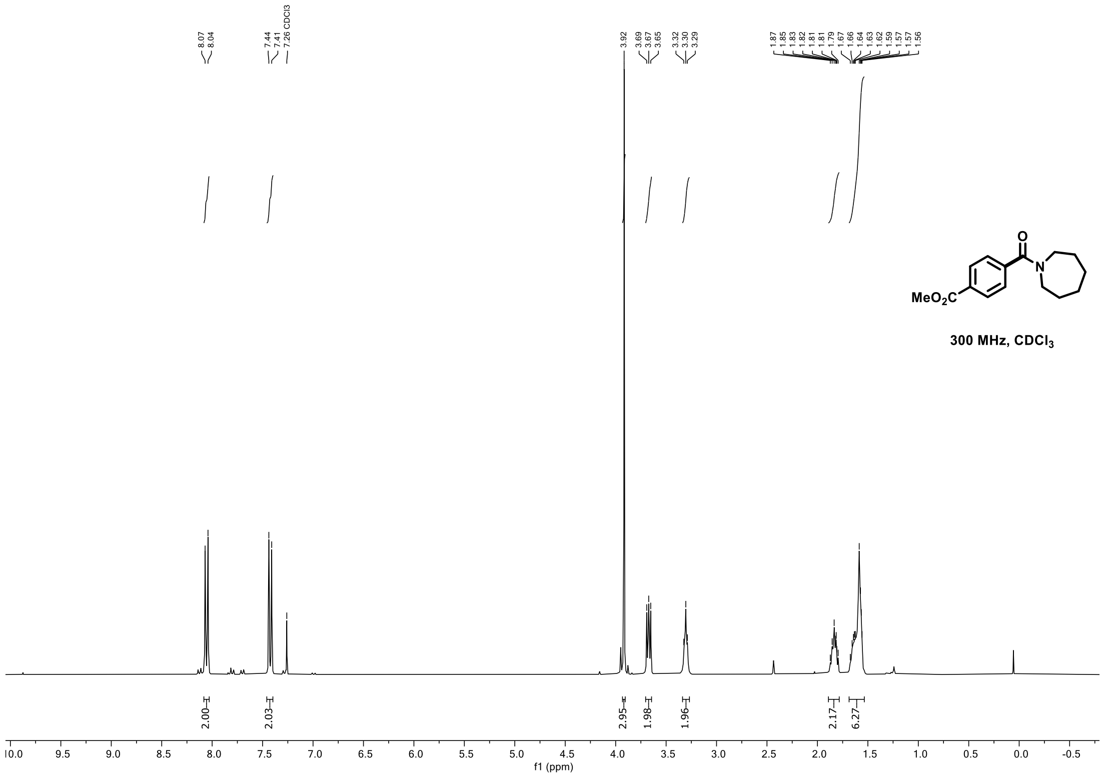

^{13}C (101 MHz, $CDCl_3$)

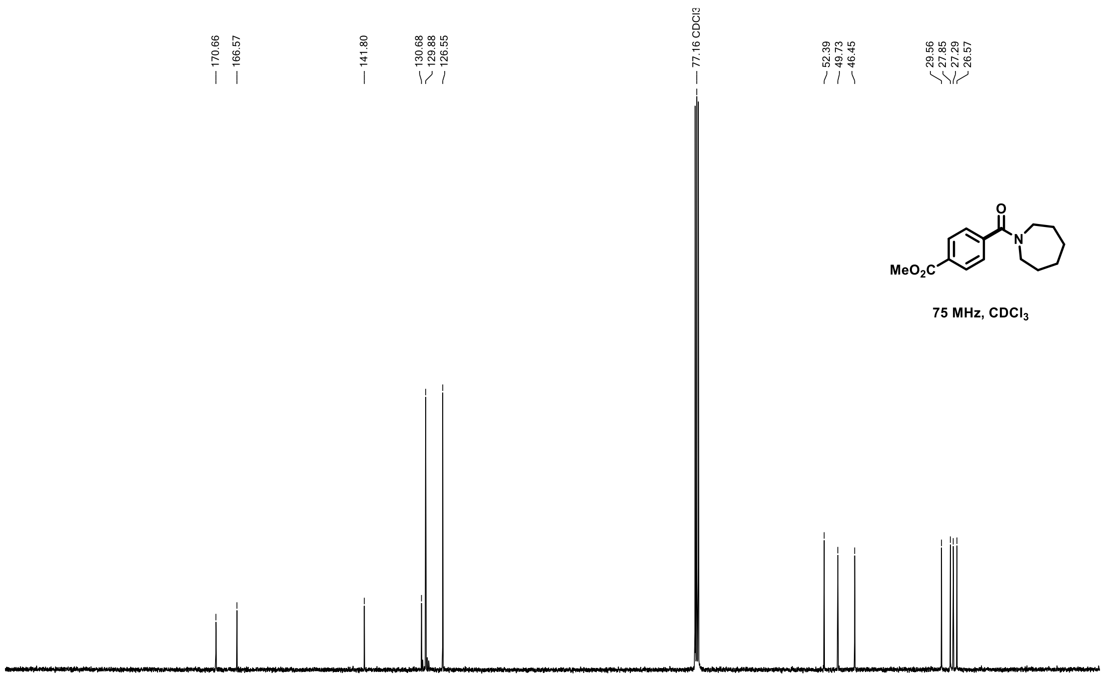

References

[1] T. M. Masson, S. D. Zondag, J. H. Schuurmans, T. Noël, *Reaction Chemistry & Engineering* **2024**, *9*, 2218-2225.

[2] H. Zeng, R. Yin, Y. Zhao, J.-A. Ma, J. Wu, *Nature Chemistry* **2024**, *16*, 1822-1830.

[3] M. U. Luescher, F. Gallou, *Green Chemistry* **2024**.

[4] U. Onken, A. Koettgen, H. Scheidat, P. Schueepp, F. Gallou, *Chimia* **2019**, *73*, 730-730.

[5] L. Chu, J. M. Lipshultz, D. W. MacMillan, *Angewandte Chemie International Edition* **2015**, *54*, 7929-7933.

[6] Y. Sun, N. Zhang, J. Ren, H. Huang, X. Luan, Z. Zuo, *Organic Letters* **2023**, *26*, 35-40.

[7] I. M. Ogbu, G. Kurtay, M. Badufle, F. Robert, C. S. Lopez, Y. Landais, *Chemistry–A European Journal* **2023**, *29*, e202202963.

[8] D. T. Mooney, B. D. Donkin, N. Demirel, P. R. Moore, A.-L. Lee, *The Journal of Organic Chemistry* **2021**, *86*, 17282-17293.

[9] S. Chand, A. K. Sharma, A. K. Pandey, K. N. Singh, *Chemical Communications* **2023**, *59*, 14827-14830.

[10] J. Zhang, X.-F. Wu, *Organic Letters* **2023**, *25*, 2162-2166.

[11] X. Wang, Y. Huang, Y. Xu, X. Tang, W. Wu, H. Jiang, *The Journal of Organic Chemistry* **2017**, *82*, 2211-2218.

[12] R. Ruzi, K. Liu, C. Zhu, J. Xie, *Nature Communications* **2020**, *11*, 3312.

[13] J. De Houwer, K. Abbaspour Tehrani, B. U. Maes, *ChemInform* **2012**, *43*, no.

[14] Rahul P, Veena S, J. John, *The Journal of Organic Chemistry* **2022**, *87*, 13708-13714.

[15] P. Boehm, P. Müller, P. Finkelstein, M. A. Rivero-Crespo, M.-O. Ebert, N. Trapp, B. Morandi, *Journal of the American Chemical Society* **2022**, *144*, 13096-13108.

[16] V. Murugesan, A. Muralidharan, G. V. Anantharaj, T. Chinnusamy, R. Rasappan, *Organic Letters* **2022**, *24*, 8435-8440.

[17] S.-S. Ma, B.-L. Jiang, Z.-K. Yu, S.-J. Zhang, B.-H. Xu, *Organic Letters* **2021**, *23*, 3873-3878.

[18] W. Ma, D. Xue, T. Yu, C. Wang, J. Xiao, *Chemical Communications* **2015**, *51*, 8797-8800.

[19] A. H. Dardir, P. R. Melvin, R. M. Davis, N. Hazari, M. Mohadjer Beromi, *The Journal of Organic Chemistry* **2018**, *83*, 469-477.

[20] G. Li, M. Szostak, *Chemistry–A European Journal* **2020**, *26*, 611-615.

[21] L. Yang, X. Dong, L. Wei, W. Chao, L. Zhao-Tie, X. Jianliang, *Org. Lett.* **2014**, *16*.

[22] B. Suchand, G. Satyanarayana, *The Journal of Organic Chemistry* **2016**, *81*, 6409-6423.

[23] C. Lei, D. Zhu, V. I. T. Tangcueco, J. S. Zhou, *Organic Letters* **2019**, *21*, 5817-5822.

[24] B. Chen, X.-F. Wu, *Organic Letters* **2019**, *21*, 7624-7629.

[25] F. Szabó, B. Pethő, Z. Gonda, Z. Novák, *RSC Advances* **2013**, *3*, 4903-4908.

[26] D. Marcos-Atanes, C. Vidal, C. D. Navo, F. Peccati, G. Jiménez-Osés, J. L. Mascareñas, *Angewandte Chemie International Edition* **2023**, *62*, e202214510.

[27] V. Vinayagam, T. V. Hajay Kumar, R. Nune, S. K. Karre, S. K. Sadhukhan, *The Journal of Organic Chemistry* **2023**, *88*, 2122-2131.

[28] P. Wang, S. M. Batt, B. Wang, L. Fu, R. Qin, Y. Lu, G. Li, G. S. Besra, H. Huang, *Journal of Medicinal Chemistry* **2021**, *64*, 6241-6261.

[29] H. Yuan, K. Su, M. Ji, H. Xue, H. Chen, Y. Zhang, *Synthesis* **2023**, *55*, 1586-1592.
